# Systematic literature review on Calcium Pyrophosphate Deposition (CPPD) nomenclature: condition elements and clinical states— A Gout, Hyperuricaemia and Crystal-Associated Disease Network (G-CAN) consensus project

**DOI:** 10.1136/rmdopen-2024-004847

**Published:** 2025-01-30

**Authors:** Charlotte Jauffret, Antonella Adinolfi, Silvia Sirotti, Daniele Cirillo, Luca Ingrao, Alessandro Lucia, Edoardo Cipolletta, Emilio Filippucci, Sara Tedeschi, Robert Terkeltaub, Nicola Dalbeth, Tristan Pascart, Georgios Filippou

**Affiliations:** 1 Rheumatology Department, Lille Catholic University, Saint Philibert Hospital, EA 7446 - ETHICS, Lille, France; 2 University of Lille, ULR 2694 – METRICS, CERIM, Lille, France; 3 Rheumatology unit, ASST Grande Ospedale Metropolitano Niguarda, Milano, Italy; 4 Rheumatology Department, IRCCS Galeazzi – Sant’Ambrogio Hospital, Milan, Italy; 5 Department of Clinical Sciences and Community Health, Università degli Studi di Milano, Milano, Italy; 6 Rheumatology Unit - Department of Clinical and Molecular Sciences, Polytechnic University of Marche, Ancona, Italy; 7 Academic Rheumatology, University of Nottingham, Nottingham, UK; 8 Division of Rheumatology, Inflammation and Immunity, Brigham and Women's Hospital, Boston, Massachusetts, USA; 9 Harvard Medical School, Boston, Massachusetts, USA; 10 Division of Rheumatology, Autoimmunity, and Inflammation, University of California San Diego, La Jolla, California, USA; 11 Department of Medicine, University of Auckland, Auckland, New Zealand; 12 Department of Biomedical and Clinical Sciences, Università degli Studi di Milano, Milano, Italy

**Keywords:** Chondrocalcinosis, Arthritis, Crystal arthropathies, Epidemiology

## Abstract

**Objectives:**

The Gout, Hyperuricaemia and Crystal-Associated Disease Network (G-CAN) has developed a calcium pyrophosphate deposition (CPPD) nomenclature project. This systematic literature review constituted its first step and aimed to provide a state-of-the-art analysis of the medical literature of the last 20 years.

**Methods:**

A systematic literature search was undertaken in the *PubMed*, *Cochrane* and *Embase* databases between 2000 and 2022, restricted to studies on humans and in the English language. Eight reviewers independently and manually extracted labels related to CPPD concepts, according to an a priori list generated by the authors: pathogenic conditions and pathogenic crystal labels, elementary imaging condition elements and asymptomatic and symptomatic condition states. For each concept, labels were analysed to determine their frequency.

**Results:**

Among the 2375 articles identified, 886 articles were included, of which 394 (44.5%) were case reports and 169 (19.0%) were scoping reviews. Overall, the most common labels used to designate the pathogenic condition were ‘*pseudogout’* in 365/783 (46.6%), ‘*chondrocalcinosis’* in 207/783 (26.4%) and ‘*calcium pyrophosphate deposition disease’* in 181/783 (23.1%) occurrences. The most common abbreviation was ‘CPPD’ in 312/390 (80.0%), but with different meanings. CPPD clinical phenotypes were often described as ‘pseudo-form’ labels.

**Conclusion:**

Those results demonstrate the heterogeneity of labels used to describe CPPD condition concepts, with wide variation in condition labels in the medical literature. This work provides the rationale and basis to achieve agreement about CPPD technical nomenclature.

**WHAT IS ALREADY KNOWN ON THIS TOPIC**

- The calcium pyrophosphate deposition (CPPD) nomenclature is characterised by a wide number of terms and labels that refer to similar aspects of the condition.
- This constitutes a major obstacle, both for quality research and for patient-doctor communication.

**WHAT THIS STUDY ADDS**

- This systematic literature review (SLR) collected all the labels and descriptions for the different aspects of CPPD that have been used in the last 22 years and demonstrated the enormous variability of the terms used to describe the condition.

**HOW THIS STUDY MIGHT AFFECT RESEARCH, PRACTICE OR POLICY**

- This SLR is the basis of a G-CAN project that aims to develop a consensus terminology for all aspects of the disease. The data collected in this SLR will be used in the next steps of the project.
- This SLR is expected to raise awareness regarding the need to standardise the terms and definitions of CPPD.

## Introduction

 Calcium pyrophosphate deposition (CPPD)^1^ is a pathogenic condition related to the presence of calcium pyrophosphate (CPP) crystal deposits in articular and periarticular tissues that can be asymptomatic CPPD or symptomatic CPPD disease.^2^,^4^

In 2019, a review highlighted the challenges in describing CPPD,^5^ despite prior work to standardise this terminology.^2^ In 1985, Ryan and McCarty recognised five phenotypes of a general disease called ‘*calcium pyrophosphate dihydrate (CPPD) crystal deposition disease’: ‘asymptomatic (lanthanic) calcium pyrophosphate dihydrate crystal deposition’*, ‘*pseudo-osteoarthritis’*, ‘*pseudogout’*, ‘*pseudo rheumatoid arthritis’* and ‘*pseudoneuropathic joints’* and also some rare phenotypes.^6^ Then, the proposed terminology by the European League Against Rheumatism (EULAR) in 2011 recognised the term ‘*calcium pyrophosphate deposition (CPPD)’* as the umbrella term for all instances of CPP crystal occurrence, while the cartilage calcification was defined as ‘chondrocalcinosis (CC)’. EULAR recognised four phenotypes, abandoning the prefix ‘pseudo’ to reduce confusion. However, there is ongoing imprecision in differentiating between asymptomatic and symptomatic forms.^7^ Thus, clinicians and investigators are currently faced with a variety of terms (eg, calcium pyrophosphate deposition disease, chondrocalcinosis, pseudogout and pyrophosphate arthropathy) that have been used and are still used interchangeably to describe different pathologic features and clinical states of the condition.^5 7^

On the heels of growing collective awareness over the past few years, CPPD is now a focus of international research efforts^8^,^10^; thus, the use of standardised definitions for the various elements and states of a pathogenic condition is a priority to ensure clear communication, both in clinical practice and research.^11^ Therefore, the Gout, Hyperuricaemia and Crystal-Associated Disease Network (G-CAN), an international group of rheumatologists, other physicians and non-clinician scientists that share interest and expertise in crystal arthropathies, has established a CPPD Nomenclature Project to achieve international consensus about the nomenclature of CPPD. This project is in line with a previous G-CAN endeavour on labels and definitions of condition elements and states of gout in 2018,^12 13^ which had a positive impact on the homogenisation of the language and concepts used in gout in the following years.^11^ As the first step, the project team planned this systematic literature review (SLR) to create a content analysis of the medical literature over the last two decades and to identify labels, abbreviations and definitions for CPPD, its basic elements and clinical state features.

## Methods

The Preferred Reporting Items for Systematic Reviews and Meta-analyses 2020 guidelines for reporting systematic reviews and meta-analyses were followed for this review.^14^

### Structured search strategy

The systematic research was undertaken through PubMed and Embase databases and the Cochrane Library, starting from 1 January 2000 to 31 August 2022.

Three sub-questions were developed:

- The first question aimed to assess the labels used to represent pathogenic condition and pathogenic crystal names.
- The second question aimed to assess the labels used to represent pathogenic crystal deposition elements and pathogenic condition imaging elements.
- The third question aimed to assess labels used to represent clinical condition states.

The search terms are summarised in online supplemental table S1.

### Study selection and data extraction

The search included all the studies that were in thee English language, limited to humans and with availability full text. The following study types were eligible for inclusion: SLR, meta-analysis, randomised controlled trial, retrospective or longitudinal or cross-sectional cohort study, retrospective or prospective or longitudinal case-control study, case series, case report, scoping review and letter to the editor. Studies were excluded in case of duplication, non-English language, unavailable full text, non-human population and non-included study type.

The titles, abstracts and text bodies were independently screened and then included by eight reviewers (AA, SS, CJ, EC, DC, LI, AL and EF) according to the pre-defined inclusion and exclusion criteria. The articles were randomly divided in equal numbers to the reviewers, and each one of them screened and then extracted the data only from the assigned papers. Before extraction, calibration was undertaken using a random sample of three articles, followed by a dummy extraction by the eight reviewers (AA, SS, CJ, EC, DC, LI, AL and EF) and a data quality assessment by GF. An interim quality assessment was performed during the data extraction by GF and during the analysis by CJ. Any disagreements were resolved by consensus. Data were manually extracted from the text, figures and tables, using a standardised extraction form on the *REDCap* tool, a solution allowing manual entry of information into a standardised form. Labels were extracted verbatim from the examined text. Modalities of a variable were entered in the extraction form only at their first occurrence in the article. In case of missing data, the box on the standard form was left blank.

For each article, the title, journal, first author, publication year, study type, the clinical state of the condition (if symptomatic or not), the diagnostic criteria used (McCarty criteria, synovial fluid analysis, imaging, histology, expert opinion) and the imaging applied, if available (conventional radiology, ultrasonography, CT, dual-energy CT, MRI, others), were recorded (see online supplemental table S2).

The data about pathogenic condition and pathogenic crystal names and abbreviations, imaging condition elements and clinical condition states were recorded according to an a priori list, generated by the authors before the start of the SLR and updated during data collection if appropriate.

Extracted labels were recorded verbatim, according to the following concepts:

- Pathogenic condition: name, abbreviation and abbreviation meaning
- Pathogenic crystal: name and abbreviation
- Crystal deposition elements: the macroscopic deposition of pathogenic crystal in tissues, the microscopic deposition of pathogenic crystal in tissues and the presence of pathogenic crystal in synovial fluid analysis (SFA).
- Imaging condition elements: evidence of pathogenic crystal deposition on conventional X-ray, on US, on CT, on MRI and on DECT; imaging evidence of structural cartilage damage due to pathogenic crystal deposits.
- Asymptomatic evidence of pathogenic crystal deposition: on SFA, on conventional radiography, on US and on CT scan.
- Symptomatic condition states: mono, oligo, polyarticular or periarticular nature of inflammation triggered by the presence of pathogenic crystals; acute peripheral articular inflammation triggered by the presence of pathogenic crystals; recurrent acute peripheral articular inflammation triggered by the presence of pathogenic crystals; persistent peripheral articular inflammation triggered by the presence of pathogenic crystals; skin and subcutaneous involvement with evidence of crystal deposits composed of calcium pyrophosphate; cervical spinal involvement with evidence of crystal deposits composed of calcium pyrophosphate; lumbar spinal involvement with evidence of crystal deposits composed of calcium pyrophosphate; symptomatic osteoarthritis (OA) related to crystal deposits composed of calcium pyrophosphate.

For this project, a condition state was defined as a clinically meaningful cluster based on rheumatology clinical practice experience, given the lack of consensus.

### Patient and public involvement

Patients or the public were not involved in the design, conduct, reporting or dissemination plans of this SLR as it was not deemed appropriate. The project includes a second step for the development of lay terms for the condition that will include a large number of patients and stakeholders.

### Assessment of the risk of bias

Given the descriptive nature of the data, there is no need to assess bias; therefore, no results will be presented on this issue.

### Data analysis

Among the included studies, only descriptive analyses were performed, as the great heterogeneity of the literature and the type of data extracted do not allow the implementation of a meta-analysis. It aimed to identify and extract terms indicating definitions, aetiology, pathogenesis, clinical presentation, imaging features and clinical states of CPPD as outlined above. Labels were taken verbatim, without setting up a grouping.

### Statistical analysis

As previously stated, the extracted labels were divided according to the three identified sub-questions.

For each sub-question of the extraction form, a certain quantity of labels that answered to the sub-question was obtained. For each one, the frequency of occurrence was calculated between the number of articles containing a particular label, and the total number of articles that presented at least one answer for the subquestion and then presented as a ratio and as a percentage. Because the information gathered was extremely varied, we chose to present only the modalities that were most widely reported in the literature.

All analyses were performed using R (4.1.2) and RStudio (2021.09.0 + 351) and the dplyr package.

## Results

### Descriptive results on the included articles

**Figure 1:**
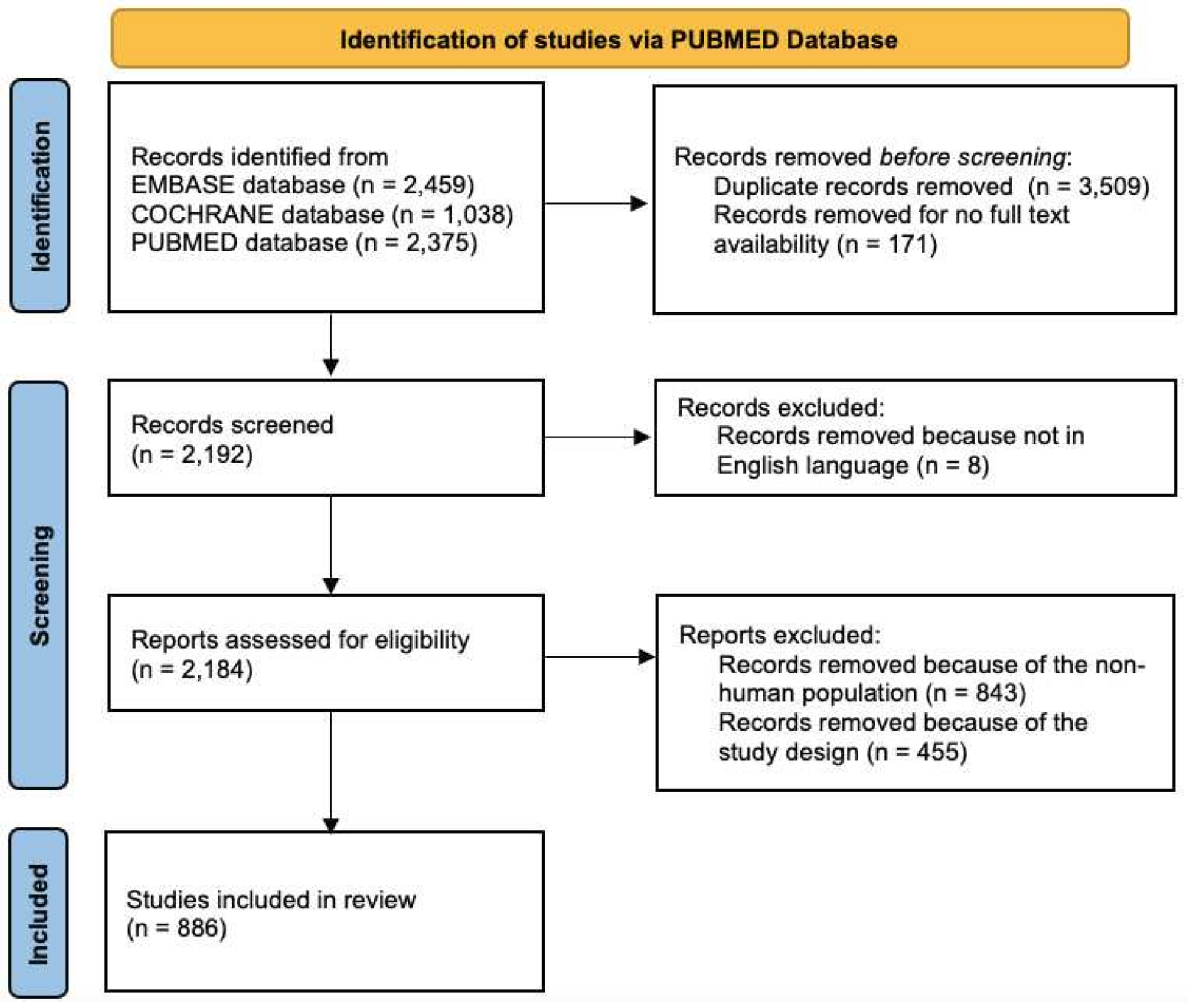
Preferred Reporting Items for Systematic Reviews and Meta-analyses 2020 flow diagram for new systematic reviews, which included searches of databases and registers only.

A total of 2375 articles were identified using the predefined search criteria. After the screening, 12 articles were excluded due to duplication, 171 because of unavailable full text, 8 because of the article language, 843 because they were not conducted on the human population and 455 because of study design outside the scope of the SLR protocol (figure 1).

The final analysis included 886 articles, for which the main details are summarised in online supplemental table S3. The references, the diagnostic modalities applied and the CPPD condition definitions proposed by the authors are illustrated in online supplemental table S4. Among those included articles, 394/886 (44.5%) were case reports and 169/886 (19.0%) were scoping reviews, and there were only 4/886 (0.5%) randomised controlled trials and only 3/886 (0.3%) meta-analyses.

Overall, the basis of CPPD diagnosis (alone or in combination with another modality) was the synovial fluid analysis in 272/886 (30.7%) articles, followed by imaging in 244/886 (27.5%), histology in 160/886 (18.1%) and McCarty criteria in 28/886 (3.2%). Expert opinion was expressed to support the diagnosis in 33/886 (3.7%).

The category ‘pathogenic condition and pathogenic crystal names’ was the one with the highest extracted-element rate with a maximum of 783/886 (88.4%) precisely for ‘condition name’. The extracted-element rate for the category ‘basic condition elements’ was at a maximum of 569/886 (64.2%) precisely for ‘imaging elements’. For the ‘clinical condition states’ category, it was 460/886 (51.9%) precisely for ‘symptomatic condition states’.

Among all the included articles, 266/886 (30.0%) provided a definition for the pathogenic condition. Available definitions were globally mentioning a probable high frequency of CPPD in the elderly, caused by intra-articular and periarticular deposition of calcium pyrophosphate crystals and characterised by heterogeneous presentations. Those sometimes included a list of clinically relevant phenotypes for the condition, like asymptomatic, acute, subacute and chronic forms.

In 405/886 (45.7%) of the included articles, ‘*CPPD’* was used to describe the deposition (both symptomatic and asymptomatic), and in 143/886 (16.1%), ‘*CPPD’* was used to describe symptomatic disease. Those results are detailed in online supplemental table S4.

### Labels used to represent pathogenic conditions and pathogenic crystal names

The labels and abbreviations used in the current literature to designate the pathogenic condition and the pathogenic crystal are summerised in table 1 and extensively presented in onlinesupplemental tables S5  S6. To designate the pathogenic condition, ‘*pseudogout’* was the most used term with 365/783 (46.6%) occurrences, followed by ‘*chondrocalcinosis’* with 207/783 (26.4%) occurrences and finally ‘*calcium pyrophosphate deposition disease’* with 181/783 (23.1%) occurrences. The pathogenic condition abbreviation was mostly ‘*CPPD’* with 312/390 (80.0%) occurrences, followed by ‘*CPDD’* with 38/390 (9.7%) occurrences and ‘*CC’* with 31/390 (7.9%) occurrences. The pathogenic condition abbreviation meaning in those cases was ‘*calcium pyrophosphate deposition disease’* in 104/421 (24.7%) cases, followed by ‘*calcium pyrophosphate dihydrate’* in 73/421 (17.3%) and finally ‘*calcium pyrophosphate deposition’* in 72/421 (17.1%).

**Table 1:** Labels used to represent the concepts of pathogenic condition (name, abbreviation and abbreviation meaning) and pathogenic crystal (name and abbreviation), among all article types, between 2000 and 2022

Concept	Articles in which at least one label is available for the concept, N (%)	Total number of labels available for the concept, N	Most commonly used labels in the literature, N (%)*
The pathogenic condition			
Pathogenic condition name	783/886 (88.4)	64	Pseudogout, 365/783 (46.6) Chondrocalcinosis, 207/783 (26.4) Calcium pyrophosphate deposition disease, 181/783 (23.1)
Pathogenic condition abbreviation	390/886 (44.0)	16	CPPD, 312/390 (80.0) CPDD, 39/390 (10.0) CC, 31/390 (7.9)
Pathogenic condition abbreviation meaning	421/886 (47.5)	32	Calcium pyrophosphate deposition disease, 104/421 (24.7) Calcium pyrophosphate dihydrate, 73/421 (17.3) Calcium pyrophosphate deposition, 72/421 (17.1)
The pathogenic crystal			
Pathogenic crystal name	717/886 (80.9)	10	Calcium pyrophosphate dihydrate, 372/717 (51.9) Calcium pyrophosphate, 280/717 (39.1) Calcium pyrophosphate dehydrate, 61/717 (8.5)
Pathogenic crystal abbreviation	551/886 (62.2)	12	CPPD, 335/551 (60.8) CPP, 213/551 (24.0) CCP, 3/551 (0.3)

N (%), effective (proportion).

* For more detailed results, please see supplementary material.

CPPcalcium pyrophosphateCPPDcalcium pyrophosphate dihydrate

The most frequently used crystal label was ‘*calcium pyrophosphate dihydrate’* in 372/717 (51.9%) articles, followed by ‘*calcium pyrophosphate’* in 280/717 (39.1%) and ‘*calcium pyrophosphate dehydrate’* in 61/717 (8.5%). The crystal abbreviation was mainly ‘*CPPD’* founded in 335/550 (60.9%) articles, then ‘*CPP’* in 213/550 (24.0%) articles and ‘*CCP’* in 3/550 (0.3%).

To have access to the crude results of the analysis, please see online supplemental table S7.

### Labels used to represent crystal deposition and imaging condition elements

Whether in tissues or in imaging, the terminologies used to describe crystal deposits are quite similar and are presented in table 2.

**Table 2:** Labels used to represent crystal deposition and imaging pathogenic condition elements, among all article types, between 2000 and 2022

Concept	Articles in which at least one label is available for the element concept, N (%)	Total number of unique labels available for the concept, N	Most commonly used labels in the literature, N (%)*
Crystal deposition elements			
The macroscopic deposition of pathogenic crystal in tissues	76/886 (8.6)	19	Material, 23/76 (30.3) Mass, 21/76 (27.6) Deposit, 10/76 (13.2)
The microscopic deposition of pathogenic crystal in tissues	162/886 (18.3)	19	Crystal, 94/162 (58.0) Deposit, 41/162 (25.3) Calcification, 16/162 (9.9)
The presence of pathogenic crystal in SFA	213/886 (24.0)	6	Crystal, 210/213 (98.6) Deposit, 1/213 (0.5) Chondrocalcinosis, 1/213 (0.5)
Imaging pathogenic condition elements			
Evidence of pathogenic crystal deposition on CR	399/886 (45.0)	19	Chondrocalcinosis, 285/399 (71.4) Calcification, 139/399 (34.8) Deposit, 17/399 (4.3)
Evidence of pathogenic crystal deposition on US	89/886 (10.0)	11	Deposit, 68/89 (76.4) Calcification, 15/89 (16.9) Chondrocalcinosis, 7/89 (7.9)
Evidence of pathogenic crystal deposition on CT	205/886 (23.1)	32	Calcification, 122/205 (59.5) Calcified mass, 19/205 (9.3) Chondrocalcinosis, 19/205 (9.3)
Evidence of pathogenic crystal deposition on MRI	72/886 (8.1)	16	Mass, 30/72 (41.7) Calcification, 17/72 (23.6) Chondrocalcinosis, 10/72 (13.9)
Evidence of pathogenic crystal deposition on DECT	13/886 (1.5)	9	Calcification, 5/13 (38.5) Calcium deposition, 2/13 (15.4) Calcium pyrophosphate crystal deposition, 1/13 (7.7)
Imaging evidence of structural cartilage damage due to pathogenic crystal deposits	Not collected†	Not collected†	Not collected†

* For more detailed results, please see supplementary material.

† Please refer to the discussion.

(%), effective (proportion); CR, conventional radiography; CT, computed tomography; DECT, dual-energy CTMRI, magnetic resonance imaging; SFA, synovial fluid analysis; US, ultra-sonography

The macroscopic deposition of pathogenic crystal in tissues was described as a ‘*material’* in 23/76 (30.3%) articles, as a mass in 21/76 (27.6%), and as a deposit in 10/76 (13.2%). Microscopically in tissues, the deposition of pathogenic crystal was described as a ‘*crystal’* in 94/162 (58.0%), a ‘*deposit’* in 41/162 (25.3%) and a ‘*calcification’* in 16/162 (9.9%). Finally, in synovial fluid analysis, the presence of pathogenic crystal was mainly described as a ‘*crystal’* in 210/213 (98.6%), a ‘*deposit’* in 1/213 (0.5%) and ‘*chondrocalcinosis’* in 1/213 (0.5%).

Conventional radiography (CR) and CT were the most described imaging modalities, with 399/886 (45.0%) and 205/886 (23.1%) articles, respectively, proposing at least one label for the concept. On CR, most authors used the term ‘*chondrocalcinosis’* that was found in 285/399 (71.4%) articles, ahead of ‘*calcification’* in 139/399 (34.8%) and ‘*deposit’* in 17/399 (4.3%). On ultrasonography (US), imaging basic element was mostly described as a ‘*deposit’* in 68/89 (76.4%) articles, then as a ‘*calcification’* in 15/89 (16.9%) and as ‘*chondrocalcinosis’* in 7/89 (7.9%).

On CT, pathogenic crystal deposits were named ‘*calcification’*, ‘*calcified mass’* and ‘*chondrocalcinosis’*, respectively, in 122/205 (59.5%), 19/205 (9.3%) and 19/205 (9.3%) articles. On MRI, authors mostly used the term ‘*mass’*, found in 30/72 (41.7%) articles, ahead of ‘*calcification’* found in 17/72 (23.6%) and ‘*chondrocalcinosis’* found in 10/72 (13.9%). Dual-energy computed tomography (DECT) deposits were described as ‘*calcification’* in 5/13 (38.5%), ‘*calcium deposition’* in 2/13 (15.4%) and ‘*calcium pyrophosphate crystal deposition’* in 1/13 (7.7%). Raman spectra, technetium-99 bone scan, FDG PET/CT and SPECT/CT were infrequently used and described.

The crude results of the analysis are presented in online supplemental table S8.

### Labels used to represent clinical condition states

#### Asymptomatic condition states

Asymptomatic condition states were infrequently described, and their available labels mainly correspond to CR deposits, under the labels ‘*chondrocalcinosis’* in 15/38 (39.5%) articles and ‘*asymptomatic chondrocalcinosis’* in 11/38 (28.9%) articles. This was never mentioned among ultrasound results.

#### Symptomatic condition states

Regarding pathogenic condition states, the acute peripheral form was described in 243/886 (27.4%) articles, the recurrent acute peripheral form in 46/886 (5.2%) and the persistent peripheral form in 88/886 (9.9%). Skin and subcutaneous involvement was described in 13/886 (1.5%), cervical spinal involvement in 162/886 (18.3%) and lumbar spinal involvement in 18/886 (2.0%). Finally, symptomatic OA related to crystal deposits composed of calcium pyrophosphate was described in 119/886 (13.4%).

The most used labels to describe one episode of acute peripheral articular inflammation were ‘*pseudogout’* in 113/243 (46.5%) articles, ‘*acute CPP crystal arthritis’* in 52/243 (21.4%) and ‘*acute pseudogout’* in 22/243 (9.1%). The recurrence of an acute peripheral articular inflammation was also described as ‘*pseudogout’* in 16/46 (34.8), ‘*acute recurrent CPP arthritis’* in 4/46 (8.7) and ‘*pseudo-rheumatoid arthritis’* in 3/46 (6.5). To describe a persistent peripheral articular inflammation, ‘*chronic CPP crystal arthritis’* was found in 27/88 (30.7%), followed by ‘*pseudo-rheumatoid arthritis’* in 25/88 (28.4%) and chronic arthritis in 15/88 (17.0%). For skin and subcutaneous involvement with evidence of pathogenic crystal deposits, ‘*tophaceous pseudogout’* was used in 25/49 (51.0%), ‘*tophaceous CPPD’* in 5/49 (10.2%), and ‘*tumorous CPPD’* in 4/49 (8.2%). For spinal involvement, at the cervical level, labels were mainly ‘*crowned dens syndrome’* in 122/162 (75.3%), followed by ‘*cervical cord compression/myelopathy’* in 24/162 (14.8%) and ‘*cervical axial pain’* in 20/162 (12.3%). At the lumbar level, labels were mainly ‘*lumbar axial pain’* in 7/18 (38.9%), ‘*radiculopathy’* in 6/18 (33.3%) and ‘*cauda equina syndrome’* in 3/18 (16.7%). Finally, the most common labels used to identify symptomatic OA related to pathogenic crystal deposits were ‘*CPPD-related/associated OA’* in 37/119 (31.1%), ‘*pseudo-osteoarthritis’* in 25/119 (21.0%) and ‘*CPP crystal deposits with OA’* in 15/119 (12.6%).

Data were not collected for mono, oligo, polyarticular or periarticular inflammation triggered by the presence of pathogenic crystals, because this concept emerged during the discussion of the results by the steering committee after the end of the data extraction.

A summary of the results is presented in table 3. The crude results of the analysis are presented in online supplemental table S9.

**Table 3:** Labels used to represent clinical condition states, among all article types, between 2000 and 2022

Concept	Articles in which at least one label is available for the concept, N (%)	Total number of labels available for the concept, N	Most commonly used labels in the literature, N (%)*
Asymptomatic condition states			
Asymptomatic evidence of pathogenic crystal deposition on SFA	3/886 (0.3)	2	Calcium pyrophosphate crystals in aspiration from asymptomatic joints, 2/3 (66.6) Calcium pyrophosphate crystals in aspiration from asymptomatic joints during inter-critical periods, 1/3 (33.3)
Asymptomatic evidence of pathogenic crystal deposition on CR	38/886 (4.3)	17	Chondrocalcinosis, 15/38 (39.5) Asymptomatic chondrocalcinosis, 11/38 (28.9) Incidental radiographic finding, 2/38 (5.3)
Asymptomatic evidence of pathogenic crystal deposition on US	0/886	NA	NA
Asymptomatic evidence of pathogenic crystal deposition on CT scan	2/886 (0.2)	2	Asymptomatic calcification, 1/2 (50.0%) Asymptomatic calcium deposition, 1/2 (50.0%)
Symptomatic condition states			
Mono, oligo, polyarticular or periarticular inflammation triggered by the presence of pathogenic crystals	Not collected†	Not collected†	Not collected†
Acute peripheral articular inflammation triggered by the presence of pathogenic crystals	243/886 (27.4)	37	Pseudogout, 113/243 (46.5) Acute CPP crystal arthritis, 52/243 (21.4) Acute pseudogout, 22/243 (9.1)
Recurrent acute peripheral articular inflammation triggered by the presence of pathogenic crystals	46/886 (5.2)	19	Pseudogout, 16/46 (34.8) Acute recurrent CPP arthritis, 4/46 (8.7) Pseudo-rheumatoid arthritis, 3/46 (6.5)
Persistent peripheral articular inflammation triggered by the presence of pathogenic crystals	88/886 (9.9)	28	Chronic CPP crystal arthritis, 27/88 (30.7) Pseudo-rheumatoid arthritis, 25/88 (28.4) Chronic arthritis, 15/88 (17.0)
Skin and subcutaneous involvement with evidence of pathogenic crystal deposits	49/886 ()	11	Tophaceous pseudogout, 25/49 (51.0) Tophaceous CPPD, 5/49 (10.2) Tumorous CPPD, 4/49 (8.2)
Cervical spinal involvement with evidence of pathogenic crystal deposits	162/886 (18.3)	51	Crowned dens syndrome, 122/162 (75.3) Cervical cord compression/myelopathy, 24/162 (14.8) Cervical axial pain, 20/162 (12.3)
Lumbar spinal involvement with evidence of pathogenic crystal deposits	18/886 (2.0)	17	Lumbar axial pain, 7/18 (38.9) Radiculopathy, 6/18 (33.3) Cauda equina syndrome, 3/18 (16.7)
Symptomatic OA related to pathogenic crystal deposits	119/886 (13.4)	45	CPPD-related/associated osteoarthritis, 37/119 (31.1) Pseudo-osteoarthritis, 25/119 (21.0) CPP crystals deposits with osteoarthritis, 15/119 (12.6)

* For more detailed results, please see Online supplemental table 6supplementary material.

† Please refer to the discussion.

CRconventional radiographyCTcomputed tomographyN (%)effective (proportion)NAnon-applicableOAosteoarthritisSFAsynovial fluid analysisUSultra-sonography

## Discussion

CPPD is one of the most challenging conditions to assess as the presence of CPP deposition may be associated (or in overlap?) with other arthropathies,^15^,^17^ making symptom attribution difficult and final diagnosis fallible. The increasing importance of CPPD, particularly in the setting of the ageing population, makes it necessary to establish international consensus on CPPD nomenclature, as a preliminary step to improve research on this field. The results of this SLR further pinpoint how far we are from consistent terminology in CPPD, as over the last two decades the terminology used in the literature is extremely varied, and same labels often refer to different concepts.

The condition’s label constitutes one of the most striking controversies. Indeed, its designations as ‘chondrocalcinosis’ (ie, radiographic deposits) or ‘pseudogout’ (ie, former term for acute arthritis due to CPPD), have been widely used, whereas the last recognised umbrella term in the EULAR recommendations is ‘calcium pyrophosphate deposition’ (the current term for all clinical phenotypes of CPPD).^2^ The other major confusion concerns the recognition of CPPD as a deposition (covering asymptomatic and symptomatic forms) or as a disease (only symptomatic forms). This confusion is revealed through letter ‘D’ of the ‘CPPD’ abbreviation meaning—that sometimes stands for ‘deposition’ and sometimes for ‘disease’. For this reason in this SLR, we decided to adopt the term ‘CPPD condition' to refer to all aspects of CPPD (asymptomatic and symptomatic) and to use 'CPPD disease' to refer only to the symptomatic form. A way to avoid confusion and inaccuracies in the literature should agree on a label for the asymptomatic form, because the asymptomatic form is often included under the terminology 'CPPD disease'. Finally, there was also a marked confusion with the abbreviation ‘CPPD’ that designate either the condition and the crystal, whereas the admitted crystal abbreviation is ‘CPP’ for ‘calcium pyrophosphate’.^2^ Regarding crystal labels, confusion has been made in the literature on the use of dihydrate and dehydrate for the name of the crystal: dehydrate is a wording error, and dihydrate is the correct name of the chemical compound according to the PubChem library.^18^

Considering the basic condition elements, one of the main issues is the place of the term ‘chondrocalcinosis’ that has been used variably to alternatively the condition, the deposits on CR, but also deposition in tissues and SFA, whereas it may be better used as a condition biomarker. For imaging modalities other than CR, ‘calcification’ was commonly used. There were numerous cervical CT scan results reported, because indeed numerous cervical axial clinical forms were described in case reports and case series. Finally, emerging imaging modalities such as the US and DECT were poorly described; these modalities will undoubtedly occupy a more prominent position in the literature over the next few years.

Considering clinical condition states, the acute, chronic and axial forms were the most frequently described. Acute and chronic forms were still often described as ‘pseudo forms’, although these were abandoned in the EULAR 2011 recommendations.^2^ The asymptomatic form was occasionally referred to as ‘chondrocalcinosis’, mainly because asymptomatic deposits were mainly identified by CR. Given the inclusion of a large number of case reports, many of which were referred to the axial form, we found numerous ‘crowned dens syndrome’ descriptions with a high level of agreement on that label. The recurrent form and subcutaneous and skin deposit-associated form were rarely mentioned. The subcutaneous and skin deposit-associated form was often described as a ‘tophaceous’ form, copying the gout nomenclature to describe the poorly codified phenotypes of CPPD disease.

The lack of a standardised nomenclature for disease course states was apparent in this study, because the most prevalent phenotypes were not clearly defined or were still pseudo-forms (eg, acute, recurrent, persistent and chronic forms, OA-associated form), and the less prevalent phenotypes were still partly ignored (eg, crowned dens syndrome, tophaceous form). Finally, one of the most controversial issues concerns the OA status among clinical condition elements: the pathophysiology between OA and CPPD and whether OA is a cause and/or a consequence of CPPD.^19^ Consistent with the EULAR recommendations,^2^ this combination is described sometimes as an entity itself and sometimes just as a possible association with the asymptomatic form^8 10^ (see below).

These findings indicate that previous attempts to standardise CPPD nomenclature have been unsuccessful. It should be underlined that this imprecision is supported by the insufficiency of coding systems.^5 7^ The most widely used is ICD-10, among which various associations of imprecise codes have been used to constitute heterogeneous study cohorts, namely ‘familial chondrocalcinosis (M111*)’, ‘other chondrocalcinosis (M112*)’, ‘other specified crystal arthropathies (M118*)’, ‘crystal arthropathy in other metabolic disorder (M141*)’ and ‘other disorders of calcium metabolism (E83.59*)’. The ICD11 coding system, currently in development, may be more precise with the inclusion of specific codes for CPPD, although with terminology that deviates from the EULAR recommendations, but hopefully, future versions will implement the upcoming consensus nomenclature on CPPD.^7 20^ Awaiting a better codification, the authors have recently developed algorithms, to better identify CPPD and its acute clinical form, which may constitute the best way to improve research on its clinical phenotypes. Those showed a good reliability and reproducibility in a Veterans Affairs study in 2015,^21^ and in a recent work on the Partners HealthCare Research Patient Data Repository.^22^

Our study has multiple strengths. Three databases were searched, so the greatest part of the literature has probably been covered. Due to the low number of publications available in this field over the years, we did not restrict the review to articles of high scientific quality only (notably no caution on journal types) nor limit strictly the period of inclusion, which allowed many included publications. This provides full representation of the contemporary literature. The search strategy reflected an attempt to be exhaustive in the screening of available publications. Further, we highlight the fact that eight reviewers did the inclusions, the calibration before inclusions and the quality controls implemented during and after inclusions. Despite the inequal proportions in terms of article types, the results per article and the pooled results did not show heterogeneity. The differences in extracted-element rates are explained by the fact that some variables are more frequently covered than others in articles and do not constitute a limitation from a lack of coverage.

The present SLR has also some limitations. Searches that use key words—however numerous they are—are at risk of unintentional data omission, so we cannot guarantee that our work is reflecting all the available data. The proportions of the different article types included are not balanced, but considering the qualitative nature of this work, the authors underline that this point is not a major limitation. The manual extraction of pathogenic condition elements necessarily led to subjective results, ie, has involved the reviewer’s assessment when reading articles during the inclusion process. The ‘not collected’ line that appears in table 2 is explained by the fact that imaging evidence of structural cartilage damage due to pathogenic crystal deposits is difficult to differentiate from primary OA descriptions. The ‘not collected’ line that appears in table 3 is explained by the fact that this concept was discussed by the steering committee after inclusion and because of the exploratory nature of the study.

Future analysis will be performed to evaluate the impact of the 2011 EULAR recommendations for CPPD terminology and diagnosis on the variability of the terminology.

Based on the findings from this SLR, experts in CPPD from the G-CAN membership will be invited to participate in Delphi exercises, to reach agreement on condition’s state labels and definitions for use in technical and scientific communications. Subsequently, this group will develop an easily understandable statement describing CPPD in a language that can be used in conversations with the lay public, media, non-governmental organisations, funders, healthcare providers and other stakeholders (common language definition).

To conclude, contemporary CPPD nomenclature is characterised by large heterogeneity and a lack of precision in the labels used to describe all aspects of the condition. The development of agreed nomenclature would positively impact both research quality and clinical practice. The current work provides the basis for agreement regarding the labels and definitions of this condition.

## supplementary material

10.1136/rmdopen-2024-004847 online supplemental table 1

10.1136/rmdopen-2024-004847 online supplemental table 2

10.1136/rmdopen-2024-004847 online supplemental table 3

10.1136/rmdopen-2024-004847 online supplemental table 4

10.1136/rmdopen-2024-004847 online supplemental table 5

10.1136/rmdopen-2024-004847 online supplemental table 6

10.1136/rmdopen-2024-004847 online supplemental table 7

10.1136/rmdopen-2024-004847 online supplemental table 8

10.1136/rmdopen-2024-004847 online supplemental table 9

## Data Availability

Data are available upon reasonable request.
