# Supplementary material for: Systematic literature review on Calcium Pyrophosphate Deposition (CPPD) nomenclature: condition elements and clinical states— A Gout, Hyperuricaemia and Crystal-Associated Disease Network (G-CAN) consensus project
Source: RMD Open. 2025 Jan 30;11(1):e004847. doi: 10.1136/rmdopen-2024-004847 (PMC11784236; doi:10.1136/rmdopen-2024-004847)
Supplement: online supplemental table 1 [file rmdopen-11-1-s001.pdf]

Supplementary Table S1. Search terms among databases

PubMed	Embase	Cochrane Library
1. Calcium pyrophosphate [Mesh Term]	1. Calcium pyrophosphate (Article title, Abstract, Keywords)	1. calcium pyrophosphate (All text)
2. Calcium pyrophosphate dihydrate (all fields)	2. Calcium pyrophosphate dihydrate (Article title, Abstract, Keywords)	2. calcium pyrophosphate dihydrate (All text)
3. Chondrocalcinosis [Mesh Term]	3. Chondrocalcinosis (Article title, Abstract, Keywords)	3. Chondrocalcinosis (All text)
4. Pseudogout (all fields)	4. Pseudogout (Article title, Abstract, Keywords)	4. Pseudogout (All text)
5. pseudo-gout (all fields)	5. pseudo-gout (Article title, Abstract, Keywords)	5. pseudo-gout (All text)
6. CPPD (all fields)	6. CPPD (Article title, Abstract, Keywords)	6. CPPD (All text)
7. CPDD (all fields)	7. CPDD (Article title, Abstract, Keywords)	7. CPDD (All text)
8. Pyrophosphate arthropathy (all fields)	8. Pyrophosphate arthropathy (Article title, Abstract, Keywords)	8. Pyrophosphate arthropathy (All text)
9. Crowned dens syndrome (all fields)	9. Crowned dens syndrome (Article title, Abstract, Keywords)	9. Crowned dens syndrome (All text)
10. Cartilage calcification (all fields)	10. cartilage calcification (Article title, Abstract, Keywords)	10. cartilage calcification (All text)

Limits: humans, English language
Time Period: January 1, 2000 to August 31, 2022
