# Supplementary material for: Systematic literature review on Calcium Pyrophosphate Deposition (CPPD) nomenclature: condition elements and clinical states— A Gout, Hyperuricaemia and Crystal-Associated Disease Network (G-CAN) consensus project
Source: RMD Open. 2025 Jan 30;11(1):e004847. doi: 10.1136/rmdopen-2024-004847 (PMC11784236; doi:10.1136/rmdopen-2024-004847)
Supplement: online supplemental table 2 [file rmdopen-11-1-s002.docx]

**Supplementary Table S2. RedCap extraction form.**

| **General informations** | TITLE | *(manual entry)* |
| --- | --- | --- |
|  | JOURNAL | *(manual entry)* |
|  | FIRST AUTHOR | *(manual entry)* |
|  | Publication year | *(manual entry)* |
|  | Investigator (simple choice answer) | Antonella Adinolfi  Silvia Sirotti  Edoardo Cipolletta  Emilio Filippucci  Charlotte Jauffret  Daniele Cirillo  Luca Ingrao  Alessandro Lucia |
|  | Included | No/Yes |
|  | If Not included (simple choice answer) | Duplication  Unavailable full text  Article language  Not on human population  Study design outside the scope of the SLR protocol |
|  | Study design (simple choice answer) | Systematic literature review  Meta-analysis  Randomized control trial (RCT)  Cohort study retrospective  Cohort study longitudinal  Cohort study cross-sectional  Case-control study retrospective  Case-control study longitudinal  Case-control study retrospective  Case series  Case report  Scoping review |
|  | CPPD diagnostic criteria (multiple choice answer) | McCarty criteria  Synovial fluid analysis  Imaging (specify)  Histology  Expert opinion  Other (specify) |
| **SB_1: Calcium Pyrophosphate Deposition Disease - Labels and Definitions** | CPPD disease Label | *(manual entry)* |
|  | CPPD disease Abbreviation | *(manual entry)* |
|  | CPPD disease Abbreviation Meaning | *(manual entry)* |
|  | CPPD crystal Label | *(manual entry)* |
|  | CPPD crystal Abbreviation | *(manual entry)* |
|  | Is CPPD disease designating (simple choice answer) …? | The deposition (both symptomatic and asymptomatic)  The disease (only symptomatic) |
|  | CPPD disease Definition | *(manual entry)* |
| **SB_2: to identify the disease elements of calcium pyrophosphate deposition disease present in the literature, and the labels used to represent them** | Pathogenetic elements: Yes/No (if yes, *manual entry*) | Macroscopic aspect of crystals in tissues  Calcium pyrophosphate crystals in synovial fluid analysis  Calcium pyrophosphate crystal deposits in tissues (microscopy or other high sensitivity techniques)  Other |
|  | Imaging elements: Yes/No (if yes, *manual entry*) | Abnormalities on CR due to CPPD  Abnormalities on Ultrasound due to CPPD  Abnormalities on CT due to CPPD  Abnormalities on DECT due to CPPD  Abnormalities on MRI due to CPPD  Other |
| **SB_3: to identify the clinical states of calcium pyrophosphate deposition disease present in the literature, and the labels used to represent them** | Asymptomatic/Preclinical state (if yes, *manual entry*) | Presence of CPP crystals in SFA without clinical disease element  CR evidence of CPP crystal deposition without clinical disease element  US evidence of CPP crystal deposition without clinical disease element  CT evidence of CPP crystal deposition without clinical disease element  DECT evidence of CPP crystal deposition without clinical disease element  MRI evidence of CPP crystal deposition without clinical disease element  Other |
|  | Disease course state (if yes, *manual entry*) | One episode of acute calcium pyrophosphate crystal arthritis  More than one episode of acute calcium pyrophosphate crystal arthritis with asymptomatic period between the acute episodes  Persistent inflammatory arthritis due to calcium pyrophosphate crystal deposition  Osteoarthritis with evidence of calcium pyrophosphate deposition  Periarticular involvement due to calcium pyrophosphate crystal deposition  Nerves compression secondary to calcium pyrophosphate crystal deposits  Spinal involvement due to calcium pyrophosphate crystal deposition  Other |
