# Supplementary material for: Systematic literature review on Calcium Pyrophosphate Deposition (CPPD) nomenclature: condition elements and clinical states— A Gout, Hyperuricaemia and Crystal-Associated Disease Network (G-CAN) consensus project
Source: RMD Open. 2025 Jan 30;11(1):e004847. doi: 10.1136/rmdopen-2024-004847 (PMC11784236; doi:10.1136/rmdopen-2024-004847)
Supplement: online supplemental table 3 [file rmdopen-11-1-s003.docx]

**Supplementary Table S3. Included articles**

|  | **Included articles** | **Publi. year** | **Study design** | **Reference standard** | **CPPD designation^a^** | **CPPD definition in article** |
| --- | --- | --- | --- | --- | --- | --- |
| 1 | Kakitsubata et al. ^1^ | 2000 | Case series | Imaging | Disease | - |
| 2 | Agudelo et al. ^2^ | 2000 | Scoping review | McCarty criteria | Disease | “usually presents as acute, self-limited episodes of monoarthritis, but may be associated with polyarticular attacks and chronic arthritis”; “is associated more often with onset in elderly patients and has more predictable features in this population” |
| 3 | Aoyama et al. ^3^ | 2000 | Case report | Histology | Disease | - |
| 4 | Baysal et al. ^4^ | 2000 | Case report | Imaging | Disease | “referred to as articular chondrocalcinosis or pseudogout”; “is considered to be one of the most common forms of crystal-induced arthritis” |
| 5 | Calò et al. ^5^ | 2000 | Scoping review | SFA | - | - |
| 6 | Caramaschi et al. ^6^ | 2000 | Letter to the editor | SFA | - | - |
| 7 | McGill et al. ^7^ | 2000 | Scoping review | SFA | - | - |
| 8 | Cheung et al. ^8^ | 2000 | Scoping review | - | - | “a group of clinically heterogeneous arthritides, of unknown etiology, that are a significant source of morbidity in the elderly” |
| 9 | Cibere et al. ^9^ | 2000 | Case report | SFA | Disease | - |
| 10 | Lomoschitz et al. ^10^ | 2000 | Case report | Histology | Deposition | “is characterized by acute, subacute or chronic joint inflammation caused by deposition of CPPD crystals in degenerated hyalin cartilage, fibrocartilage and synovial structures, tendons, ligaments and other extraarticular soft tissues” |
| 11 | Kroesen et al. ^11^ | 2000 | Case report | SFA | Disease | - |
| 12 | Li-Yu et al. ^12^ | 2000 | Case report | Histology | Deposition | “can vary from asymptomatic crystal deposits in an articular fibrocartilage, acute arthritis (pseudogout), or a severe form of destructive arthropathy” |
| 13 | Mostafapour et al. ^13^ | 2000 | Case series | Histology | - | - |
| 14 | Oostveen et al. ^14^ | 2000 | Systematic literature review | Imaging | - | “is characterized by the presence of calcification of the articular cartilage, menisci, synovium, and periarticular tissues”; “can cause destructive lesions of vertebral bodies and disc spaces and calciﬁcation of discs” |
| 15 | Hasegawa et al. ^15^ | 2000 | Case report | Histology | - | - |
| 16 | Punzi et al. ^16^ | 2000 | Letter to the editor | - | Deposition | - |
| 17 | Steinbach et al. ^17^ | 2000 | Scoping review | - | - | - |
| 18 | Sagarin et al. ^18^ | 2000 | Case report | SFA | Disease | “is a disease of the elderly”; “mild chronic symptoms punctuated by occasional acute exacerbations” |
| 19 | Tedd et al. ^19^ | 2000 | Case report | SFA | Disease | - |
| 20 | Teramoto et al. ^20^ | 2000 | Letter to the editor | - | - | - |
| 21 | Usuki et al. ^21^ | 2000 | Case report | SFA | - | - |
| 22 | Yamagami et al. ^22^ | 2000 | Case report | Imaging | Deposition | - |
| 23 | Wong et al. ^23^ | 2000 | Case series | SFA | Deposition | - |
| 24 | Malaviya et al. ^24^ | 2001 | Cohort study longitudinal | McCarty criteria | Deposition | - |
| 25 | Aoyama et al. ^25^ | 2001 | Case report | Imaging | Deposition | - |
| 26 | Assaker et al. ^26^ | 2001 | Case series | Imaging | Deposition | “a well-known rheumatological entity and a common form of crystal deposition arthritis” |
| 27 | Bernardeau et al. ^27^ | 2001 | Case series | - | Deposition | - |
| 28 | Mula et al. ^28^ | 2001 | Case report | Imaging | Deposition | - |
| 29 | Cabre et al. ^29^ | 2001 | Cohort study retrospective | Histology | - | - |
| 30 | Maldonado et al. ^30^ | 2001 | Scoping review | - | Deposition | - |
| 31 | Canhão et al. ^31^ | 2001 | Cohort study cross-sectional | SFA & Imaging | Deposition | “a well-known but heterogeneous disease with a variable presentation and course” |
| 32 | Caspi et al. ^32^ | 2001 | Cohort study cross-sectional | Imaging | - | - |
| 33 | Kelley et al. ^33^ | 2001 | Case series | SFA | Deposition | “is either an incidental finding, or patients may present with an acute mono or polyarthritis or with a chronic arthropathy associated with osteoarthritis” |
| 34 | Chivukula et al. ^34^ | 2001 | Case report | Histology | Disease | “acute or chronic inflammation with joint pain mimicking gout” |
| 35 | Coombs et al. ^35^ | 2001 | Case report | Histology | Deposition | “has multiple clinical manifestations. These range from asymptomatic to a painful symptomatic arthropathy”; “is characterized by the intra-articular presence of calcium pyrophosphate dihydrate crystals” |
| 36 | Olin et al. ^36^ | 2001 | Case report | Histology | Deposition | “is characterized by absence of symptoms or severely destructive arthropathy” |
| 37 | Lambrecht et al. ^37^ | 2001 | Case report | Histology | Disease | - |
| 38 | Matsukado et al. ^38^ | 2001 | Case report | Histology | Deposition | - |
| 39 | Ohira et al. ^39^ | 2001 | Cohort study retrospective | Histology | Deposition | - |
| 40 | Halverson et al. ^40^ | 2001 | Scoping review | - | - | - |
| 41 | Eriksson et al. ^41^ | 2001 | Case report | SFA & Histology | - | - |
| 42 | Shidham et al. ^42^ | 2001 | Cohort study longitudinal | Histology | Disease | - |
| 43 | Perez-Ruiz et al. ^43^ | 2001 | Case report | SFA | - | - |
| 44 | Peter et al. ^44^ | 2001 | Cohort study longitudinal | Imaging & Expert opinion | - | - |
| 45 | Selvi et al. ^45^ | 2001 | Case-control study prospective | SFA & Imaging | Disease | - |
| 46 | Reuge et al. ^46^ | 2001 | Cohort study retrospective | Imaging | Deposition | - |
| 47 | Rosenthal et al. ^47^ | 2001 | Systematic literature review | SFA | - | - |
| 48 | Yamakawa et al. ^48^ | 2001 | Cohort study retrospective | Imaging & Histology | Deposition | - |
| 49 | Van den Bosch et al. ^49^ | 2001 | Case series | Histology | - | - |
| 50 | Wendling et al. ^50^ | 2001 | Case report | Imaging | - | - |
| 51 | Jaovisidha et al. ^51^ | 2002 | Scoping review | - | - | - |
| 52 | Al-Arfaj et al. ^52^ | 2002 | Cohort study cross-sectional | - | Deposition | - |
| 53 | Al-Arfaj et al. ^53^ | 2002 | Cohort study cross-sectional | - | Deposition | - |
| 54 | Biankin et al. ^54^ | 2002 | Case report | Histology | Deposition | “may rarely be an inherited metabolic disorder but is most commonly either associated with previous joint damage or with another disorder”; “has various presentations and is most commonly asymptomatic” |
| 55 | Song et al. ^55^ | 2002 | Case report | SFA | Deposition | “includes arthritic syndromes associated with calcium pyrophosphate dihydrate crystal deposition in articular tissues”; “has three common presentations consisting of acute synovitis that was also called pseudogout clinically, chronic arthritis, and asymptomatic chondrocalcinosis” |
| 56 | Ziswiler et al. ^56^ | 2002 | Case report | SFA | - | - |
| 57 | Cottrell et al. ^57^ | 2002 | Case report | Histology | Disease | “is characterized by the presence of CPPD crystals in the joint space” |
| 58 | Kobayashi et al. ^58^ | 2002 | Case series | SFA & Histology | Deposition | - |
| 59 | Derfus et al. ^59^ | 2002 | Cohort study longitudinal | - | - | - |
| 60 | Pakzad et al. ^60^ | 2002 | Case report | Histology | Deposition | “a relatively rare disease with variable clinical presentations”; “a relatively uncommon disease that usually involves the elderly population” |
| 61 | Sofka et al. ^61^ | 2002 | Case report | Histology | Deposition | “is characterized radiographically by calcification in and around joints, usually affecting the non-weight-bearing surfaces” |
| 62 | Houdaille et al. ^62^ | 2002 | Case report | SFA | - | - |
| 63 | Hayashi et al. ^63^ | 2002 | Case report | SFA | - | “a common acute or chronic articular disorder among the elderly” |
| 64 | Greaves et al. ^64^ | 2002 | Case report | SFA | - | “a rare and benign condition in which CPPD crystals are deposited in synovial fluid and which results in the calcification of articular cartilage”; “acute presentation mimics gout, chronic features resemble osteoarthritis” |
| 65 | Guggi et al. ^65^ | 2002 | Cohort study cross-sectional | SFA | - | - |
| 66 | Gadgil et al. ^66^ | 2002 | Case report | - | - | “a disorder of pyrophosphate metabolism that occurs in older patients” |
| 67 | Gálvez et al. ^67^ | 2002 | Cohort study cross-sectional | SFA | - | - |
| 68 | Foldes et al. ^68^ | 2002 | Case-control study prospective | Imaging | - | - |
| 69 | Dodd et al. ^69^ | 2002 | Scoping review | - | - | - |
| 70 | Santos-Ocampo et al. ^70^ | 2002 | Case report | SFA | Disease | - |
| 71 | Swan et al. ^71^ | 2002 | Systematic literature review | SFA | - | - |
| 72 | Romera et al. ^72^ | 2002 | Letter to the editor | - | Deposition | - |
| 73 | Terkeltaub et al. ^73^ | 2002 | Scoping review | - | Deposition | - |
| 74 | Terkeltaub et al. ^74^ | 2002 | Case series | SFA | Deposition | - |
| 75 | Rubin et al. ^75^ | 2002 | Scoping review | SFA | Disease | “occurs when CPPD crystals are identified in joint fluid”; “in which most patients are asymptomatic, but acute crystal synovitis or chronic arthropathy can be present” |
| 76 | Waguri-Nagaya et al. ^76^ | 2002 | Case report | Imaging | Deposition | - |
| 77 | Jackson et al. ^77^ | 2003 | Systematic literature review | - | - | - |
| 78 | Ahn et al. ^78^ | 2003 | Case report | Imaging | Disease | “a disease of the elderly and extremely rare in young individuals”; “may be an incidental finding, or may be associated with severe clinical symptoms including arthritis” |
| 79 | Baty et al. ^79^ | 2003 | Case report | Imaging | Deposition | “usually occur in elderly patients, and range from incidental findings to a destructive arthropathy” |
| 80 | Bencardino et al. ^80^ | 2003 | Scoping review | - | Deposition | “is characterized by the accumulation of pyrophosphate dihydrate crystals in articular and periarticular tissue”; “is among many conditions that may result in crystal deposition within cartilage” |
| 81 | Brandt et al. ^81^ | 2003 | Scoping review | - | - | - |
| 82 | Cañete et al. ^82^ | 2003 | Cohort study cross-sectional | SFA & Imaging | Disease | - |
| 83 | Caramaschi et al. ^83^ | 2003 | Case report | Histology | Disease | - |
| 84 | Neame et al. ^84^ | 2003 | Cohort study retrospective | Imaging | Deposition | “may be sporadic, familial or secondary to a variety of metabolic abnormalities” |
| 85 | Nalbant et al. ^85^ | 2003 | Cohort study retrospective | SFA | Deposition | - |
| 86 | Muthukumar et al. ^86^ | 2003 | Cohort study longitudinal | Histology | Deposition | - |
| 87 | Ofluoğlu et al. ^87^ | 2003 | Case report | Imaging | Deposition | - |
| 88 | Osano et al. ^88^ | 2003 | Case report | Histology | Deposition | “a common age-related phenomenon” |
| 89 | Ozolek et al. ^89^ | 2003 | Case report | Histology | Deposition | “an increasingly common form of arthritis in the elderly affecting articular and periarticular soft tissues” |
| 90 | Sofka et al. ^90^ | 2003 | Scoping review | Imaging | Deposition | “a specific metabolic condition characterized by CPPD crystal deposition in and around joints” |
| 91 | Havitçioğlu et al. ^91^ | 2003 | Case report | - | - | “is an inflammatory pyrophosphate arthropathy especially affecting the middle-aged and elderly population”; “has multiple clinical features with variable courses creating several pitfalls in clinical diagnosis” |
| 92 | Hamburger et al. ^92^ | 2003 | Scoping review | - | - | - |
| 93 | Halverson et al. ^93^ | 2003 | Scoping review | - | - | - |
| 94 | Pay et al. ^94^ | 2003 | Systematic literature review | Expert opinion | Deposition | - |
| 95 | Sallis et al. ^95^ | 2003 | Scoping review | - | - | - |
| 96 | Rutsch et al. ^96^ | 2003 | Scoping review | - | Deposition | - |
| 97 | Yuan et al. ^97^ | 2003 | Letter to the editor | - | Deposition | - |
| 98 | Williams et al. ^98^ | 2003 | Systematic literature review | - | Deposition | - |
| 99 | McKee et al. ^99^ | 2004 | Case report | - | Deposition | “is a relatively common form of arthropathy characterized by the abnormal deposition of calcium pyrophosphate crystals in articular cartilage” |
| 100 | Abreu et al. ^100^ | 2004 | Case series | Imaging & Histology | Disease | “is the most common crystalline arthropathy, characterized by acute, subacute or chronic joint symptoms and signs, and deposition of CPPD crystals in hyaline cartilage fibrocartilage and other soft tissue structures” |
| 101 | Aouba et al. ^101^ | 2004 | Case series | Imaging | - | - |
| 102 | Mader et al. ^102^ | 2004 | Case report | SFA | Deposition | - |
| 103 | Béjia et al. ^103^ | 2004 | Cohort study cross-sectional | Imaging & Histology | Deposition | “is characterized by the deposition of calcium containing crystals within joint cartilage and fibrocartilage” |
| 104 | Boutboul et al. ^104^ | 2004 | Case report | - | - | - |
| 105 | Nordström et al. ^105^ | 2004 | Case report | - | Deposition | - |
| 106 | Chen et al. ^106^ | 2004 | Scoping review | SFA | Deposition | - |
| 107 | Marsot-Dupuch et al. ^107^ | 2004 | Case series | SFA & Histology | Deposition | “is a metabolic disease associated with periarticular and intra-articular calcification”; “is an uncommon disorder that primarily affects patients older than 50 years”; “is a crystal deposition disease similar to gout” |
| 108 | Netter et al. ^108^ | 2004 | Scoping review | - | Deposition | “is a chronic condition in which calcium pyrophosphate dihydrate deposition microcrystals deposit in the joint fluid cartilage and periarticular tissues” |
| 109 | Griesdale et al. ^109^ | 2004 | Case report | Histology | - | “is an inflammatory arthropathy characterized by the deposition of [...] crystals in articular and periarticular structures” |
| 110 | Goldblatt et al. ^110^ | 2004 | Case report | SFA | Disease | “is a heterogeneous disorder, with clinical presentations ranging from an acute arthritis to chronic pain secondary to degenerative cartilage disease” |
| 111 | Falsetti et al. ^111^ | 2004 | Case-control study prospective | McCarty criteria | - | “is an arthropathy generally caused by deposit of calcium pyrophosphate-dihydrate microcrystals in the joints and in the tendons” |
| 112 | Ea et al. ^112^ | 2004 | Scoping review | - | - | - |
| 113 | Pasquetti et al. ^113^ | 2004 | Case report | SFA | Deposition | - |
| 114 | Steinbach et al. ^114^ | 2004 | Scoping review | - | - | - |
| 115 | Saffar et al. ^115^ | 2004 | Scoping review | SFA & Imaging & Histology | Deposition | - |
| 116 | Sato et al. ^116^ | 2004 | Case report | SFA & Imaging | Disease | - |
| 117 | Zhang et al. ^117^ | 2004 | Cohort study longitudinal | Imaging | Deposition | - |
| 118 | Meul et al. ^118^ | 2005 | Case report | Histology & Expert opinion | Deposition | “a rare joint disease which is characterized by the presence of calcium pyrophosphate dihydrate crystals in the intra-articular and periarticular tissue” |
| 119 | Mahmud et al. ^119^ | 2005 | Case series | Histology | Deposition | “is characterized by acute, subacute, or chronic inflammation joints” |
| 120 | McCarthy et al. ^120^ | 2005 | Scoping review | - | Deposition | - |
| 121 | Campo-Ruiz et al. ^121^ | 2005 | Cohort study cross-sectional | Histology | - | - |
| 122 | Srinivasan et al. ^122^ | 2005 | Case report | Histology | Deposition | “is a disorder characterized by the presence of CPPD crystals in or around joints” |
| 123 | Choy et al. ^123^ | 2005 | Scoping review | SFA & Histology | Deposition | “is characterized by the deposition of calcium pyrophosphate crystals in hyaline cartilage fibrocartilage and other soft tissue structures” |
| 124 | Dalbeth et al. ^124^ | 2005 | Scoping review | - | Deposition | - |
| 125 | Paolini et al. ^125^ | 2005 | Case series | Histology | Deposition | “is a well-recognized systemic arthropathy” |
| 126 | Papakonstantinou et al. ^126^ | 2005 | Case report | - | - | - |
| 127 | Smolka et al. ^127^ | 2005 | Case report | Histology | - | “is characterized by a crystal-induced synovitis caused by the accumulation of pyrophosphate dihydrate crystals in articular and periarticular tissues” |
| 128 | Holt et al. ^128^ | 2005 | Case report | - | - | - |
| 129 | Gupta et al. ^129^ | 2005 | Case report | Expert opinion | - | - |
| 130 | Frediani et al. ^130^ | 2005 | Case-control study prospective | SFA | Deposition | - |
| 131 | Ea et al. ^131^ | 2005 | Case report | Imaging | - | - |
| 132 | Pascual et al. ^132^ | 2005 | Scoping review | SFA | - | - |
| 133 | Shih et al. ^133^ | 2005 | Scoping review | SFA | Deposition | - |
| 134 | Pego-Reigosa et al. ^134^ | 2005 | Cohort study longitudinal | - | - | - |
| 135 | Shah et al. ^135^ | 2005 | Cohort study longitudinal | - | - | - |
| 136 | Suan et al. ^136^ | 2005 | Case series | - | Deposition | - |
| 137 | Swayamprakasam et al. ^137^ | 2005 | Case report | SFA | Disease | “a disease similar to gout but which is characterized by the deposition of calcium pyrophosphate dihydrate crystals within joints” |
| 138 | Richette et al. ^138^ | 2005 | Case report | Imaging | Deposition | - |
| 139 | Sato et al. ^139^ | 2005 | Case report | Imaging | Deposition | - |
| 140 | Tshering et al. ^140^ | 2005 | Case series | Imaging | - | - |
| 141 | Young-Min et al. ^141^ | 2005 | Case report | SFA | Deposition | - |
| 142 | Wu et al. ^142^ | 2005 | Case series | Imaging | Deposition | - |
| 143 | Wise et al. ^143^ | 2005 | Systematic literature review | Imaging & Histology | Deposition | - |
| 144 | Molloy et al. ^144^ | 2006 | Scoping review | - | - | - |
| 145 | Atzeni et al. ^145^ | 2006 | Scoping review | - | Deposition | “a metabolic arthropathy caused by calcium pyrophosphate crystal deposits”; “is frequent in the second half of life” |
| 146 | Lin et al. ^146^ | 2006 | Case report | Histology | Deposition | - |
| 147 | Cascone et al. ^147^ | 2006 | Case report | Histology | - | - |
| 148 | Choi et al. ^148^ | 2006 | Scoping review | McCarty criteria | Deposition | “is characterized by the presence of CPPD crystals within and around joints” |
| 149 | Cohen et al. ^149^ | 2006 | Scoping review | - | Deposition | - |
| 150 | Rosenthal et al. ^150^ | 2006 | Scoping review | - | Deposition | - |
| 151 | Neogi et al. ^151^ | 2006 | Cohort study longitudinal | - | - | - |
| 152 | Devauchelle-Pensec et al. ^152^ | 2006 | Cohort study longitudinal | Imaging | Disease | - |
| 153 | De Filippo et al. ^153^ | 2006 | Scoping review | - | - | - |
| 154 | Harrington et al. ^154^ | 2006 | Scoping review | - | - | - |
| 155 | Gerster et al. ^155^ | 2006 | Cohort study retrospective | SFA | - | - |
| 156 | Grassi et al. ^156^ | 2006 | Scoping review | - | - | - |
| 157 | Feydy et al. ^157^ | 2006 | Scoping review | - | - | “is among many conditions that may result in crystal deposition within cartilage” |
| 158 | Ellman et al. ^158^ | 2006 | Scoping review | - | - | - |
| 159 | Pytel et al. ^159^ | 2006 | Cohort study retrospective | Histology | Deposition | - |
| 160 | Rajakulendran et al. ^160^ | 2006 | Case report | Imaging | - | - |
| 161 | Taggarshe et al. ^161^ | 2006 | Case report | McCarty criteria | - | - |
| 162 | Zhang et al. ^162^ | 2006 | Cohort study longitudinal | Imaging | - | - |
| 163 | Watanabe et al. ^163^ | 2006 | Case report | SFA | Disease | - |
| 164 | Josefina et al. ^164^ | 2007 | Case report | SFA | Disease | - |
| 165 | Mitsuyama et al. ^165^ | 2007 | Cohort study cross-sectional | Histology | Deposition | “can cause acute attacks of inflammatory arthritis, such as pseudogout, erosive arthritis, or periarthritis, and are associated with an exaggerated form of osteoarthritis” |
| 166 | Ames et al. ^166^ | 2007 | Case report | - | Disease | - |
| 167 | Ariyoshi et al. ^167^ | 2007 | Case report | Histology | - | “a well-recognized inflammatory joint disorder” |
| 168 | Lam et al. ^168^ | 2007 | Case series | SFA | Deposition | “the deposition of CPPD crystals in the articular or periarticular structures leading to acute, subacute or chronic inflammation of the joints” |
| 169 | Cameron et al. ^169^ | 2007 | Case report | SFA & Imaging | - | “is characterised by the clinical features of arthritis, radiographic chondrocalcinosis in both hyaline and fibrocartilage, and identification of calcium pyrophosphate crystals in either synovial fluid or excised tissue” |
| 170 | Sonsale et al. ^170^ | 2007 | Case report | SFA | Disease | - |
| 171 | Chollet-Janin et al. ^171^ | 2007 | Cohort study retrospective | - | Deposition | “a common and potentially severe metabolic arthropathy caused by calcium pyrophosphate dihydrate crystal deposition”; “can cause severe acute attacks of inflammatory arthritis, mainly in the knees and wrists, as well as various forms of chronic, frequently destructive, arthropathies” |
| 172 | Chong et al. ^172^ | 2007 | Cohort study retrospective | SFA | - | - |
| 173 | Nicholas et al. ^173^ | 2007 | Case report | Imaging | - | “a relatively rare condition usually found in the large joints of the knee, wrist, and shoulder”; “a rare erosive metabolic arthropathy characterized by intra- and periarticular calcium crystals”; “a disorder of late middle age and the elderly” |
| 174 | De Geeter et al. ^174^ | 2007 | Case report | - | - | - |
| 175 | Delle et al. ^175^ | 2007 | Scoping review | - | Disease | - |
| 176 | Carlson et al. ^176^ | 2007 | Scoping review | Histology | - | - |
| 177 | Hirose et al. ^177^ | 2007 | Case report | - | - | “a disease of acute or chronic inflammatory synovitis characterized by calcium pyrophosphate dihydrate crystals within a joint” |
| 178 | Giulioni et al. ^178^ | 2007 | Case report | Histology | - | - |
| 179 | Goto et al. ^179^ | 2007 | Cohort study retrospective | Imaging | - | - |
| 180 | Filippou et al. ^180^ | 2007 | Case-control study prospective | SFA | Deposition | - |
| 181 | Epis et al. ^181^ | 2007 | Cohort study cross-sectional | SFA | - | - |
| 182 | Doumas et al. ^182^ | 2007 | Scoping review | - | - | - |
| 183 | Doita et al. ^183^ | 2007 | Case report | Histology | - | “is not a rare condition among the elderly” |
| 184 | Peach et al. ^184^ | 2007 | Systematic literature review | - | Deposition | - |
| 185 | Shah et al. ^185^ | 2007 | Cohort study retrospective | SFA | Disease | - |
| 186 | Sethi et al. ^186^ | 2007 | Case report | Histology | Disease | “is a form of arthritis in elderly patients that affects articular and periarticular soft tissues”; “can also occur in spinal structures, such as intervertebral disks, ligaments, bursae, articular cartilage, synovium, and joint capsules” |
| 187 | Scutellari et al. ^187^ | 2007 | Case-control study prospective | Imaging | Deposition | “is the most common crystal arthropathy”; “refers to a disorder characterized by the exclusive presence of CPPD crystals in or around the joints”; “ranges from an incidental radiographic finding to destructive arthropathy” |
| 188 | Richette et al. ^188^ | 2007 | Cohort study cross-sectional | - | Deposition | - |
| 189 | Scavarda et al. ^189^ | 2007 | Case report | Imaging | Disease | - |
| 190 | Rothschild et al. ^190^ | 2007 | Case report | - | Disease | - |
| 191 | Rosenthal et al. ^191^ | 2007 | Scoping review | SFA | - | - |
| 192 | Viriyavejkul et al. ^192^ | 2007 | Cohort study cross-sectional | - | Deposition | - |
| 193 | Volpe et al. ^193^ | 2007 | Case series | - | - | - |
| 194 | Wakefield et al. ^194^ | 2007 | Letter to the editor | - | - | - |
| 195 | Jennings et al. ^195^ | 2008 | Scoping review | - | - | “associated with deposition of calcium pyrophosphate dehydrate crystals and characterized by joint effusions with marked neutrophilia and a form of secondary osteoarthritis (OA) with a pattern of joint involvement that differs from primary osteoarthritis” |
| 196 | Albert et al. ^196^ | 2008 | Cohort study cross-sectional | Histology | Disease | - |
| 197 | Announ et al. ^197^ | 2008 | Scoping review | SFA | Disease | “a common and potentially severe metabolic arthropathy” |
| 198 | Naqvi et al. ^198^ | 2008 | Case report | Histology | Deposition | “is characterized by the accumulation of calcium pyrophosphate dihydrate crystals in intra-articular and periarticular tissues” |
| 199 | Ascani et al. ^199^ | 2008 | Case report | Histology | - | “a juxta-articular disease resulting from deposition of calcium pyrophosphate” |
| 200 | Martens et al. ^200^ | 2008 | Case report | Histology | Deposition | - |
| 201 | Brunot et al. ^201^ | 2008 | Letter to the editor | Imaging | - | “a microcrystalline disease characterized radiographically by multiple foci of calcification in hyaline and fibrocartilage of the joints and intervertebral discs” |
| 202 | O'Connor et al. ^202^ | 2008 | Case report | SFA | Disease | - |
| 203 | Ding et al. ^203^ | 2008 | Case report | SFA | - | - |
| 204 | McGonagle et al. ^204^ | 2008 | Case report | SFA | Deposition | - |
| 205 | Nuki et al. ^205^ | 2008 | Scoping review | - | Deposition | - |
| 206 | Mikami et al. ^206^ | 2008 | Case report | Histology | Deposition | “a type of crystal arthritis and occurs as a result of deposition of CPPD in the synovial membrane or cartilage of the joint” |
| 207 | Das et al. ^207^ | 2008 | Scoping review | - | - | - |
| 208 | Del Rincón et al. ^208^ | 2008 | Case report | Histology | - | - |
| 209 | Checa et al. ^209^ | 2008 | Case report | Histology | - | - |
| 210 | Sivera et al. ^210^ | 2008 | Cohort study cross-sectional | SFA | - | - |
| 211 | Pawlotsky et al. ^211^ | 2008 | Case-control study retrospective | - | Deposition | - |
| 212 | Pritzker et al. ^212^ | 2008 | Letter to the editor | - | Deposition | - |
| 213 | Sander et al. ^213^ | 2008 | Case report | Histology | Disease | - |
| 214 | Queiro et al. ^214^ | 2008 | Case series | SFA | Deposition | - |
| 215 | Su et al. ^215^ | 2008 | Case report | - | Disease | - |
| 216 | Tan et al. ^216^ | 2008 | Letter to the editor | Histology | Disease | - |
| 217 | Rood et al. ^217^ | 2008 | Scoping review | - | Deposition | - |
| 218 | Sarraf et al. ^218^ | 2008 | Scoping review | - | Deposition | - |
| 219 | Yamazaki et al. ^219^ | 2008 | Case report | SFA | Deposition | “an inflammatory pyrophosphate arthropathy that generally occurs in the middle-aged and elderly population” |
| 220 | Valenti et al. ^220^ | 2008 | Cohort study cross-sectional | Imaging | - | - |
| 221 | Yabuki et al. ^221^ | 2008 | Case series | Imaging | Deposition | - |
| 222 | Whelan et al. ^222^ | 2008 | Case report | - | - | - |
| 223 | Wendling et al. ^223^ | 2008 | Case report | SFA | Deposition | - |
| 224 | Kalish et al. ^224^ | 2009 | Case report | Histology | Deposition | - |
| 225 | Nachimuthu et al. ^225^ | 2009 | Case report | - | Deposition | - |
| 226 | Announ et al. ^226^ | 2009 | Case report | SFA | Disease | - |
| 227 | Armas et al. ^227^ | 2009 | Scoping review | SFA | Deposition | “is a disease of older patients which prevalence increases with the increasing of age” |
| 228 | Beck et al. ^228^ | 2009 | Scoping review | - | Deposition | - |
| 229 | Bullocks et al. ^229^ | 2009 | Case series | SFA & Histology | - | “occurs as a result of changes in concentrations of pyrophosphate and phosphate” |
| 230 | Chen et al. ^230^ | 2009 | Case report | Imaging | Disease | “is the accumulation of CPPD in human tissues such as cartilage, synovium, tendons or ligaments” |
| 231 | Ciapetti et al. ^231^ | 2009 | Cohort study cross-sectional | SFA | Disease | “is characterized by acute or chronic inflammation due to deposit of calcium pyrophosphate dihydrate crystals in articular cartilage and periarticular soft tissues” |
| 232 | Richette et al. ^232^ | 2009 | Scoping review | SFA & Histology | Deposition | “the most common cause of radiographic articular calcification at the knee”; “term used to incorporate various phenotypes” |
| 233 | Pascual et al. ^233^ | 2009 | Scoping review | SFA | Deposition | - |
| 234 | Courtney et al. ^234^ | 2009 | Scoping review | SFA | - | - |
| 235 | Covani et al. ^235^ | 2009 | Case report | Histology | Disease | “is an uncommon disorder that primarily affects patients older than 50 years”; “a crystal-induced synovitis caused by the accumulation of pyrophosphate dihydrate crystals in articular and peri-articular tissues” |
| 236 | Dalbeth et al. ^236^ | 2009 | Scoping review | Imaging | Deposition | - |
| 237 | Rosenthal et al. ^237^ | 2009 | Systematic literature review | - | Deposition | - |
| 238 | Mehta et al. ^238^ | 2009 | Case report | SFA | Disease | - |
| 239 | Siva et al. ^239^ | 2009 | Scoping review | SFA | Disease | - |
| 240 | Hamilton et al. ^240^ | 2009 | Case report | Histology | - | “usually manifests as a polyarticular arthritis, although it can initially present as a monoarticular disorder affecting the knees, shoulders, wrists and fingers” |
| 241 | Galed-Placed et al. ^241^ | 2009 | Case report | SFA | - | “encompasses pseudogout, articular chondrocalcinosis and all of the other inflammatory and degenerative joint lesions of this disease” |
| 242 | Fuerst et al. ^242^ | 2009 | Case-control study prospective | Histology | - | - |
| 243 | Fuerst et al. ^243^ | 2009 | Cohort study cross-sectional | - | - | - |
| 244 | Frey et al. ^244^ | 2009 | Case report | Imaging | - | “an inflammatory condition that has been associated with several reported patterns of arthropathy” |
| 245 | Filippucci et al. ^245^ | 2009 | Case-control study prospective | McCarty criteria | - | - |
| 246 | Ellman et al. ^246^ | 2009 | Case report | - | - | - |
| 247 | Ea et al. ^247^ | 2009 | Scoping review | - | - | - |
| 248 | Pritzker et al. ^248^ | 2009 | Scoping review | - | Deposition | - |
| 249 | Schlesinger et al. ^249^ | 2009 | Cohort study retrospective | - | - | - |
| 250 | Unlu et al. ^250^ | 2009 | Case report | SFA & Imaging | - | - |
| 251 | Yanai et al. ^251^ | 2009 | Case series | Imaging | Disease | - |
| 252 | Volpe et al. ^252^ | 2009 | Case report | McCarty criteria | Deposition | - |
| 253 | Wener et al. ^253^ | 2009 | Case report | Histology | Deposition | - |
| 254 | Maggio et al. ^254^ | 2010 | Case report | SFA | Deposition | - |
| 255 | Ishikawa et al. ^255^ | 2010 | Case report | Imaging | - | - |
| 256 | Brent et al. ^256^ | 2010 | Case report | Histology | Deposition | - |
| 257 | Carda et al. ^257^ | 2010 | Case report | SFA | Disease | - |
| 258 | Hsu et al. ^258^ | 2010 | Case report | Histology | Disease | “a variety of metabolic arthropathy caused by the deposition of calcium pyrophosphate dihydrate crystals in and around joints” |
| 259 | Córdoba-Fernández et al. ^259^ | 2010 | Case report | SFA & Histology | Deposition | - |
| 260 | Cranenburg et al. ^260^ | 2010 | Cohort study cross-sectional | - | - | - |
| 261 | Dala-Ali et al. ^261^ | 2010 | Case report | SFA | Disease | - |
| 262 | Dallos et al. ^262^ | 2010 | Cohort study cross-sectional | Imaging | - | - |
| 263 | Kathju et al. ^263^ | 2010 | Case report | Histology | - | - |
| 264 | Kato et al. ^264^ | 2010 | Case report | Histology | Deposition | “is caused by deposition of calcium pyrophosphate dihydrate crystals in the articular cartilage or synovium” |
| 265 | Nowatzky et al. ^265^ | 2010 | Scoping review | - | Deposition | - |
| 266 | Kishore et al. ^266^ | 2010 | Case report | Histology | Deposition | “a rare benign crystalline arthopathy of unknown cause”; “a well-recognized inflammatory joint disorder characterized by presence of calcium pyrophosphate dihydrate crystals in intraarticular and periarticular tissue” |
| 267 | Omura et al. ^267^ | 2010 | Systematic literature review | SFA | - | - |
| 268 | Gutierrez et al. ^268^ | 2010 | Case series | SFA | - | - |
| 269 | Gutierrez et al. ^269^ | 2010 | Case report | SFA | - | “is characterized by deposits of CPPD crystals within the hyaline or fibrocartilage of the joints or in the periarticular soft tissues” |
| 270 | Fuerst et al. ^270^ | 2010 | Case-control study prospective | Histology | - | - |
| 271 | Filippucci et al. ^271^ | 2010 | Case-control study prospective | - | - | - |
| 272 | Emary et al. ^272^ | 2010 | Case report | - | - | - |
| 273 | Dufauret-Lombard et al. ^273^ | 2010 | Scoping review | - | - | “is a common crystal deposition joint disease in which calcium pyrophosphate dihydrate crystals deposit within joint cartilage and fibrocartilage” |
| 274 | Sekijima et al. ^274^ | 2010 | Cohort study retrospective | Imaging | - | “is one of the most common forms of crystal-associated arthropathy in the elderly” |
| 275 | Pollock et al. ^275^ | 2010 | Case-control study longitudinal | Imaging | Deposition | - |
| 276 | Sahinbegovic et al. ^276^ | 2010 | Cohort study cross-sectional | Imaging | - | - |
| 277 | Tamborrini et al. ^277^ | 2010 | Case report | SFA | Disease | - |
| 278 | Taniguchi et al. ^278^ | 2010 | Case series | Imaging | Deposition | - |
| 279 | Zadaka et al. ^279^ | 2010 | Case series | SFA | Deposition | - |
| 280 | Viana et al. ^280^ | 2010 | Scoping review | Imaging | - | - |
| 281 | Wen et al. ^281^ | 2010 | Systematic literature review | Imaging | Deposition | - |
| 282 | Meng et al. ^282^ | 2011 | Case report | Histology | Deposition | - |
| 283 | Jansen et al. ^283^ | 2011 | Scoping review | - | - | “is due to crystals deposited in fibro- and hyaline cartilage and often seen in degenerative joint disease, especially in the knee” |
| 284 | Mebarek et al. ^284^ | 2011 | Scoping review | - | Deposition | - |
| 285 | Abhishek et al. ^285^ | 2011 | Scoping review | - | - | “is the most common cause of the calcification of articular fibrocartilage or hyaline cartilage” |
| 286 | Abhishek et al. ^286^ | 2011 | Case-control study retrospective | Imaging | Deposition | “may be an asymptomatic incidental finding on imaging or may be associated with acute CCP crystal arthritis osteoarthritis (OA) and/or chronic CCP crystal inflammatory arthritis” |
| 287 | Ali et al. ^287^ | 2011 | Case report | Histology | Deposition | “a metabolic arthropathy caused by the deposition of calcium pyrophosphate dihydrate in and around joints especially in articular and fibrocartilage” |
| 288 | Arauz-Rivera et al. ^288^ | 2011 | Case report | Imaging | - | - |
| 289 | Aran et al. ^289^ | 2011 | Randomized controlled trial | - | - | - |
| 290 | Checa et al. ^290^ | 2011 | Case report | SFA & Histology | Deposition | - |
| 291 | Siau et al. ^291^ | 2011 | Case series | Imaging | Deposition | - |
| 292 | Lahmer et al. ^292^ | 2011 | Case report | SFA & Imaging | Deposition | - |
| 293 | Dodakundi et al. ^293^ | 2011 | Case report | Histology | Disease | - |
| 294 | Nguyen et al. ^294^ | 2011 | Scoping review | - | - | - |
| 295 | Mukhopadhyay et al. ^295^ | 2011 | Case report | SFA | Deposition | “is the second most common crystal-induced form of arthropathy, frequently seen in the knee, shoulder, wrist, elbow, and ankle” |
| 296 | Di Geso et al. ^296^ | 2011 | Case series | SFA & Imaging | - | - |
| 297 | Niggemeyer et al. ^297^ | 2011 | Case-control study retrospective | Expert opinion | - | - |
| 298 | De Marco et al. ^298^ | 2011 | Case report | Imaging | - | - |
| 299 | Demertzis et al. ^299^ | 2011 | Scoping review | - | Deposition | - |
| 300 | Pandit et al. ^300^ | 2011 | Scoping review | Imaging | - | - |
| 301 | Sklenicka et al. ^301^ | 2011 | Case report | Histology | Deposition | “is a systemic disease that results in crystal deposition within the joint space” |
| 302 | Skeete et al. ^302^ | 2011 | Cohort study retrospective | SFA | - | - |
| 303 | Hernigou et al. ^303^ | 2011 | Cohort study retrospective | SFA & Imaging | - | - |
| 304 | Favero et al. ^304^ | 2011 | Scoping review | - | - | - |
| 305 | Erhardt et al. ^305^ | 2011 | Case report | Histology | - | - |
| 306 | Ea et al. ^306^ | 2011 | Scoping review | - | - | “is considered to be a common aging-related process” |
| 307 | Seybold et al. ^307^ | 2011 | Case report | Histology | Disease | - |
| 308 | Rim et al. ^308^ | 2011 | Case report | Imaging | - | - |
| 309 | Robier et al. ^309^ | 2011 | Cohort study cross-sectional | SFA | - | - |
| 310 | Robier et al. ^310^ | 2011 | Cohort study cross-sectional | - | - | - |
| 311 | Robier et al. ^311^ | 2011 | Case-control study retrospective | SFA | Deposition | - |
| 312 | Robier et al. ^312^ | 2011 | Letter to the editor | SFA | Deposition | - |
| 313 | Rosenthal et al. ^313^ | 2011 | Scoping review | - | - | - |
| 314 | Rosenthal et al. ^314^ | 2011 | Systematic literature review | SFA | Deposition | - |
| 315 | Zhang et al. ^315^ | 2011 | Systematic literature review | - | Deposition | - |
| 316 | Zhang et al. ^316^ | 2011 | Systematic literature review | - | Deposition | - |
| 317 | Yoo et al. ^317^ | 2011 | Case report | Histology | Disease | “is deﬁned as articular deposition of calcium pyrophosphate dihydrate crystals” |
| 318 | Ogawa et al. ^318^ | 2012 | Case report | Histology | Deposition | “the deposition of calcium pyrophosphate dihydrate crystals in the articular or periarticular structures leading to acute, subacute, or chronic joint inflammation” |
| 319 | Levi et al. ^319^ | 2012 | Case report | - | Disease | “is an acute attack of crystal-induced synovitis” |
| 320 | Ivory et al. ^320^ | 2012 | Scoping review | - | Deposition | “is related to a variety of articular manifestations” |
| 321 | Agrawal et al. ^321^ | 2012 | Case report | Histology | Deposition | “a rare joint disease which is characterized by the presence of calcium pyrophosphate dihydrate crystals in the intra-articular and periarticular tissue”; “a rare benign crystalline arthropathy of unknown cause” |
| 322 | Abhishek et al. ^322^ | 2012 | Cohort study cross-sectional | Imaging | Deposition | - |
| 323 | Hunte et al. ^323^ | 2012 | Case report | SFA | - | - |
| 324 | Hujazi et al. ^324^ | 2012 | Cohort study longitudinal | SFA | - | - |
| 325 | Andrés et al. ^325^ | 2012 | Cohort study retrospective | SFA | Deposition | “a prevalent disorder in rheumatology clinics” |
| 326 | Antohe et al. ^326^ | 2012 | Case report | SFA | Disease | - |
| 327 | Namazie et al. ^327^ | 2012 | Case report | Histology | Deposition | - |
| 328 | Lee et al. ^328^ | 2012 | Case report | Histology | Deposition | “is defined as the deposition of CPPD crystals in the articular or periarticular structures that leads to inflammation of the joints” |
| 329 | Bilge et al. ^329^ | 2012 | Case series | Imaging | - | “is a rare inherited metabolic disorder where the chemical calcium pyrophosphate dehydrate is deposited in one or more joints in the body usually the knee is affected” |
| 330 | Beggs et al. ^330^ | 2012 | Case report | - | - | - |
| 331 | Moltó et al. ^331^ | 2012 | Case series | SFA | Deposition | - |
| 332 | Zweifel et al. ^332^ | 2012 | Scoping review | Histology | Deposition | “is a metabolic disorder characterized by non-infectious joint inflammation with intra- or periarticular calcification” |
| 333 | Matsumura et al. ^333^ | 2012 | Case report | Imaging | - | - |
| 334 | Ciancio et al. ^334^ | 2012 | Scoping review | SFA | Deposition | “is characterized by the deposition of calcium pyrophosphate crystals in articular tissues, most commonly fibrocartilage and hyaline cartilage”; “is the third most common inflammatory arthritis” |
| 335 | Magarelli et al. ^335^ | 2012 | Systematic literature review | SFA & Imaging | Deposition | “is characterised by the accumulation of pyrophosphate dihydrate crystals in articular and periarticular tissues; can be classified as sporadic, hereditary or secondary” |
| 336 | Couderc et al. ^336^ | 2012 | Letter to the editor | Imaging | - | - |
| 337 | Diamantopoulos et al. ^337^ | 2012 | Case report | SFA & Imaging | - | - |
| 338 | Minoda et al. ^338^ | 2012 | Case report | Expert opinion | Deposition | - |
| 339 | Di Geso et al. ^339^ | 2012 | Case report | SFA & Imaging | Deposition | “is a metabolic arthropathy caused by the deposition of calcium pyrophosphate crystals in and around joints, especially in hyaline and fibrous cartilage”; “is generally categorized into sporadic, familial, and secondary (metabolic) forms” |
| 340 | Marson et al. ^340^ | 2012 | Scoping review | McCarty criteria | Deposition | - |
| 341 | Matsumura et al. ^341^ | 2012 | Case report | Expert opinion | Deposition | - |
| 342 | Macmullan et al. ^342^ | 2012 | Scoping review | SFA | Deposition | “has the potential to mimic most forms of inflammatory arthritis” |
| 343 | Oda et al. ^343^ | 2012 | Case report | Imaging | - | - |
| 344 | Odate et al. ^344^ | 2012 | Case report | Imaging | - | - |
| 345 | Hosseinian Amiri et al. ^345^ | 2012 | Case report | - | Deposition | - |
| 346 | Gruber et al. ^346^ | 2012 | Case report | SFA | Disease | “is characterized by deposition of calcium pyrophosphate crystals within articular hyaline and fibrocartilages, as well as certain soft tissues” |
| 347 | Hahn et al. ^347^ | 2012 | Case report | Imaging | - | - |
| 348 | Filippou et al. ^348^ | 2012 | Scoping review | - | Deposition | - |
| 349 | Filippou et al. ^349^ | 2012 | Cohort study cross-sectional | Histology | Deposition | - |
| 350 | Filippucci et al. ^350^ | 2012 | Scoping review | - | - | - |
| 351 | Ellabban et al. ^351^ | 2012 | Cohort study longitudinal | SFA | - | “is a common and potentially severe arthropathy in which CPPD crystals deposit within the joint cartilage, fibrocartilage, and juxta-articular tissues leading to calcification” |
| 352 | Ellabban et al. ^352^ | 2012 | Case-control study prospective | McCarty criteria | - | “is a common and potentially severe metabolic arthropathy caused by calcium pyrophosphate dihydrate crystal deposition” |
| 353 | Doghramji et al. ^353^ | 2012 | Scoping review | - | - | - |
| 354 | Seror et al. ^354^ | 2012 | Case report | - | Deposition | - |
| 355 | Popov et al. ^355^ | 2012 | Case report | SFA | Deposition | - |
| 356 | Salar et al. ^356^ | 2012 | Case report | Histology | Deposition | “is a term used to describe symptoms and clinical signs associated with calcium pyrophosphate deposition into the synovium and/or articular fibro or hyaline cartilages” |
| 357 | Reed et al. ^357^ | 2012 | Systematic literature review | SFA | - | - |
| 358 | Srinivasan et al. ^358^ | 2012 | Case report | Histology | Deposition | “is a crystal arthropathy which features calcium pyrophosphate crystal deposition in joint spaces, episodes of synovitis and radiological features of chondrocalcinosis” |
| 359 | Srinivasan et al. ^359^ | 2012 | Case report | Histology | Deposition | “is a crystal arthropathy characterized by the deposition of calcium pyrophosphate crystals in joint spaces, episodes of synovitis, and radiological features of chondrocalcinosis” |
| 360 | Sussmann et al. ^360^ | 2012 | Scoping review | Imaging | - | - |
| 361 | Rho et al. ^361^ | 2012 | Case-control study retrospective | - | Deposition | - |
| 362 | Rosales et al. ^362^ | 2012 | Case report | Imaging | Deposition | - |
| 363 | Terkeltaub et al. ^363^ | 2012 | Scoping review | - | Deposition | - |
| 364 | Wendling et al. ^364^ | 2012 | Case report | Imaging | - | - |
| 365 | Roubille et al. ^365^ | 2013 | Scoping review | - | - | - |
| 366 | Huaiqing et al. ^366^ | 2013 | Case report | - | Disease | “is a metabolic disorder caused by the deposition of calcium pyrophosphate dihydrate in the articular cartilage”; “a relatively common arthritic disorder in the elderly” |
| 367 | Kenzaka et al. ^367^ | 2013 | Case report | SFA | Deposition | “is associated with the deposition of calcium pyrophosphate dihydrate crystals in the articular cartilage and surrounding tissues, which is observed on plain radiography images as calciﬁcations” |
| 368 | Abhishek et al. ^368^ | 2013 | Cohort study cross-sectional | Imaging | Deposition | - |
| 369 | Adinolfi et al. ^369^ | 2013 | Case report | SFA | Deposition | - |
| 370 | Bahk et al. ^370^ | 2013 | Case report | Histology | Deposition | “an inflammatory disease of joints characterized by the presence of crystals in articular space and periarticular tissues” |
| 371 | Barskova et al. ^371^ | 2013 | Cohort study retrospective | SFA | Deposition | “is a metabolic arthropathy due to deposition of calcium pyrophosphate crystals in the joints, most common in articular hyaline or fibrocartilage”; “is a rare disease, most common in the elderly” |
| 372 | Cacciotti et al. ^372^ | 2013 | Case report | McCarty criteria & Histology | Deposition | “is a rare benign inflammatory joint disorder characterized by the presence of calcium pyrophosphate dihydrate crystal in the interarticular and periarticular tissue” |
| 373 | Chakravarty et al. ^373^ | 2013 | Scoping review | - | - | - |
| 374 | Chang et al. ^374^ | 2013 | Cohort study retrospective | Imaging | Deposition | - |
| 375 | Dirim et al. ^375^ | 2013 | Case-control study prospective | - | Deposition | “is the most common cause of crystalline arthropathy” |
| 376 | Morita et al. ^376^ | 2013 | Case report | Imaging | - | - |
| 377 | Damian et al. ^377^ | 2013 | Case report | Histology | - | - |
| 378 | O'Connor et al. ^378^ | 2013 | Scoping review | - | Deposition | - |
| 379 | Oliviero et al. ^379^ | 2013 | Cohort study retrospective | SFA | - | - |
| 380 | Ottaviani et al. ^380^ | 2013 | Case series | - | - | - |
| 381 | Hakozaki et al. ^381^ | 2013 | Case report | SFA | - | - |
| 382 | Godfrin-Valnet et al. ^382^ | 2013 | Case series | Imaging | - | - |
| 383 | Garcia-Gonzalez et al. ^383^ | 2013 | Case report | SFA & Imaging | - | - |
| 384 | Filippou et al. ^384^ | 2013 | Cohort study cross-sectional | Histology | - | - |
| 385 | Filippou et al. ^385^ | 2013 | Cohort study cross-sectional | McCarty criteria | Deposition | - |
| 386 | Filippucci et al. ^386^ | 2013 | Case-control study prospective | McCarty criteria | - | - |
| 387 | Parperis et al. ^387^ | 2013 | Cohort study retrospective | Imaging | Deposition | “is a relatively common arthritic disorder of the elderly” |
| 388 | Suresh et al. ^388^ | 2013 | Scoping review | - | - | - |
| 389 | Robier et al. ^389^ | 2013 | Cohort study cross-sectional | SFA | - | - |
| 390 | Takahashi et al. ^390^ | 2013 | Case report | - | Deposition | - |
| 391 | Russell et al. ^391^ | 2013 | Systematic literature review | Imaging | Deposition | - |
| 392 | Tausche et al. ^392^ | 2013 | Cohort study cross-sectional | SFA | - | - |
| 393 | Touraine et al. ^393^ | 2013 | Cohort study cross-sectional | Imaging | - | - |
| 394 | Uh et al. ^394^ | 2013 | Case report | Imaging | - | - |
| 395 | Ungprasert et al. ^395^ | 2013 | Case report | SFA | Disease | - |
| 396 | Rothschild et al. ^396^ | 2013 | Case report | - | Disease | - |
| 397 | Yamazaki et al. ^397^ | 2013 | Case report | Imaging | Disease | - |
| 398 | Verhoeven et al. ^398^ | 2013 | Letter to the editor | - | - | - |
| 399 | Wakasugi et al. ^399^ | 2013 | Case report | McCarty criteria | Deposition | - |
| 400 | Reis et al. ^400^ | 2014 | Case report | Histology | Deposition | “appears radiographically as chondrocalcinosis involving hyaline and ﬁbrocartilage” |
| 401 | Matsuda et al. ^401^ | 2014 | Case report | - | Disease | - |
| 402 | Kobayashi et al. ^402^ | 2014 | Cohort study longitudinal | SFA | Deposition | - |
| 403 | Jimbo et al. ^403^ | 2014 | Case report | SFA | Disease | “is characterized by crystal deposits in the articular cartilage or synovium” |
| 404 | Abdelsayed et al. ^404^ | 2014 | Case series | Histology | Disease | “is a rare benign crystalline arthropathy of the articulating joints”; “calcium pyrophosphate dihydrate may be deposited asymptomatically, or it may be associated with various chronic and acute symptoms” |
| 405 | Abhishek et al. ^405^ | 2014 | Case-control study retrospective | Expert opinion | Deposition | - |
| 406 | Aichmair et al. ^406^ | 2014 | Case report | Histology | Deposition | - |
| 407 | Abhishek et al. ^407^ | 2014 | Scoping review | SFA | Deposition | “is an umbrella term for all instances of occurrence of calcium pyrophosphate crystals” |
| 408 | Abhishek et al. ^408^ | 2014 | Scoping review | - | Deposition | - |
| 409 | Abhishek et al. ^409^ | 2014 | Case-control study retrospective | Imaging | Deposition | - |
| 410 | Husar-Memmer et al. ^410^ | 2014 | Scoping review | - | - | - |
| 411 | Lee et al. ^411^ | 2014 | Case report | - | Deposition | - |
| 412 | Bruges-Armas et al. ^412^ | 2014 | Case series | SFA & Imaging | Disease | “is caused by calcium pyrophosphate dehydrate crystal deposition. Three main forms of CPPD are usually described: acute pseudogout, inflammatory chronic arthropathy, and osteoarthritis-like disease” |
| 413 | Kuriyama et al. ^413^ | 2014 | Case report | SFA & Imaging | Deposition | - |
| 414 | Kim et al. ^414^ | 2014 | Case report | SFA | Deposition | - |
| 415 | Huang et al. ^415^ | 2014 | Scoping review | - | - | - |
| 416 | Lin et al. ^416^ | 2014 | Case report | SFA | Deposition | “is the third most common inflammatory arthritis, characterized by acute or chronic inflammation caused by deposit of CPPD crystals in articular cartilage and periarticular soft tissues, mostly in knees and wrists” |
| 417 | McQueen et al. ^417^ | 2014 | Scoping review | SFA | Deposition | “is a common form of crystalline arthropathy characterized clinically by a variety of patterns” |
| 418 | Patel et al. ^418^ | 2014 | Case report | - | Deposition | “is the most common form of crystal arthropathy second only to gout” |
| 419 | MuȘetescu et al. ^419^ | 2014 | Case report | SFA | Deposition | “is a metabolic disorder determined by calcium pyrophosphate dihydrate crystal precipitation at the level of intra and periarticular structures […] often manifested through latent joint structural lesions rather than symptomatic arthropathy” |
| 420 | Nguyen et al. ^420^ | 2014 | Scoping review | Imaging | Deposition | - |
| 421 | Klineberg et al. ^421^ | 2014 | Case report | Histology | Deposition | - |
| 422 | Daoussis et al. ^422^ | 2014 | Systematic literature review | SFA | - | - |
| 423 | Souza et al. ^423^ | 2014 | Case report | Imaging | Disease | - |
| 424 | Hanai et al. ^424^ | 2014 | Case report | Histology | - | - |
| 425 | Gutierrez et al. ^425^ | 2014 | Case-control study prospective | SFA | - | “is a disorder characterized by intra-articular and/or periarticular deposition of calcium pyrophosphate (CPP) crystals” |
| 426 | Grobost et al. ^426^ | 2014 | Case report | Histology | - | - |
| 427 | Finckh et al. ^427^ | 2014 | Randomized controlled trial | McCarty criteria | Deposition | “is a very common and occasionally severe arthropathy associated with chondrocalcinosis” |
| 428 | Shirazian et al. ^428^ | 2014 | Cohort study retrospective | Imaging | Deposition | - |
| 429 | Filippou et al. ^429^ | 2014 | Scoping review | - | - | - |
| 430 | Filippucci et al. ^430^ | 2014 | Scoping review | - | - | - |
| 431 | Ea et al. ^431^ | 2014 | Scoping review | - | - | - |
| 432 | Durcan et al. ^432^ | 2014 | Scoping review | - | - | - |
| 433 | Pascart et al. ^433^ | 2014 | Scoping review | - | - | “is widespread and its prevalence increases with age” |
| 434 | Shamil et al. ^434^ | 2014 | Case report | - | Disease | - |
| 435 | Seeger et al. ^435^ | 2014 | Cohort study cross-sectional | Imaging | - | - |
| 436 | Ramonda et al. ^436^ | 2014 | Scoping review | - | - | - |
| 437 | Robier et al. ^437^ | 2014 | Case-control study longitudinal | SFA | - | - |
| 438 | Rosales-Alexander et al. ^438^ | 2014 | Systematic literature review | SFA | - | - |
| 439 | Theiler et al. ^439^ | 2014 | Cohort study cross-sectional | - | Disease | - |
| 440 | Ryu et al. ^440^ | 2014 | Cohort study retrospective | Histology | Deposition | - |
| 441 | Tischler et al. ^441^ | 2014 | Scoping review | - | Deposition | - |
| 442 | Toprak et al. ^442^ | 2014 | Scoping review | - | - | - |
| 443 | Rosenthal et al. ^443^ | 2014 | Scoping review | SFA | Deposition | “comprises a clinically heterogeneous group of arthritides caused by the presence of calcium pyrophosphate crystals in articular tissues” |
| 444 | Viana et al. ^444^ | 2014 | Case report | - | Deposition | - |
| 445 | Wenham et al. ^445^ | 2014 | Systematic literature review | SFA & Imaging & Histology | Deposition | - |
| 446 | Watura et al. ^446^ | 2014 | Case report | - | Disease | - |
| 447 | Kahloune et al. ^447^ | 2015 | Case report | Imaging | Deposition | - |
| 448 | Nakamura et al. ^448^ | 2015 | Case report | Histology | Deposition | - |
| 449 | Inokuchi et al. ^449^ | 2015 | Case report | SFA | - | - |
| 450 | Alix et al. ^450^ | 2015 | Case report | - | Deposition | “is a common disease”; “is characterized by calcium pyrophosphate dehydrate crystal deposition in joints” |
| 451 | Aouba et al. ^451^ | 2015 | Case series | SFA | - | - |
| 452 | Mori et al. ^452^ | 2015 | Case report | SFA | Deposition | - |
| 453 | Bartels et al. ^453^ | 2015 | Case-control study retrospective | McCarty criteria | Deposition | “is a common but understudied form of arthritis occurring in approximately 20% of adults over age 80 and contributing to nearly 25% of knee osteoarthritis in older adults” |
| 454 | Boroda et al. ^454^ | 2015 | Case report | - | - | - |
| 455 | Borowski et al. ^455^ | 2015 | Case report | - | Deposition | “affects para-articular soft tissues or articular components such as cartilage and causes painful arthritis” |
| 456 | Koda et al. ^456^ | 2015 | Case report | - | - | - |
| 457 | Brennan et al. ^457^ | 2015 | Case report | SFA | Deposition | “is a rheumatological disorder with various symptoms caused by the deposition of calcium pyrophosphate dihydrate crystals in the articular cartilage and synovial fluid” |
| 458 | Lomax et al. ^458^ | 2015 | Case series | Histology & Expert opinion | Deposition | “is a metabolic disorder characterized by soft tissue calcific deposits formed primarily in articular cartilage” |
| 459 | Löffler et al. ^459^ | 2015 | Cohort study cross-sectional | SFA | Deposition | “is a crystal-related joint disease that often present with acute monoarthritis or oligoarthritis and caused by synovial deposition of calcium pyrophosphate crystals” |
| 460 | Chang et al. ^460^ | 2015 | Case report | Imaging | Deposition | - |
| 461 | Checa et al. ^461^ | 2015 | Cohort study cross-sectional | Histology | Disease | “is a common adult chronic arthropathy with a wide pathologic spectrum” |
| 462 | Maravic et al. ^462^ | 2015 | Cohort study retrospective | - | Deposition | “is a microcrystal-related diseases secondary to calcium pyrophosphate deposition” |
| 463 | Miksanek et al. ^463^ | 2015 | Scoping review | McCarty criteria | Deposition | “is a common and clinically heterogeneous form of arthritis caused by the deposition of calcium pyrophosphate (CPP) crystals in articular tissues” |
| 464 | Cimbek et al. ^464^ | 2015 | Case report | - | Deposition | “is the umbrella term that includes acute calcium pyrophosphate crystal arthritis, chronic calcium pyrophosphate crystal inflammatory arthritis and osteoarthritis with calcium pyrophosphate dehydrate crystal deposition disease” |
| 465 | Moses et al. ^465^ | 2015 | Case report | Imaging | Deposition | - |
| 466 | Couturier et al. ^466^ | 2015 | Case-control study prospective | Imaging | Deposition | - |
| 467 | Kocyigit et al. ^467^ | 2015 | Case report | SFA | Deposition | “is one of the most common crystal-induced arthropathies, the acutest form of arthritis in the elderly” |
| 468 | Laviv et al. ^468^ | 2015 | Case report | Histology | Deposition | “is a rare benign crystalline arthropathy […] with a predilection for joints with fibrocartilage rather than hyaline cartilage, most commonly affecting the knee meniscus and the triangular ligament of the wrist” |
| 469 | Di Carlo et al. ^469^ | 2015 | Case report | - | - | - |
| 470 | Ochoa Escudero et al. ^470^ | 2015 | Case series | Histology | Disease | - |
| 471 | Oka et al. ^471^ | 2015 | Case series | Imaging | Deposition | - |
| 472 | Ottaviani et al. ^472^ | 2015 | Cohort study retrospective | SFA | - | - |
| 473 | Singh et al. ^473^ | 2015 | Scoping review | SFA | - | - |
| 474 | Hong et al. ^474^ | 2015 | Case report | - | Deposition | “is a common rheumatic arthropathy caused by the deposition of calcium pyrophosphate crystals in and around joints, especially in knee, wrist, and hip joints” |
| 475 | Harato et al. ^475^ | 2015 | Cohort study longitudinal | SFA | - | - |
| 476 | Grassi et al. ^476^ | 2015 | Scoping review | - | - | - |
| 477 | Gamon et al. ^477^ | 2015 | Meta-analysis | McCarthy criteria | Deposition | “Is one of the two main crystal-related arthropathies associated with gout, and one of the most common inflammatory joint diseases. It involves the formation of chronic or acute calcium pyrophosphate crystals in joints, fibrous cartilage, tendons or bursa/ recesses” |
| 478 | Pascual et al. ^478^ | 2015 | Scoping review | SFA | - | - |
| 479 | Patel et al. ^479^ | 2015 | Case report | - | - | - |
| 480 | Pollet et al. ^480^ | 2015 | Cohort study longitudinal | SFA | Deposition | - |
| 481 | Ramonda et al. ^481^ | 2015 | Scoping review | - | Deposition | - |
| 482 | Strub et al. ^482^ | 2015 | Cohort study longitudinal | SFA | Disease | - |
| 483 | Tagoe et al. ^483^ | 2015 | Cohort study cross-sectional | SFA | Disease | - |
| 484 | Takeda et al. ^484^ | 2015 | Case report | Imaging | Deposition | - |
| 485 | Taljanovic et al. ^485^ | 2015 | Scoping review | - | Deposition | - |
| 486 | Ruban et al. ^486^ | 2015 | Case report | - | - | - |
| 487 | Zufferey et al. ^487^ | 2015 | Case-control study longitudinal | SFA & Imaging | Deposition | - |
| 488 | Zhang et al. ^488^ | 2015 | Case report | Imaging | Deposition | - |
| 489 | Zaman et al. ^489^ | 2015 | Case report | SFA | Deposition | - |
| 490 | Yamada et al. ^490^ | 2015 | Case report | Imaging | Deposition | - |
| 491 | Ward et al. ^491^ | 2015 | Case report | Imaging | Deposition | “is a common etiology of crystalline arthropathy” |
| 492 | Jungraithmayr et al. ^492^ | 2016 | Case report | - | Deposition | - |
| 493 | Kobayashi et al. ^493^ | 2016 | Case report | Histology | Deposition | - |
| 494 | Rosen et al. ^494^ | 2016 | Case report | McCarty criteria | Deposition | - |
| 495 | Krochak et al. ^495^ | 2016 | Case report | Histology | Deposition | “is a general term used for several musculoskeletal maladies caused by ectopic calcium salt crystallization that can be asymptomatic or associated with a number of clinical syndromes” |
| 496 | Afzal et al. ^496^ | 2016 | Case report | SFA | Deposition | “is a crystal-induced arthropathy characterized by the deposition of calcium pyrophosphate dihydrate crystals in synovial fluid, menisci, or articular cartilage” |
| 497 | Abhishek et al. ^497^ | 2016 | Case-control study retrospective | Imaging | Deposition | - |
| 498 | Abhishek et al. ^498^ | 2016 | Scoping review | - | Deposition | “is frequently asymptomatic and manifests as articular chondrocalcinosis on imaging studies” |
| 499 | Abhishek et al. ^499^ | 2016 | Systematic literature review | McCarty criteria | Deposition | “occurs mainly in the elderly”; “can present with acute CPP crystal arthritis or chronic arthropathy with structural changes of osteoarthritis, or may be asymptomatic presenting as an incidental finding of chondrocalcinosis on imaging studies” |
| 500 | Iqbal et al. ^500^ | 2016 | Case report | Imaging | - | - |
| 501 | Iqbal et al. ^501^ | 2016 | Case report | Imaging | - | - |
| 502 | Akkoç et al. ^502^ | 2016 | Case report | Imaging | Deposition | “is a crystal arthropathy caused by the deposition of calcium pyrophosphate dehydrate in and around joints particularly in articular cartilage and fibrocartilage” |
| 503 | Hubert et al. ^503^ | 2016 | Cohort study cross-sectional | Imaging | - | - |
| 504 | Hiroyasu Nakano et al. ^504^ | 2016 | Case report | Imaging | Deposition | - |
| 505 | Cammelli et al. ^505^ | 2016 | Case report | Imaging | - | - |
| 506 | Hoxha et al. ^506^ | 2016 | Case report | SFA | - | - |
| 507 | Soloway et al. ^507^ | 2016 | Case report | SFA | Deposition | - |
| 508 | Kurihara et al. ^508^ | 2016 | Case report | Imaging | - | - |
| 509 | Dietvorst et al. ^509^ | 2016 | Case report | SFA | - | - |
| 510 | Cozzani et al. ^510^ | 2016 | Case report | SFA | Disease | “is the most common cause of chondrocalcinosis, a form of microcrystalline arthritis induced by calcium pyrophosphate crystal deposits both in and around the joints” |
| 511 | Dadlani et al. ^511^ | 2016 | Case report | Imaging | - | - |
| 512 | Ng et al. ^512^ | 2016 | Case report | Histology | - | “is characterized by the deposition of CPPD crystals in hyaline cartilage and fibrocartilage” |
| 513 | Omoumi et al. ^513^ | 2016 | Scoping review | Imaging | Deposition | - |
| 514 | Pappu et al. ^514^ | 2016 | Scoping review | Imaging | Deposition | - |
| 515 | Park et al. ^515^ | 2016 | Case report | Histology | - | “is an inflammatory disease of the joints characterized by the periarticular deposition of CPPD crystals, which most commonly occurs in fibrocartilage and hyaline cartilage” |
| 516 | Sivera et al. ^516^ | 2016 | Scoping review | Imaging | Disease | “is an underrecognized cause of musculoskeletal symptoms” |
| 517 | Horino et al. ^517^ | 2016 | Case report | - | - | - |
| 518 | Higgins et al. ^518^ | 2016 | Scoping review | - | - | “is characterized by calcium pyrophosphate dihydrate crystal deposits in articular joints” |
| 519 | Gersing et al. ^519^ | 2016 | Case-control study prospective | Imaging | - | - |
| 520 | Glanville et al. ^520^ | 2016 | Scoping review | - | - | - |
| 521 | Galed-Placed et al. ^521^ | 2016 | Case report | SFA | - | - |
| 522 | Galozzi et al. ^522^ | 2016 | Cohort study retrospective | SFA | - | - |
| 523 | Fung et al. ^523^ | 2016 | Case report | Expert opinion | - | - |
| 524 | Frallonardo et al. ^524^ | 2016 | Cohort study cross-sectional | SFA | - | - |
| 525 | Filippou et al. ^525^ | 2016 | Meta-analysis | McCarty criteria | Deposition | “is the third most common inflammatory arthritis and its prevalence grows with ageing” |
| 526 | Filippou et al. ^526^ | 2016 | Cohort study cross-sectional | Histology | Deposition | “is one of the most frequent arthropathies among the elderly”; “is the umbrella term for all instances of CPP crystal occurrence” |
| 527 | Filippou et al. ^527^ | 2016 | Cohort study cross-sectional | Histology | Deposition | - |
| 528 | Escrivá-Fornés et al. ^528^ | 2016 | Case report | SFA | - | - |
| 529 | Patel et al. ^529^ | 2016 | Cohort study retrospective | Imaging | Deposition | - |
| 530 | Pawelek et al. ^530^ | 2016 | Case report | SFA | Deposition | - |
| 531 | Stensby et al. ^531^ | 2016 | Case-control study longitudinal | Imaging | Deposition | - |
| 532 | Tagami et al. ^532^ | 2016 | Case report | Imaging | Deposition | - |
| 533 | Tajima et al. ^533^ | 2016 | Case series | Imaging | Deposition | - |
| 534 | Rheinboldt et al. ^534^ | 2016 | Systematic literature review | Imaging | Deposition | “is a metabolic arthropathy secondary to the precipitation of calcium pyrophosphate dihydrate crystals in the synovial and periarticular soft tissues” |
| 535 | Ryosuke et al. ^535^ | 2016 | Case report | SFA & Imaging & Histology | Deposition | “is a disease that involves the deposition of calcium pyrophosphate dehydrate crystals in the affected joint” |
| 536 | Ruta et al. ^536^ | 2016 | Case series | Imaging | Deposition | - |
| 537 | Rosenthal et al. ^537^ | 2016 | Systematic literature review | SFA | Deposition | “is arthritis caused by calcium pyrophosphate crystals” |
| 538 | Zabotti et al. ^538^ | 2016 | Case report | SFA & Imaging | Deposition | - |
| 539 | Verhoeven et al. ^539^ | 2016 | Case report | Imaging | - | - |
| 540 | Yahia et al. ^540^ | 2016 | Cohort study retrospective | SFA | - | - |
| 541 | Wu et al. ^541^ | 2016 | Systematic literature review | Imaging & Histology | Disease | - |
| 542 | Wadhwa et al. ^542^ | 2016 | Scoping review | Imaging | Deposition | “may be hereditary or sporadic, with the former usually seen in elderly patients and the latter showing a preponderance towards young women”; “five common clinical patterns – asymptomatic, pseudo-gout, pseudo-rheumatoid, pseudo-osteoarthritic and pseudo-neuropathic joint disease pattern” |
| 543 | Joshi et al. ^543^ | 2017 | Case report | SFA & Imaging | Deposition | “one of the most common forms of inflammatory arthritis” |
| 544 | Kudoh et al. ^544^ | 2017 | Case report | Histology | Disease | - |
| 545 | Lim et al. ^545^ | 2017 | Case report | Histology | Deposition | - |
| 546 | Jiang et al. ^546^ | 2017 | Case report | Imaging | - | - |
| 547 | Jens et al. ^547^ | 2017 | Scoping review | - | - | - |
| 548 | Jacques et al. ^548^ | 2017 | Scoping review | - | Deposition | “is caused by the deposition of calcium pyrophosphate crystals in joints” |
| 549 | Jacobson et al. ^549^ | 2017 | Scoping review | - | Disease | - |
| 550 | Imamura et al.^550^ | 2017 | Case report | Imaging | - | - |
| 551 | Inoue et al. ^551^ | 2017 | Case report | Imaging | - | - |
| 552 | Ankli et al. ^552^ | 2017 | Cohort study cross-sectional | SFA | Disease | “is a common arthritis” |
| 553 | Kleiber Balderrama et al. ^553^ | 2017 | Cohort study cross-sectional | SFA | Disease | “is a common cause of acute and chronic arthritis” |
| 554 | Awan et al. ^554^ | 2017 | Case report | SFA | Deposition | “is a common arthritic disorder among elderly patients” |
| 555 | Baudart et al. ^555^ | 2017 | Case report | Imaging | Deposition | “is a crystal-related arthropathy and one of the most common inflammatory joint disease”; “is characterized by the presence of calcium pyrophosphate dihydrate crystals within the cartilage of the joints and fibrocartilage” |
| 556 | Berendsen et al. ^556^ | 2017 | Cohort study cross-sectional | SFA | - | - |
| 557 | Boumans et al. ^557^ | 2017 | Case-control study longitudinal | Imaging | - | - |
| 558 | Kohno et al. ^558^ | 2017 | Case report | SFA | Deposition | - |
| 559 | Bridges et al. ^559^ | 2017 | Case report | Imaging | - | - |
| 560 | Buckens et al. ^560^ | 2017 | Scoping review | Imaging | Disease | “is characterized by the deposition of calcium pyrophosphate crystals in and around joints causing inflammation sometimes with paroxysmal goutlike symptoms” |
| 561 | Bui et al. ^561^ | 2017 | Case report | SFA | Deposition | - |
| 562 | Can et al. ^562^ | 2017 | Systematic literature review | Imaging | - | - |
| 563 | Carlson et al. ^563^ | 2017 | Scoping review | SFA | Deposition | - |
| 564 | Priesand et al. ^564^ | 2017 | Case report | SFA | Deposition | - |
| 565 | Carpenter et al. ^565^ | 2017 | Case report | SFA | Disease | - |
| 566 | Castro et al. ^566^ | 2017 | Case report | SFA | Disease | - |
| 567 | Chen et al. ^567^ | 2017 | Case report | SFA | Disease | - |
| 568 | Couto et al. ^568^ | 2017 | Cohort study cross-sectional | Imaging | - | “is characterized by the deposition of calcium-containing crystals in articular cartilage, synovial membranes and in periarticular soft tissues” |
| 569 | Couture et al. ^569^ | 2017 | Case series | SFA & Imaging | Deposition | - |
| 570 | Di Matteo et al. ^570^ | 2017 | Case-control study longitudinal | McCarty criteria | Deposition | “is a crystal related arthropathy characterised by deposition of calcium pyrophosphate dihydrate (CPP) crystals at articular and periarticular structures” |
| 571 | Karimzadeh et al. ^571^ | 2017 | Cohort study cross-sectional | SFA | - | - |
| 572 | Singh et al. ^572^ | 2017 | Scoping review | SFA | - | - |
| 573 | Han et al. ^573^ | 2017 | Cohort study longitudinal | Expert opinion | - | - |
| 574 | Ferreyra et al. ^574^ | 2017 | Cohort study retrospective | SFA | - | - |
| 575 | Filippou et al. ^575^ | 2017 | Cohort study cross-sectional | SFA | - | “is one of the most common arthropathies of the elderly” |
| 576 | Shikino et al. ^576^ | 2017 | Case report | Imaging | Disease | - |
| 577 | Shapiro et al. ^577^ | 2017 | Scoping review | - | - | - |
| 578 | Petit et al. ^578^ | 2017 | Case report | Histology | Deposition | - |
| 579 | Stainsby et al. ^579^ | 2017 | Case report | Imaging | Disease | - |
| 580 | Takahashi et al. ^580^ | 2017 | Cohort study cross-sectional | Imaging | Deposition | - |
| 581 | Roddy et al. ^581^ | 2017 | Case-control study retrospective | Histology | Deposition | “is a common idiopathic age-related phenomenon. Clinical presentations are varied and include asymptomatic radiographic chondrocalcinosis, chronic arthropathy, and, most dramatically, acute attacks of joint pain and swelling” |
| 582 | Tedeschi et al. ^582^ | 2017 | Case report | SFA & Imaging | Deposition | “causes a spectrum of clinical presentations including asymptomatic chondrocalcinosis, acute CPP crystal arthritis (pseudogout), and chronic CPP inflammatory arthritis” |
| 583 | Testa et al. ^583^ | 2017 | Case series | SFA | - | - |
| 584 | Rothschild et al. ^584^ | 2017 | Scoping review | Histology | Disease | - |
| 585 | Zeng et al. ^585^ | 2017 | Scoping review | Imaging | Deposition | - |
| 586 | Younis et al. ^586^ | 2017 | Case report | - | Deposition | - |
| 587 | Villion et al. ^587^ | 2017 | Cohort study retrospective | SFA | Disease | - |
| 588 | Wickrematilake et al. ^588^ | 2017 | Case series | - | Deposition | “is characterized by the deposition of calcium pyrophosphate dihydrate crystals in the articular cartilage visualized as intra-articular calcifications with identification of the crystals in the synovial fluid and an acute arthropathy called pseudogout” |
| 589 | Watanabe et al. ^589^ | 2017 | Case report | SFA | Deposition | - |
| 590 | Takashi et al. ^590^ | 2018 | Cohort study longitudinal | SFA | Deposition | “is a common rheumatologic disorder that induce acute arthritis” |
| 591 | Muangchan et al. ^591^ | 2018 | Cohort study cross-sectional | SFA | Deposition | “is a common cause of crystal-induced acute non-infectious arthritis presented in inpatient and outpatient settings” |
| 592 | Masmoudi et al. ^592^ | 2018 | Case report | - | Disease | - |
| 593 | McCarthy et al. ^593^ | 2018 | Scoping review | SFA | Deposition | - |
| 594 | Abhishek et al. ^594^ | 2018 | Scoping review | Expert opinion | Deposition | - |
| 595 | Abhishek et al. ^595^ | 2018 | Scoping review | - | - | - |
| 596 | Hubert et al. ^596^ | 2018 | Cohort study cross-sectional | Imaging | - | - |
| 597 | Andrés et al. ^597^ | 2018 | Scoping review | - | Disease | “is a common disease and a frequent cause of visits to the emergency services” |
| 598 | Argyropoulos et al. ^598^ | 2018 | Case report | - | Disease | - |
| 599 | Martens et al. ^599^ | 2018 | Case report | SFA | - | - |
| 600 | Kwon et al. ^600^ | 2018 | Case report | Histology | Deposition | - |
| 601 | Ledingham et al. ^601^ | 2018 | Case report | Imaging & Expert opinion | Deposition | - |
| 602 | Pavic et al. ^602^ | 2018 | Cohort study retrospective | - | - | - |
| 603 | Chang et al. ^603^ | 2018 | Scoping review | - | Deposition | - |
| 604 | Chiba et al. ^604^ | 2018 | Cohort study retrospective | Imaging | Deposition | - |
| 605 | Cho et al. ^605^ | 2018 | Cohort study longitudinal | Imaging | Deposition | - |
| 606 | Colaco et al. ^606^ | 2018 | Case report | SFA | Deposition | - |
| 607 | Conway et al. ^607^ | 2018 | Scoping review | SFA | - | - |
| 608 | Ottaviani et al. ^608^ | 2018 | Case report | Imaging | Deposition | - |
| 609 | Coutier et al. ^609^ | 2018 | Case report | Imaging | - | - |
| 610 | Curzi et al. ^610^ | 2018 | Scoping review | - | - | “is a crystal-induced arthropathy characterized by calcium pyrophosphate dihydrate crystal deposition in the connective tissues and it is strictly correlated to aging” |
| 611 | Lans et al. ^611^ | 2018 | Scoping review | SFA | Deposition | - |
| 612 | Mijola et al. ^612^ | 2018 | Case report | Imaging | Deposition | - |
| 613 | Madhavan et al. ^613^ | 2018 | Case series | Imaging | Deposition | - |
| 614 | Delpont et al. ^614^ | 2018 | Scoping review | - | Disease | - |
| 615 | Heck et al. ^615^ | 2018 | Case report | Imaging | Disease | - |
| 616 | Hatayama et al. ^616^ | 2018 | Case report | SFA | - | - |
| 617 | Goel et al. ^617^ | 2018 | Case report | Imaging | - | - |
| 618 | Grana et al. ^618^ | 2018 | Case report | SFA | - | - |
| 619 | Fuentes-Martinez et al. ^619^ | 2018 | Case report | SFA | - | “occurs in elderly patients particularly in the knee joint” |
| 620 | Freire et al. ^620^ | 2018 | Scoping review | - | - | “is the archetype of articular calcifications and the most frequent cause of crystal-induced arthropathies” |
| 621 | Sidari et al. ^621^ | 2018 | Scoping review | SFA | Deposition | “is an umbrella term to refer to all occurrences of CPP crystals” |
| 622 | Forien et al. ^622^ | 2018 | Case-control study prospective | - | Disease | “is a joint disease caused by deposition of calcium pyrophosphate crystals” |
| 623 | Frallonardo et al. ^623^ | 2018 | Cohort study cross-sectional | SFA | - | - |
| 624 | Filippou et al. ^624^ | 2018 | Cohort study cross-sectional | SFA | - | “is one of the most common arthropathies in the elderly” |
| 625 | Sano et al. ^625^ | 2018 | Cohort study retrospective | SFA & Imaging | Disease | - |
| 626 | Subramanian et al. ^626^ | 2018 | Case report | Other | - | “is a metabolic arthropathy which results from deposition of calcium pyrophosphate crystals in and around joints especially the hyaline cartilage and disc material” |
| 627 | Tai et al. ^627^ | 2018 | Case report | McCarty criteria | Deposition | - |
| 628 | Schlee et al. ^628^ | 2018 | Scoping review | SFA | - | - |
| 629 | Rigsbee et al. ^629^ | 2018 | Case report | Imaging | Deposition | - |
| 630 | Takahashi et al. ^630^ | 2018 | Scoping review | - | Deposition | - |
| 631 | Tanikawa et al. ^631^ | 2018 | Cohort study cross-sectional | SFA | - | - |
| 632 | Tedeschi et al. ^632^ | 2018 | Cohort study retrospective | SFA | Deposition | - |
| 633 | Sabchyshyn et al. ^633^ | 2018 | Cohort study longitudinal | SFA & Imaging | Deposition | “a common, often un-recognized form of acute and chronic arthritis” |
| 634 | Rothschild et al. ^634^ | 2018 | Cohort study retrospective | - | Deposition | - |
| 635 | Zamudio-Cuevas et al. ^635^ | 2018 | Scoping review | SFA | Deposition | - |
| 636 | Yamamura et al. ^636^ | 2018 | Case report | SFA & Imaging | Deposition | “is an inflammatory arthritis produced by the deposition of calcium pyrophosphate crystals in articular and periarticular soft tissues” |
| 637 | Vele et al. ^637^ | 2018 | Cohort study cross-sectional | SFA | Deposition | - |
| 638 | Vellone et al. ^638^ | 2018 | Case report | Expert opinion | - | - |
| 639 | Wu et al. ^639^ | 2018 | Case report | Imaging | Deposition | - |
| 640 | Wold et al. ^640^ | 2018 | Case-control study retrospective | - | Deposition | - |
| 641 | Wada et al. ^641^ | 2018 | Case report | - | Deposition | - |
| 642 | Guohua et al. ^642^ | 2019 | Case report | Histology | Disease | “is a rare inflammatory disease of the joints characterized by the presence of calcium pyrophosphate dehydrate crystals in the intra-articular tissue most commonly fibrocartilage and hyaline cartilage” |
| 643 | Kaffel et al. ^643^ | 2019 | Case report | Imaging | Deposition | - |
| 644 | Joyce et al. ^644^ | 2019 | Case series | Imaging | Disease | - |
| 645 | Jeong et al. ^645^ | 2019 | Cohort study cross-sectional | SFA | - | - |
| 646 | Iwasaki et al. ^646^ | 2019 | Case series | Expert opinion | Deposition | “is an arthritis related condition that is caused by the deposition of calcium pyrophosphate crystals in the joints” |
| 647 | Iqbal et al. ^647^ | 2019 | Scoping review | - | Disease | “is caused by the deposition of calcium pyrophosphate crystals in the articular cartilage, resulting in inflammation and degenerative changes in the affected joint” |
| 648 | Huang et al. ^648^ | 2019 | Scoping review | - | - | “is a group of chronic conditions that are caused by the deposition of calcium pyrophosphate dihydrate crystals in and around the joints” |
| 649 | Amouzougan et al. ^649^ | 2019 | Case report | Imaging & Histology | - | - |
| 650 | Andrés et al. ^650^ | 2019 | Cohort study cross-sectional | - | - | - |
| 651 | Ariyawatkul et al. ^651^ | 2019 | Case-control study prospective | Histology | Disease | “is the third most common type of inflammatory arthritis” |
| 652 | Lim et al. ^652^ | 2019 | Case report | Imaging | - | “is one of the crystal arthropathies, caused by the deposition of CPPD crystals mainly at the knees, wrists and hands joint” |
| 653 | Baillet et al. ^653^ | 2019 | Cohort study retrospective | SFA & Imaging | Disease | - |
| 654 | Bangert et al. ^654^ | 2019 | Case report | SFA | - | - |
| 655 | Bansal et al. ^655^ | 2019 | Case report | Imaging | - | “majority of individuals with CPPD deposition are asymptomatic whereas others can present either with acute episodic mono- or oligo-arthritis involving large joints (knee ankle or wrists) or with chronic arthropathy” |
| 656 | Becce et al. ^656^ | 2019 | Scoping review | SFA & Imaging | Deposition | “is the umbrella term for all instances of CPP crystal occurrence” |
| 657 | Becce et al. ^657^ | 2019 | Scoping review | SFA | Deposition | “is the third most common inflammatory arthritis” |
| 658 | Lee et al. ^658^ | 2019 | Cohort study longitudinal | SFA & Imaging | Deposition | “is the third most common inflammatory arthritis caused by calcium pyrophosphate crystals” |
| 659 | Soma et al. ^659^ | 2019 | Case report | Imaging | Deposition | “is a condition in which calcium pyrophosphate dihydrate crystal is deposited in joint cartilage and ligaments” |
| 660 | Sousa et al. ^660^ | 2019 | Case report | Imaging | Deposition | - |
| 661 | Sadiq et al. ^661^ | 2019 | Case report | - | Deposition | “is the accumulation of calcium pyrophosphate dihydrate crystals in the intra-articular and periarticular tissues” |
| 662 | Chouk et al. ^662^ | 2019 | Case-control study longitudinal | SFA & Imaging | Disease | - |
| 663 | Cooper et al. ^663^ | 2019 | Case report | Imaging | - | - |
| 664 | Rothschild et al. ^664^ | 2019 | Letter to the editor | - | Disease | “is a disorder long recognized as inflammatory in character” |
| 665 | Khmelinskii et al. ^665^ | 2019 | Case report | SFA | Deposition | - |
| 666 | Di Matteo et al. ^666^ | 2019 | Cohort study longitudinal | SFA | Deposition | “is a microcrystalline arthropathy caused by the deposition of calcium pyrophosphate crystals within articular and periarticular tissues” |
| 667 | Maloney et al. ^667^ | 2019 | Case report | SFA | - | “is a chronic idiopathic articular disease that predominantly affects elderly patients, caused by a systemic deposition of calcium pyrophosphate crystals in the articular and hyaline joint cartilage” |
| 668 | Kim et al. ^668^ | 2019 | Case report | SFA | Deposition | “is a neutrophil-driven autoinflammatory disorder” |
| 669 | Northrup et al. ^669^ | 2019 | Case report | - | Deposition | - |
| 670 | Moshrif et al. ^670^ | 2019 | Case series | Imaging | Deposition | “represents the deposition of calcium pyrophosphate dihydrate crystals almost exclusively into articular fibrocartilage and hyaline cartilage, where it is the most common cause of chondrocalcinosis” |
| 671 | Mohammed et al. ^671^ | 2019 | Cohort study cross-sectional | Synovial fluid analysis & Imaging | Deposition | “is a joint disease related to abnormal intra-cartilaginous precipitates of crystals” |
| 672 | Mizokami et al. ^672^ | 2019 | Case series | Expert opinion | Deposition | “is the most common non-gout crystal arthropathy and is by the EULAR proposed to be an umbrella term for all calcium pyrophosphate crystal arthropathies” |
| 673 | Loizidis et al. ^673^ | 2019 | Case report | Histology | - | - |
| 674 | De Jong et al. ^674^ | 2019 | Case report | Histology | Disease | - |
| 675 | De la Garza-Montaño et al. ^675^ | 2019 | Cohort study retrospective | SFA & Histology | Disease | - |
| 676 | Paalanen et al. ^676^ | 2019 | Cohort study cross-sectional | SFA | Deposition | - |
| 677 | Slostad et al. ^677^ | 2019 | Case report | Imaging | Disease | - |
| 678 | Hosu et al. ^678^ | 2019 | Scoping review | - | Deposition | - |
| 679 | Ho et al. ^679^ | 2019 | Case report | - | Disease | - |
| 680 | Hameed et al. ^680^ | 2019 | Cohort study longitudinal | - | Disease | - |
| 681 | Gumucio et al. ^681^ | 2019 | Case report | Expert opinion | - | - |
| 682 | Guillot et al. ^682^ | 2019 | Randomized controlled trial | SFA | - | - |
| 683 | Halupa et al. ^683^ | 2019 | Case report | SFA | - | - |
| 684 | Hakozaki et al. ^684^ | 2019 | Case report | SFA | - | - |
| 685 | Hajri et al. ^685^ | 2019 | Case report | Imaging | Deposition | - |
| 686 | George et al. ^686^ | 2019 | Cohort study retrospective | - | - | - |
| 687 | Galeano-Valle et al. ^687^ | 2019 | Case report | - | - | - |
| 688 | Gama et al. ^688^ | 2019 | Case report | SFA | - | - |
| 689 | García-Fontana et al. ^689^ | 2019 | Case series | Expert opinion | - | - |
| 690 | Gao et al. ^690^ | 2019 | Case report | - | Deposition | “is a common inflammatory pyrophosphate arthropathy, associated with degenerative osteoarthritis” |
| 691 | Finkenstaedt et al. ^691^ | 2019 | Cohort study cross-sectional | Imaging | Deposition | “is a common condition particularly in the elderly population that leads to chondrocalcinosis affecting the fibrocartilaginous menisci as one site of predilection”; “is most commonly asymptomatic, but can occasionally present as acute CPPD-associated arthritis and is then known as pseudogout” |
| 692 | Ellis et al. ^692^ | 2019 | Scoping review | - | - | - |
| 693 | Pascart et al. ^693^ | 2019 | Cohort study retrospective | SFA | Deposition | - |
| 694 | Stamp et al. ^694^ | 2019 | Case report | Imaging | - | - |
| 695 | Sahu et al. ^695^ | 2019 | Case report | SFA | Disease | - |
| 696 | Scheldeman et al. ^696^ | 2019 | Case report | Imaging | Deposition | - |
| 697 | Tedeschi et al. ^697^ | 2019 | Scoping review | - | Deposition | “is a crystalline arthritis that predominantly affects older adults” |
| 698 | Thomas et al. ^698^ | 2019 | Cohort study retrospective | - | - | - |
| 699 | Turaga et al. ^699^ | 2019 | Case report | SFA | Deposition | - |
| 700 | Ujihara et al. ^700^ | 2019 | Case report | Expert opinion | - | - |
| 701 | Urits et al. ^701^ | 2019 | Case report | Imaging | Deposition | - |
| 702 | Zell et al.^702^ | 2019 | Systematic literature review | SFA | - | - |
| 703 | Vasishta et al. ^703^ | 2019 | Case report | SFA & Imaging | - | - |
| 704 | Wang et al. ^704^ | 2019 | Case report | SFA | Deposition | “is a type of inflammatory joint disease in the elderly” |
| 705 | Wang et al. ^705^ | 2019 | Systematic literature review | Imaging | Deposition | - |
| 706 | Desmarais et al. ^706^ | 2019 | Cohort study retrospective | - | Disease | - |
| 707 | Saylısoy et al. ^707^ | 2020 | Case report | Imaging | Deposition | “a crystal arthropathy in which crystals are deposited in the joints and/or soft tissues lead to a variety of articular and periarticular disorders”; “Acute CPPD arthritis is the most notable manifestation of CPPD disease, while CPPD can also result in asymptomatic disease or chronic CPPD arthropathies” |
| 708 | Ojemolon et al. ^708^ | 2020 | Case report | SFA & Imaging | Deposition | - |
| 709 | Konig et al. ^709^ | 2020 | Case report | - | Deposition | - |
| 710 | Kimura et al. ^710^ | 2020 | Case report | Imaging & Histology | Deposition | - |
| 711 | McCarthy ^711^ | 2020 | Scoping review | - | Deposition | - |
| 712 | Abou-Foul et al. ^712^ | 2020 | Case report | Imaging & Histology | Deposition | “is a well described rheumatologic condition characterized by non-infectious arthropathy with intra- or periarticular calcification” |
| 713 | Hubert et al. ^713^ | 2020 | Cohort study cross-sectional | Imaging | - | - |
| 714 | Aliste-Fernández et al. ^714^ | 2020 | Case-control study longitudinal | - | Deposition | - |
| 715 | Andrés et al. ^715^ | 2020 | Scoping review | SFA & Imaging | - | “is a neglected disorder common in clinics and wards” |
| 716 | Kleyer et al. ^716^ | 2020 | Case report | - | Disease | “is a quite common cause of inflammatory arthritis in an elderly population” |
| 717 | Moon et al. ^717^ | 2020 | Case series | Histology | Deposition | “is an inflammatory arthropathy characterized by the presence of calcium pyrophosphate crystals in articular or periarticular tissues”; “is a common cause of inflammatory arthropathy in older patients, most commonly affecting peripheral joints such as the knees and wrists” |
| 718 | Loro et al. ^718^ | 2020 | Case report | Histology | Deposition | “is a rare disease in the temporomandibular joint, that usually affects other joints, and patients are usually over the age of 60” |
| 719 | Awisat et al. ^719^ | 2020 | Cohort study longitudinal | Imaging | Disease | - |
| 720 | Bousson et al. ^720^ | 2020 | Case report | - | - | - |
| 721 | McCarron et al. ^721^ | 2020 | Case report | - | Deposition | - |
| 722 | Cai et al. ^722^ | 2020 | Scoping review | - | Deposition | “is a manifestation of calcium pyrophosphate deposition”; “is an umbrella term that includes acute calcium pyrophosphate crystal arthritis - chronic CPP crystal inflammatory arthritis - osteoarthritis with CPPD - and asymptomatic chondrocalcinosis” |
| 723 | Catelli et al. ^723^ | 2020 | Case report | SFA | - | - |
| 724 | Hsieh et al. ^724^ | 2020 | Case report | - | Disease | “is a common inflammatory arthritis in the elderly due to calcium pyrophosphate dihydrate crystal deposits” |
| 725 | Chang et al. ^725^ | 2020 | Case report | Histology | - | “is a crystal arthropathy primarily affecting the peripheral joints, most commonly the wrist and knee” |
| 726 | Chernoff et al. ^726^ | 2020 | Case report | - | Deposition | - |
| 727 | Chisari et al. ^727^ | 2020 | Cohort study retrospective | SFA | Disease | - |
| 728 | Choi et al. ^728^ | 2020 | Case report | Histology | Deposition | “is a crystal arthropathy” |
| 729 | Ciaffi et al. ^729^ | 2020 | Case report | SFA | Disease | - |
| 730 | Cipolletta et al. ^730^ | 2020 | Case-control study prospective | McCarthy criteria | - | “is characterized by the deposition of calcium pyrophosphate crystals within articular and periarticular structures” |
| 731 | Cipolletta et al. ^731^ | 2020 | Systematic literature review | McCarthy criteria | Deposition | “is characterized by the deposition of calcium pyrophosphate crystals at articular and periarticular level” |
| 732 | Cipolletta et al. ^732^ | 2020 | Case report | SFA & Imaging | Deposition | “is a common arthropathy caused by the deposition of calcium pyrophosphate crystals within articular and periarticular tissues” |
| 733 | Collinot et al. ^733^ | 2020 | Case report | SFA | Deposition | - |
| 734 | Conticini et al. ^734^ | 2020 | Case report | Imaging | Disease | - |
| 735 | Conticini et al. ^735^ | 2020 | Case report | Imaging | - | - |
| 736 | Zimmer et al. ^736^ | 2020 | Case report | Imaging | Disease | - |
| 737 | Slouma et al. ^737^ | 2020 | Case report | SFA | Deposition | “typically targets the articular structures periarticular involvement including the tendons and bursae” |
| 738 | Ziegeler et al. ^738^ | 2020 | Case-control study retrospective | McCarthy | Disease | - |
| 739 | Lu et al. ^739^ | 2020 | Cohort study retrospective | Imaging | Deposition | “is a spectrum of diseases in which CPPD crystal deposited in joint cartilage, ligaments, tendons, bursae, and joint capsules” |
| 740 | Nguyen et al. ^740^ | 2020 | Cohort study cross-sectional | Imaging | Deposition | - |
| 741 | Kenny et al. ^741^ | 2020 | Case report | Expert opinion | Deposition | “is a disease of the elderly and its prevalence doubles every decade over 60” |
| 742 | Miura et al. ^742^ | 2020 | Case report | Imaging | Deposition | - |
| 743 | Latourte et al. ^743^ | 2020 | Cohort study longitudinal | SFA | Deposition | “is a prevalent condition characterized by the presence of CPP crystals in articular tissues, especially hyaline cartilage and fibrocartilage” |
| 744 | Oduyale et al. ^744^ | 2020 | Case report | SFA | Deposition | - |
| 745 | De Silva et al. ^745^ | 2020 | Case report | Imaging | - | - |
| 746 | Singla Amit et al. ^746^ | 2020 | Scoping review | SFA & Histology | Deposition | - |
| 747 | Hotokezaka et al. ^747^ | 2020 | Case report | Histology | - | “is a rare, benign crystalline arthropathy, first reported by McCarty *et al*.” |
| 748 | He et al. ^748^ | 2020 | Cohort study cross-sectional | Imaging | - | “is a disease characterized by the formation of calcium pyrophosphate and basic calcium phosphate crystals in the pericellular matrix of cartilage, and by the calcification of articular fibrocartilage and hyaline cartilage” |
| 749 | Hage et al. ^749^ | 2020 | Case-control study prospective | Expert opinion | - | “is the most frequent inflammatory arthritis in hemodyalisis patients” |
| 750 | Greca et al. ^750^ | 2020 | Case report | Histology | Deposition | - |
| 751 | Guermazi et al. ^751^ | 2020 | Cohort study cross-sectional | - | - | - |
| 752 | Simon et al. ^752^ | 2020 | Scoping review | - | - | - |
| 753 | Haikal et al. ^753^ | 2020 | Cohort study retrospective | Expert opinion | - | - |
| 754 | Gomez et al. ^754^ | 2020 | Case report | Histology | - | - |
| 755 | Gandikota et al. ^755^ | 2020 | Scoping review | - | - | - |
| 756 | Foreman et al. ^756^ | 2020 | Cohort study longitudinal | Imaging | - | “is an arthropathy defined as the presence of calcium-containing crystal depositions on radiographs in cartilage or other soft-tissue structures of the joint” |
| 757 | Forlizzi et al. ^757^ | 2020 | Case report | SFA | - | “is a subtype of crystalline arthropathy involving deposition of calcium pyrophosphate dihydrate crystals and can present with an erythematous, hot, swollen mono-articular arthritis similar in manifestation to acute septic arthritis” |
| 758 | Filippou et al. ^758^ | 2020 | Scoping review | - | - | - |
| 759 | Filippou et al ^759^ | 2020 | Cohort study cross-sectional | Histology | Deposition | “is an umbrella term used to describe all instances of calcium pyrophosphate crystals occurrence in tissues” |
| 760 | Filippucci et al. ^760^ | 2020 | Scoping review | - | - | - |
| 761 | Falkowski et al. ^761^ | 2020 | Case-control study prospective | SFA | Disease | “is the umbrella term that includes acute calcium pyrophosphate crystal arthritis, osteoarthritis with CPPD and chronic CPP crystal inflammatory arthritis, calcium pyrophosphate deposition (CPPD) can cause arthropathies from deposition of calcium pyrophosphate crystals” |
| 762 | Dupré et al. ^762^ | 2020 | Cohort study retrospective | - | - | - |
| 763 | Pascart et al. ^763^ | 2020 | Cohort study retrospective | Imaging | - | - |
| 764 | Pascual et al. ^764^ | 2020 | Case report | - | Deposition | - |
| 765 | Pastor et al. ^765^ | 2020 | Cohort study longitudinal | SFA | - | - |
| 766 | Persons et al. ^766^ | 2020 | Scoping review | SFA | - | - |
| 767 | Salles et al. ^767^ | 2020 | Scoping review | - | Disease | - |
| 768 | Quin et al. ^768^ | 2020 | Systematic literature review | SFA | Deposition | “is a common crystalline arthropathy in which calcium pyrophosphate crystals are deposited into various tissues” |
| 769 | Ramonda et al. ^769^ | 2020 | Case report | Imaging | Deposition | - |
| 770 | Sugimoto et al. ^770^ | 2020 | Case report | Imaging | - | - |
| 771 | Sakellariou et al. ^771^ | 2020 | Systematic literature review | SFA & Imaging & Histology | Disease | - |
| 772 | Tang et al. ^772^ | 2020 | Case series | Imaging | - | - |
| 773 | Scarlini et al. ^773^ | 2020 | Case report | - | - | - |
| 774 | Tedeschi et al. ^774^ | 2020 | Letter to the editor | SFA | - | - |
| 775 | Thomas et al. ^775^ | 2020 | Case report | SFA | Deposition | - |
| 776 | Zell et al. ^776^ | 2020 | Cohort study cross-sectional | Synovial fluid analysis | Disease | “is arthritis due to deposition of calcium pyrophosphate crystals in synovial fluid or tissues, , which can cause signiﬁcant morbidity and is often under- diagnosed |
| 777 | Zavisanos et al. ^777^ | 2020 | Cohort study cross-sectional | SFA | - | - |
| 778 | Yates et al. ^778^ | 2020 | Cohort study retrospective | - | Disease | - |
| 779 | Vanni et al. ^779^ | 2020 | Case report | - | - | - |
| 780 | Willems et al. ^780^ | 2020 | Cohort study retrospective | Imaging | - | - |
| 781 | Willekens et al. ^781^ | 2020 | Cohort study retrospective | Imaging | - | - |
| 782 | Chotard et al. ^782^ | 2021 | Cohort study cross-sectional | Imaging | Deposition | - |
| 783 | Dang et al. ^783^ | 2021 | Case report | Histology | Deposition | “is a non-infectious arthropathy with deposition of calcium pyrophosphate dihydrate crystals in the intra- or peri-articular structures and is often seen in the elderly particularly involving the knee joint” |
| 784 | Kravchenko et al. ^784^ | 2021 | Case-control study retrospective | Expert opinion | Deposition | “is thought to occur when an imbalance in inorganic pyrophosphate production and pyrophosphatase leads to saturation and precipitation of CPP crystals near the surface of cartilage” |
| 785 | Mank et al. ^785^ | 2021 | Case report | Imaging | Disease | “is a crystalline arthropathy seen in middle age to elderly patients, common inflammatory joint disease” |
| 786 | Kamalaksha et al. ^786^ | 2021 | Case report | Imaging | Deposition | - |
| 787 | Qin et al. ^787^ | 2021 | Case series | Imaging | Deposition | - |
| 788 | Altomare et al. ^788^ | 2021 | Scoping review | SFA & Imaging | Deposition | “is characterized by the deposition of calcium pyrophosphate crystals in the cartilage, that can manifest in most cases as a subclinical condition named chondrocalcinosis [...] and in other cases deposition can cause flares of arthritis known as acute CPP crystal arthritis” |
| 789 | Aoki et al. ^789^ | 2021 | Case report | - | Deposition | - |
| 790 | Mazzoni et al. ^790^ | 2021 | Case report | Histology | Disease | - |
| 791 | Bamgboje et al. ^791^ | 2021 | Case report | Imaging | Deposition | - |
| 792 | Barge et al. ^792^ | 2021 | Case report | Imaging | - | - |
| 793 | Bashir et al. ^793^ | 2021 | Cohort study retrospective | - | Deposition | “is a common crystalline arthritis with a variety of manifestations including acute and chronic inflammatory arthritis as well as osteoarthritis in unusual joints” |
| 794 | Liao et al. ^794^ | 2021 | Case report | Histology | Deposition | - |
| 795 | Bernabei et al. ^795^ | 2021 | Case report | Histology | - | - |
| 796 | Maheswaranathan et al. ^796^ | 2021 | Case report | Histology | Deposition | - |
| 797 | Brotherton et al. ^797^ | 2021 | Case report | SFA | Disease | - |
| 798 | Budzik et al. ^798^ | 2021 | Case-control study prospective | SFA | - | - |
| 799 | Mizumoto et al. ^799^ | 2021 | Case report | Imaging & Histology | Deposition | - |
| 800 | Cai et al. ^800^ | 2021 | Scoping review | - | Deposition | “is an umbrella term that includes acute CPP crystal arthritis (also known as "pseudogout") - chronic CPP crystal inflammatory arthritis and CPPD with osteoarthritis” |
| 801 | Cai et al. ^801^ | 2021 | Cohort study cross-sectional | - | Deposition | - |
| 802 | Sirotti et al. ^802^ | 2021 | Cohort study cross-sectional | Histology | Deposition | “is an umbrella term used to describe all occurrences of calcium pyrophosphate crystals in tissues” |
| 803 | Cenin et al. ^803^ | 2021 | Cohort study retrospective | SFA & Imaging | Disease | - |
| 804 | Chakravorty et al. ^804^ | 2021 | Case report | Histology | Deposition | “is a crystalline arthropathy that usually affects large joints and periarticular tissue” |
| 805 | Cho et al. ^805^ | 2021 | Case report | SFA | - | - |
| 806 | Cipolletta et al. ^806^ | 2021 | Meta-analysis | - | Deposition | “is a crystal arthropathy characterized by the deposition of calcium pyrophosphate crystals in articular and periarticular structures” |
| 807 | Doaré et al. ^807^ | 2021 | Cohort study retrospective | - | Disease | - |
| 808 | Rammanohar et al. ^808^ | 2021 | Case report | Imaging | Disease | - |
| 809 | Lo et al. ^809^ | 2021 | Case report | Histology | Deposition | - |
| 810 | Slouma et al. ^810^ | 2021 | Letter to the editor | Imaging | Disease | - |
| 811 | Ziegeler et al. ^811^ | 2021 | Case-control study retrospective | McCarthy criteria | Disease | - |
| 812 | Cudrici et al. ^812^ | 2021 | Cohort study longitudinal | SFA & Histology | Disease | “is a pro-calcifying systemic disorder” |
| 813 | Lu et al. ^813^ | 2021 | Case series | Histology | Deposition | - |
| 814 | Sang T. Kim et al. ^814^ | 2021 | Case report | SFA | Disease | “is an autoinflammatory disorder” |
| 815 | Dermawan et al. ^815^ | 2021 | Cohort study retrospective | - | Disease | “is a metabolic arthritis produced by the deposition of calcium pyrophosphate dihydrate crystals in the synovium, joint capsule, and periarticular soft tissues, including synovial fluid, of large joints and digits” |
| 816 | Okamoto et al. ^816^ | 2021 | Case report | Imaging | Deposition | - |
| 817 | Hill et al. ^817^ | 2021 | Case report | - | - | “can lead to debilitating arthritis” |
| 818 | Ham et al. ^818^ | 2021 | Scoping review | - | - | - |
| 819 | Grunz et al. ^819^ | 2021 | Scoping review | - | - | - |
| 820 | Haller et al. ^820^ | 2021 | Case report | Imaging | - | - |
| 821 | Germann et al. ^821^ | 2021 | Case-control study prospective | Imaging | Deposition | “is a frequent cause of inflammation and pain known as pseudo-gout, commonly affecting the knee joint, followed by the wrist” |
| 822 | Gewolb et al. ^822^ | 2021 | Case report | Histology | Disease | “is a disease of crystal deposition of unknown etiology” |
| 823 | Goldman et al. ^823^ | 2021 | Case report | Imaging | - | - |
| 824 | Grassi et al. ^824^ | 2021 | Case report | Imaging | - | - |
| 825 | Fuller et al. ^825^ | 2021 | Scoping review | - | Deposition | “is a common form of inflammatory arthritis that manifests as acute CPP crystal arthritis, chronic CPP crystal inflammatory arthritis, and CPPD + osteoarthritis” |
| 826 | Fuller et al. ^826^ | 2021 | Scoping review | - | Deposition | “manifests as acute CPP crystal arthritis, chronic CPP crystal inflammatory arthritis, and osteoarthritis (OA) with CPPD, and is commonly detected as articular chondrocalcinosis on radiographs” |
| 827 | Finkelstein et al. ^827^ | 2021 | Scoping review | - | - | “is a crystalline arthropathy that may cause acute joint pain, although the pathogenic mechanism of leading to its accumulation is not well understood” |
| 828 | Fitzgerald et al. ^828^ | 2021 | Scoping review | - | Deposition | - |
| 829 | Filippou et al. ^829^ | 2021 | Scoping review | - | Deposition | - |
| 830 | Filippou et al. ^830^ | 2021 | Case-control study prospective | SFA | Deposition | - |
| 831 | Falsetti et al. ^831^ | 2021 | Case-control study prospective | - | - | - |
| 832 | Ea et al. ^832^ | 2021 | Cohort study cross-sectional | Histology | - | - |
| 833 | Dumusc et al. ^833^ | 2021 | Letter to the editor | - | - | - |
| 834 | Dumusc et al. ^834^ | 2021 | Randomized controlled trial | SFA | - | - |
| 835 | Parperis et al. ^835^ | 2021 | Systematic literature review | SFA | Deposition | “Is a common cause of acute and chronic arthritis”; “is a common form of crystal-induced arthropathy characterized by the deposition of calcium pyrophosphate crystals in joints and soft tissues resulting in inflammation and joint damage” |
| 836 | Shiraishi et al. ^836^ | 2021 | Case report | SFA | Disease | - |
| 837 | Pongmanee et al. ^837^ | 2021 | Case report | Histology | Deposition | - |
| 838 | Schwabl et al. ^838^ | 2021 | Case report | - | Disease | - |
| 839 | Schroeder et al. ^839^ | 2021 | Case series | SFA | - | - |
| 840 | Qureshi et al. ^840^ | 2021 | Case report | SFA | - | - |
| 841 | Stack et al. ^841^ | 2021 | Scoping review | - | Deposition | - |
| 842 | Stücker et al. ^842^ | 2021 | Scoping review | - | Deposition | - |
| 843 | Sullivan et al. ^843^ | 2021 | Scoping review | Imaging | - | “is characterized by deposition of calcium pyrophosphate crystals in articular tissue, and has a heterogenous clinical presentation” |
| 844 | Tagoe et al. ^844^ | 2021 | Cohort study cross-sectional | Imaging | - | - |
| 845 | Tang et al. ^845^ | 2021 | Case series | Imaging | Deposition | - |
| 846 | Tedeschi et al. ^846^ | 2021 | Cohort study retrospective | SFA | - | - |
| 847 | Tedeschi et al. ^847^ | 2021 | Systematic literature review | - | - | - |
| 848 | Tedeschi et al. ^848^ | 2021 | Case-control study prospective | SFA | - | - |
| 849 | Rosenthal et al. ^849^ | 2021 | Scoping review | SFA | Deposition | “is associated with advanced age, and is rare in people under age 60” |
| 850 | Zamudio-Cuevas et al. ^850^ | 2021 | Cohort study retrospective | SFA | Deposition | - |
| 851 | Yurube et al. ^851^ | 2021 | Case report | Imaging | Deposition | - |
| 852 | Williams et al. ^852^ | 2021 | Systematic literature review | - | Disease | “is a disease caused by calcium pyrophosphate crystals” |
| 853 | Jeong et al. ^853^ | 2022 | Case report | Imaging | Disease | - |
| 854 | Abdelkefi et al. ^854^ | 2022 | Case report | Imaging | - | - |
| 855 | Huang et al. ^855^ | 2022 | Case report | - | Disease | - |
| 856 | Lee et al. ^856^ | 2022 | Case report | SFA & Imaging | Deposition | - |
| 857 | Bougioukas et al. ^857^ | 2022 | Case report | - | - | - |
| 858 | Bustamante et al. ^858^ | 2022 | Cohort study cross-sectional | SFA | - | - |
| 859 | Cadiou et al. ^859^ | 2022 | Case report | SFA & Imaging | Deposition | “is a common cause of rheumatological symptoms: from an acute form which is typically revealed by arthritis of specific joints to chronic form with polyarticular involvement mimicking other rheumatologic conditions and with osteoarthritis” |
| 860 | Perozzo et al. ^860^ | 2022 | Case report | SFA | Deposition | “includes a variety of clinical syndromes, including acute calcium pyrophosphate crystal arthritis” |
| 861 | Krekeler et al. ^861^ | 2022 | Cohort study retrospective | Expert opinion | Deposition | “is a rather common but not well studied disease to date, may present as an acute arthritis or a chronic arthropathy with development of radiographic structural changes, or may present as an often incidental radiographic finding called chondrocalcinosis” |
| 862 | Cipolletta et al. ^862^ | 2022 | Cohort study cross-sectional | SFA & Imaging | Deposition | - |
| 863 | Cipolletta et al. ^863^ | 2022 | Case-control study prospective | - | Deposition | “is a crystal arthropathy caused by the deposition of calcium pyrophosphate crystals within articular and periarticular structures” |
| 864 | Liew et al. ^864^ | 2022 | Case-control study prospective | Expert opinion | Deposition | - |
| 865 | Nogueira Gomes et al. ^865^ | 2022 | Case report | Imaging | Deposition | - |
| 866 | Muller et al. ^866^ | 2022 | Case report | Imaging | Deposition | - |
| 867 | Ohyama et al. ^867^ | 2022 | Case report | Imaging | Deposition | - |
| 868 | Parmar et al. ^868^ | 2022 | Systematic literature review | - | - | - |
| 869 | Haas et al. ^869^ | 2022 | Case report | Histology | Deposition | - |
| 870 | Geneva-Popova et al. ^870^ | 2022 | Case-control study retrospective | SFA | - | - |
| 871 | Francia et al. ^871^ | 2022 | Case report | SFA | Disease | - |
| 872 | Drosos et al. ^872^ | 2022 | Case report | Histology | - | “is a term that comprises many clinical disorders including CPP crystal arthritis” |
| 873 | Parperis et al. ^873^ | 2022 | Cohort study cross-sectional | SFA | Deposition | “is a common crystal‐induced arthropathy characterized by the deposition of calcium pyrophosphate crystals in the articular and periarticular tissues that might lead to inflammatory arthritis joint damage impairment and disability” |
| 874 | Shams et al. ^874^ | 2022 | Case report | Imaging | Disease | - |
| 875 | Pfeil et al. ^875^ | 2022 | Scoping review | Imaging | Deposition | - |
| 876 | Reijnierse et al. ^876^ | 2022 | Systematic literature review | Imaging | - | - |
| 877 | Takeda et al. ^877^ | 2022 | Case report | Histology | Deposition | - |
| 878 | Tantillo et al. ^878^ | 2022 | Case report | Histology | - | “is a common inflammatory arthropathy found in individuals over the age of 60” |
| 879 | Tedeschi et al. ^879^ | 2022 | Scoping review | - | - | - |
| 880 | Tedeschi et al. ^880^ | 2022 | Cohort study retrospective | - | - | “is a common crystalline arthritis” |
| 881 | Terauchi et al. ^881^ | 2022 | Case report | - | Deposition | “is a rare benign crystalline arthropathy” |
| 882 | Zeng et al. ^882^ | 2022 | Case report | Imaging | - | - |
| 883 | Zelano et al. ^883^ | 2022 | Scoping review | Imaging | Deposition | - |
| 884 | Yamauchi et al. ^884^ | 2022 | Case report | Imaging | Disease | - |
| 885 | Wireko et al. ^885^ | 2022 | Case report | SFA & Imaging | Disease | - |
| 886 | Weaver et al. ^886^ | 2022 | Systematic literature review | Imaging | Disease | “is a metabolic arthropathy secondary to deposition of calcium pyrophosphate dihydrate crystals in the articular or periarticular tissues” |

*^a^: CPPD disease designating the deposition (both symptomatic and asymptomatic) or the disease (only symptomatic); SFA : synovial fluid analysis*

References

1. Kakitsubata Y, Boutin RD, Theodorou DJ, et al. Calcium pyrophosphate dihydrate crystal deposition in and around the atlantoaxial joint: association with type 2 odontoid fractures in nine patients. *Radiology* 2000; 216: 213–219.

2. Agudelo CA, Wise CM. Crystal-associated arthritis in the elderly. *Rheum Dis Clin North Am* 2000; 26: 527–546, vii.

3. Aoyama S, Kino K, Amagasa T, et al. Differential diagnosis of calcium pyrophosphate dihydrate deposition of the temporomandibular joint. *Br J Oral Maxillofac Surg* 2000; 38: 550–553.

4. Baysal T, Baysal O, Kutlu R, et al. The crowned dens syndrome: a rare form of calcium pyrophosphate deposition disease. *Eur Radiol* 2000; 10: 1003–1005.

5. Calò L, Punzi L, Semplicini A. Hypomagnesemia and chondrocalcinosis in Bartter’s and Gitelman’s syndrome: review of the pathogenetic mechanisms. *Am J Nephrol* 2000; 20: 347–350.

6. Caramaschi P, Biasi D, Carletto A, et al. Calcium pyrophosphate dihydrate crystal deposition disease and primary hyperparathyroidism associated with rheumatoid arthritis: description of 3 cases. *Clin Exp Rheumatol* 2000; 18: 110.

7. McGill NW. Gout and other crystal-associated arthropathies. *Baillieres Best Pract Res Clin Rheumatol* 2000; 14: 445–460.

8. Cheung HS. Calcium crystal effects on the cells of the joint: implications for pathogenesis of disease. *Curr Opin Rheumatol* 2000; 12: 223–227.

9. Cibere J. Rheumatology: 4. Acute monoarthritis. *CMAJ Can Med Assoc J J Assoc Medicale Can* 2000; 162: 1577–1583.

10. Lomoschitz F, Krestan C, Sulzbacher I, et al. Quiz case: CPPD crystal deposition disease. *Eur J Radiol* 2000; 35: 78–80.

11. Kroesen S, Schmid W, Theiler R. Induction of an acute attack of calcium pyrophosphate dihydrate arthritis by intra-articular injection of hylan G-F 20 (Synvisc). *Clin Rheumatol* 2000; 19: 147–149.

12. Li-Yu J, Schumacher HRJ, Gratwick G. Invasive tophaceous pseudogout in the temporomandibular joint: misdiagnosis as tumor: case report and review of the literature. *J Clin Rheumatol Pract Rep Rheum Musculoskelet Dis* 2000; 6: 272–277.

13. Mostafapour SP, Futran ND. Tumors and tumorous masses presenting as temporomandibular joint syndrome. *Otolaryngol--Head Neck Surg Off J Am Acad Otolaryngol-Head Neck Surg* 2000; 123: 459–464.

14. Oostveen JC, van de Laar MA. Magnetic resonance imaging in rheumatic disorders of the spine and sacroiliac joints. *Semin Arthritis Rheum* 2000; 30: 52–69.

15. Hasegawa H, Nakajima Y, Mabuchi E, et al. Retro-odontoid massive calcium pyrophosphate crystal deposition--case report. *Neurol Med Chir (Tokyo)* 2000; 40: 387–390.

16. Punzi L, Pianon M, Piero SG, et al. Pseudogout and intraarticular hyaluronate injections: comment on the article by Disla et al. *Arthritis Rheum* 2000; 43: 1660–1661.

17. Steinbach LS, Resnick D. Calcium pyrophosphate dihydrate crystal deposition disease: imaging perspectives. *Curr Probl Diagn Radiol* 2000; 29: 209–229.

18. Sagarin MJ. Pseudogout. *J Emerg Med* 2000; 18: 373–374.

19. Tedd RJ, Norton MR, Thomas WG. Bilateral simultaneous atraumatic quadriceps tendon ruptures associated with ‘pseudogout’. *Injury* 2000; 31: 467–469.

20. Teramoto S. Possible causes of acute pseudogout in older patients with osteoarthritis. *Am J Med* 2000; 109: 75–76.

21. Usuki K, Yamashita M, Kanzaki T. A case of pseudogout. *J Dermatol* 2000; 27: 284–287.

22. Yamagami T, Kawano N, Nakano H. Calcification of the cervical ligamentum flavum--case report. *Neurol Med Chir (Tokyo)* 2000; 40: 234–238.

23. Wong PK, York JR, Ryan D, et al. Crystal unclear. *Ann Rheum Dis* 2000; 59: 83–85.

24. Malaviya AN, Al-Shari IM, Al-Shayeb AR, et al. Calcium pyrophosphatase dihydrate (CPPD) crystal deposition disease in a teaching hospital in Kuwait. *Ann Rheum Dis* 2001; 60: 416–419.

25. Aoyama T, Takahashi S. Cubital tunnel syndrome caused by tumoral deposition of calcium pyrophosphate dihydrate crystals: a case report. *J Shoulder Elbow Surg* 2001; 10: 194–196.

26. Assaker R, Louis E, Boutry N, et al. Foramen magnum syndrome secondary to calcium pyrophosphate crystal deposition in the transverse ligament of the atlas. *Spine* 2001; 26: 1396–1400.

27. Bernardeau C, Bucki B, Lioté F. Acute arthritis after intra-articular hyaluronate injection: onset of effusions without crystal. *Ann Rheum Dis* 2001; 60: 518–520.

28. Mula M, Bordin G, Naldi P, et al. Crowned dens syndrome in an elderly man. *Neurology* 2001; 56: 275.

29. Cabre P, Pascal-Moussellard H, Kaidomar S, et al. Six cases of cervical ligamentum flavum calcification in Blacks in the French West Indies. *Joint Bone Spine* 2001; 68: 158–165.

30. Maldonado I, Reginato AM, Reginato AJ. Familial calcium crystal diseases: what have we learned? *Curr Opin Rheumatol* 2001; 13: 225–233.

31. Canhão H, Fonseca JE, Leandro MJ, et al. Cross-sectional study of 50 patients with calcium pyrophosphate dihydrate crystal arthropathy. *Clin Rheumatol* 2001; 20: 119–122.

32. Caspi D, Flusser G, Farber I, et al. Clinical, radiologic, demographic, and occupational aspects of hand osteoarthritis in the elderly. *Semin Arthritis Rheum* 2001; 30: 321–331.

33. Kelley JT 3rd, Agudelo CA, Sharma V, et al. Fever with acute arthritis in calcium pyrophosphate dihydrate deposition disease: a missed explanation for altered mental status in elderly patients? *J Clin Rheumatol Pract Rep Rheum Musculoskelet Dis* 2001; 7: 322–325.

34. Chivukula M, Shidham G, Shidham V. Pathologic quiz case. Crystal deposition disease of the knee joint. *Arch Pathol Lab Med* 2001; 125: 705–706.

35. Coombs RJ, Padanilam TG, Phillips E. Tumoral pseudogout of the metatarsal. *Skeletal Radiol* 2002; 31: 39–42.

36. Olin HB, Pedersen K, Francis D, et al. A very rare benign tumour in the parotid region: calcium pyrophosphate dihydrate crystal deposition disease. *J Laryngol Otol* 2001; 115: 504–506.

37. Lambrecht N, Nelson SD, Seeger L, et al. Tophaceous pseudogout: a pitfall in the diagnosis of chondrosarcoma. *Diagn Cytopathol* 2001; 25: 258–261.

38. Matsukado K, Amano T, Itou O, et al. Tumoral calcinosis in the upper cervical spine causing progressive radiculomyelopathy--case report. *Neurol Med Chir (Tokyo)* 2001; 41: 411–414.

39. Ohira T, Ishikawa K. Preservation of calcium pyrophosphate dihydrate crystals: effect of Mayer’s haematoxylin staining period. *Ann Rheum Dis* 2001; 60: 80–82.

40. Halverson PB, Derfus BA. Calcium crystal-induced inflammation. *Curr Opin Rheumatol* 2001; 13: 221–224.

41. Eriksson L, Mertens F, Akerman M, et al. Calcium pyrophosphate dihydrate crystal deposition disease in the temporomandibular joint: diagnostic difficulties and clonal chromosome aberrations in a case followed up for 5 years. *J Oral Maxillofac Surg Off J Am Assoc Oral Maxillofac Surg* 2001; 59: 1217–1220.

42. Shidham V, Chivukula M, Basir Z, et al. Evaluation of crystals in formalin-fixed, paraffin-embedded tissue sections for the differential diagnosis of pseudogout, gout, and tumoral calcinosis. *Mod Pathol Off J U S Can Acad Pathol Inc* 2001; 14: 806–810.

43. Perez-Ruiz F, Testillano M, Gastaca MA, et al. ‘Pseudoseptic’ pseudogout associated with hypomagnesemia in liver transplant patients. *Transplantation* 2001; 71: 696–698.

44. Peter A, Simmen BR, Brühlmann P, et al. Osteoarthritis of the scaphoidtrapezium joint: an early sign of calcium pyrophosphate dihydrate disease. *Clin Rheumatol* 2001; 20: 20–24.

45. Selvi E, Manganelli S, Catenaccio M, et al. Diff Quik staining method for detection and identification of monosodium urate and calcium pyrophosphate crystals in synovial fluids. *Ann Rheum Dis* 2001; 60: 194–198.

46. Reuge L, Van Linthoudt D, Gerster JC. Local deposition of calcium pyrophosphate crystals in evolution of knee osteoarthritis. *Clin Rheumatol* 2001; 20: 428–431.

47. Rosenthal AK, Mandel N. Identification of crystals in synovial fluids and joint tissues. *Curr Rheumatol Rep* 2001; 3: 11–16.

48. Yamakawa K, Iwasaki H, Ohjimi Y, et al. Tumoral calcium pyrophosphate dihydrate crystal deposition disease. A clinicopathologic analysis of five cases. *Pathol Res Pract* 2001; 197: 499–506.

49. van den Bosch F, Baeten D, Kruithof E, et al. Characteristic marco- and microscopic aspect of the synovial membrane in crystal induced arthritis. *J Rheumatol* 2001; 28: 392–393.

50. Wendling D, Jeannin-Louys L, Kremer P, et al. Adult hypophosphatasia. Current aspects. *Joint Bone Spine* 2001; 68: 120–124.

51. Jaovisidha K, Rosenthal AK. Calcium crystals in osteoarthritis. *Curr Opin Rheumatol* 2002; 14: 298–302.

52. Al-Arfaj AS. The relationship between chondrocalcinosis and osteoarthritis in Saudi Arabia. *Clin Rheumatol* 2002; 21: 493–496.

53. Al-Arfaj AS, Al-Boukai AA. Articular chondrocalcinosis in Saudi Arabia. *Saudi Med J* 2002; 23: 577–579.

54. Biankin S, Jaworski R, Mawad S. Tumoural calcium pyrophosphate dihydrate crystal deposition disease presenting clinically as a malignant soft tissue mass diagnosed on fine needle aspiration biopsy. *Pathology (Phila)* 2002; 34: 336–338.

55. Song JS, Lee YH, Kim SS, et al. A case of calcium pyrophosphate dihydrate crystal deposition disease presenting as an acute polyarthritis. *J Korean Med Sci* 2002; 17: 423–425.

56. Ziswiler M, Radü E-W, Romero J. Chondrocalcinosis in an isolated suprapatellar pouch with recurrent effusion. *Arthrosc J Arthrosc Relat Surg Off Publ Arthrosc Assoc N Am Int Arthrosc Assoc* 2002; 18: E14.

57. Cottrell DA, Nierzwicki BL, Jacob GA, et al. Nontender mass in the parotid region. *J Oral Maxillofac Surg Off J Am Assoc Oral Maxillofac Surg* 2002; 60: 912–917.

58. Kobayashi H, Akizuki S, Takizawa T, et al. Three cases of pseudogout complicated with unicondylar knee arthroplasty. *Arch Orthop Trauma Surg* 2002; 122: 469–471.

59. Derfus BA, Kurian JB, Butler JJ, et al. The high prevalence of pathologic calcium crystals in pre-operative knees. *J Rheumatol* 2002; 29: 570–574.

60. Pakzad K, Yang YJ, Ambrose JL, et al. Diagnosis of calcium pyrophosphate dihydrate deposition disease by fine needle aspiration biopsy: a case report. *Acta Cytol* 2002; 46: 46–49.

61. Sofka CM, Adler RS, Cordasco FA. Ultrasound diagnosis of chondrocalcinosis in the knee. *Skeletal Radiol* 2002; 31: 43–45.

62. Houdaille L, Prévot G, Ripault H, et al. Miliary tuberculosis with crystal deposition disease leading to a diagnosis of tuberculous arthritis. *Joint Bone Spine* 2002; 69: 338–340.

63. Hayashi M, Matsunaga T, Tanikawa H. Idiopathic widespread calcium pyrophosphate dihydrate crystal deposition disease in a young patient. *Skeletal Radiol* 2002; 31: 246–250.

64. Greaves S, Fordyce A. Bilateral temporomandibular joint pseudogout. *Br Dent J* 2002; 192: 25–27.

65. Guggi V, Calame L, Gerster J-C. Contribution of digit joint aspiration to the diagnosis of rheumatic diseases. *Joint Bone Spine* 2002; 69: 58–61.

66. Gadgil AA, Eisenstein SM, Darby A, et al. Bilateral symptomatic synovial cysts of the lumbar spine caused by calcium pyrophosphate deposition disease: a case report. *Spine* 2002; 27: E428-431.

67. Gálvez J, Sáiz E, Linares LF, et al. Delayed examination of synovial fluid by ordinary and polarised light microscopy to detect and identify crystals. *Ann Rheum Dis* 2002; 61: 444–447.

68. Foldes K. Knee chondrocalcinosis: an ultrasonographic study of the hyalin cartilage. *Clin Imaging* 2002; 26: 194–196.

69. Dodd LG, Major NM. Fine-needle aspiration cytology of articular and periarticular lesions. *Cancer* 2002; 96: 157–165.

70. Santos-Ocampo AS, Tupasi TE, Villanueva F, et al. Mycobacterium tuberculosis infection of a tophaceous pseudogout nodule. *J Rheumatol* 2002; 29: 1093–1096.

71. Swan A, Amer H, Dieppe P. The value of synovial fluid assays in the diagnosis of joint disease: a literature survey. *Ann Rheum Dis* 2002; 61: 493–498.

72. Romera M. Effect of intraarticular hyaluronate injections in chondrocalcinosis: comment on the article by Martens. *Arthritis Rheum* 2002; 46: 847; author reply 847.

73. Terkeltaub RA. What does cartilage calcification tell us about osteoarthritis? *J Rheumatol* 2002; 29: 411–415.

74. Terkeltaub R. Pseudogout, hypomagnesemia, and liver transplantation. *Curr Rheumatol Rep* 2002; 4: 243–244.

75. Rubin MR, Silverberg SJ. Rheumatic manifestations of primary hyperparathyroidism and parathyroid hormone therapy. *Curr Rheumatol Rep* 2002; 4: 179–185.

76. Waguri-Nagaya Y, Kubota Y, Sekiya I, et al. Extensor tendon rupture related to calcium pyrophosphate crystal deposition disease. *Rheumatol Int* 2002; 21: 243–246.

77. Jackson JL, O’Malley PG, Kroenke K. Evaluation of acute knee pain in primary care. *Ann Intern Med* 2003; 139: 575–588.

78. Ahn JK, Kim HJ, Kim EH, et al. Idiopathic calcium pyrophosphate dihydrate (CPPD) crystal deposition disease in a young male patient: a case report. *J Korean Med Sci* 2003; 18: 917–920.

79. Baty V, Prost B, Jouvet A, et al. Acute spinal cord compression and calcium pyrophosphate deposition disease. Case illustration. *J Neurosurg* 2003; 99: 240.

80. Bencardino JT, Hassankhani A. Calcium pyrophosphate dihydrate crystal deposition disease. *Semin Musculoskelet Radiol* 2003; 7: 175–185.

81. Brandt KD. Chondrocalcinosis, osteophytes and osteoarthritis. *Chondrocalcinosis Osteophytes Osteoarthr* 2003; 62: 499–500.

82. Cañete JD, Rodríguez JR, Salvador G, et al. Diagnostic usefulness of synovial vascular morphology in chronic arthritis. A systematic survey of 100 cases. *Semin Arthritis Rheum* 2003; 32: 378–387.

83. Caramaschi P, Biasi D, Villa G, et al. Clinical images: Neuropathic joint in a patient with calcium pyrophosphate deposition disease. *Arthritis Rheum* 2003; 48: 1170.

84. Neame RL, Carr AJ, Muir K, et al. UK community prevalence of knee chondrocalcinosis: evidence that correlation with osteoarthritis is through a shared association with osteophyte. *Ann Rheum Dis* 2003; 62: 513–518.

85. Nalbant S, Martinez JAM, Kitumnuaypong T, et al. Synovial fluid features and their relations to osteoarthritis severity: new findings from sequential studies. *Osteoarthritis Cartilage* 2003; 11: 50–54.

86. Muthukumar N, Karuppaswamy U. Tumoral calcium pyrophosphate dihydrate deposition disease of the ligamentum flavum. *Neurosurgery* 2003; 53: 103–8; discussion 108-109.

87. Ofluoğlu D, Gündüz OH, Ozaras N, et al. Early-onset hemochromatic arthropathy in a patient with idiopathic hypermobility syndrome. *Rheumatol Int* 2003; 23: 305–308.

88. Osano H, Matsumoto K, Kusama M. Calcium pyrophosphate dihydrate arthropathy with condylar destruction of the temporomandibular joint. *J Oral Sci* 2003; 45: 223–226.

89. Ozolek JA, Chu CT. Pseudogout of the craniocervical junction. *Arch Pathol Lab Med* 2003; 127: 895.

90. Sofka CM, Ghelman B. Radiographic tools for assessment of pathologic cartilage calcification. *Curr Opin Rheumatol* 2003; 15: 296–301.

91. Havitçioğlu H, Tatari H, Baran O, et al. Calcium pyrophosphate dihydrate crystal deposition disease mimicking malignant soft tissue tumor. *Knee Surg Sports Traumatol Arthrosc Off J ESSKA* 2003; 11: 263–266.

92. Hamburger MI, Lakhanpal S, Mooar PA, et al. Intra-articular hyaluronans: a review of product-specific safety profiles. *Semin Arthritis Rheum* 2003; 32: 296–309.

93. Halverson PB. Crystal deposition disease of the shoulder (including calcific tendonitis and milwaukee shoulder syndrome). *Curr Rheumatol Rep* 2003; 5: 244–247.

94. Pay S, Terkeltaub R. Calcium pyrophosphate dihydrate and hydroxyapatite crystal deposition in the joint: new developments relevant to the clinician. *Curr Rheumatol Rep* 2003; 5: 235–243.

95. Sallis JD, Cheung HS. Inhibitors of articular calcium crystal formation. *Curr Opin Rheumatol* 2003; 15: 321–325.

96. Rutsch F, Terkeltaub R. Parallels between arterial and cartilage calcification: what understanding artery calcification can teach us about chondrocalcinosis. *Curr Opin Rheumatol* 2003; 15: 302–310.

97. Yuan S, Bien C, Wener MH, et al. Repeat examination of synovial fluid for crystals: is it useful? *Clin Chem* 2003; 49: 1562–1563.

98. Williams CJ. Familial calcium pyrophosphate dihydrate deposition disease and the ANKH gene. *Curr Opin Rheumatol* 2003; 15: 326–331.

99. McKee S, Pendleton A, Dixey J, et al. Autosomal dominant early childhood seizures associated with chondrocalcinosis and a mutation in the ANKH Gene. *Epilepsia* 2004; 45: 1258–1260.

100. Abreu M, Johnson K, Chung CB, et al. Calcification in calcium pyrophosphate dihydrate (CPPD) crystalline deposits in the knee: anatomic, radiographic, MR imaging, and histologic study in cadavers. *Skeletal Radiol* 2004; 33: 392–398.

101. Aouba A, Vuillemin-Bodaghi V, Mutschler C, et al. Crowned dens syndrome misdiagnosed as polymyalgia rheumatica, giant cell arteritis, meningitis or spondylitis: an analysis of eight cases. *Rheumatol Oxf Engl* 2004; 43: 1508–1512.

102. Mader R. Calcium pyrophosphate dihydrate deposition disease of the wrist. *Clin Rheumatol* 2004; 23: 95–96.

103. Béjia I, Rtibi I, Touzi M, et al. Familial calcium pyrophosphate dihydrate deposition disease. A Tunisian kindred. *Joint Bone Spine* 2004; 71: 401–408.

104. Boutboul S, Bourcier T, Heligon J-P, et al. Familial pseudotumoral sclerochoroidal calcification associated with chondrocalcinosis. *Br J Ophthalmol* 2004; 88: 1094–1095.

105. Nordström DC, Aarnio M, Laasonen L. Acute pseudogout in a patient with treated acromegaly. *Scand J Rheumatol* 2004; 33: 443–445.

106. Chen LX, Clayburne G, Schumacher HR. Update on identification of pathogenic crystals in joint fluid. *Curr Rheumatol Rep* 2004; 6: 217–220.

107. Marsot-Dupuch K, Smoker WRK, Gentry LR, et al. Massive calcium pyrophosphate dihydrate crystal deposition disease: a cause of pain of the temporomandibular joint. *AJNR Am J Neuroradiol* 2004; 25: 876–879.

108. Netter P, Bardin T, Bianchi A, et al. The ANKH gene and familial calcium pyrophosphate dihydrate deposition disease. *Joint Bone Spine* 2004; 71: 365–368.

109. Griesdale DEGJ, Boyd M, Sahjpaul RL. Pseudogout of the transverse atlantal ligament: an unusual cause of cervical myelopathy. *Can J Neurol Sci J Can Sci Neurol* 2004; 31: 273–275.

110. Goldblatt F, Highton J, Kumara GR. Temporomandibular joint pseudogout: an uncommon site for a familiar condition. *Ann Rheum Dis* 2004; 63: 1706–1707.

111. Falsetti P, Frediani B, Acciai C, et al. Ultrasonographic study of Achilles tendon and plantar fascia in chondrocalcinosis. *J Rheumatol* 2004; 31: 2242–2250.

112. Ea H-K, Lioté F. Calcium pyrophosphate dihydrate and basic calcium phosphate crystal-induced arthropathies: update on pathogenesis, clinical features, and therapy. *Curr Rheumatol Rep* 2004; 6: 221–227.

113. Pasquetti P, Selvi E, Righeschi K, et al. Joint lavage and pseudogout. *Ann Rheum Dis* 2004; 63: 1529–1530.

114. Steinbach LS. Calcium pyrophosphate dihydrate and calcium hydroxyapatite crystal deposition diseases: imaging perspectives. *Calcium Pyrophosphate Dihydrate Calcium Hydroxyapatite Cryst Depos Dis Imaging Perspect* 2004; 42: 185–205, vii.

115. Saffar P. Chondrocalcinosis of the wrist. *J Hand Surg Edinb Scotl* 2004; 29: 486–493.

116. Sato Y, Yasuda T, Konno S, et al. Pseudogout showing meningoencephalitic symptoms: crowned dens syndrome. *Intern Med Tokyo Jpn* 2004; 43: 865–868.

117. Zhang W, Neame R, Doherty S, et al. Relative risk of knee chondrocalcinosis in siblings of index cases with pyrophosphate arthropathy. *Ann Rheum Dis* 2004; 63: 969–973.

118. Meul B, Ernestus K, Neugebauer J, et al. A case of chronic calcium pyrophosphate dihydrate crystal disease (tophaceous pseudogout) in the temporomandibular joint. *Oral Dis* 2005; 11: 113–115.

119. Mahmud T, Basu D, Dyson PHP. Crystal arthropathy of the lumbar spine: a series of six cases and a review of the literature. *J Bone Joint Surg Br* 2005; 87: 513–517.

120. McCarthy GM. Crystal deposition diseases: out of sight, out of mind. *Curr Opin Rheumatol* 2005; 17: 312–313.

121. Campo-Ruiz V, Patel D, Anderson RR, et al. Evaluation of human knee meniscus biopsies with near-infrared, reflectance confocal microscopy. A pilot study. *Int J Exp Pathol* 2005; 86: 297–307.

122. Srinivasan A, Belanger E, Woulfe J, et al. Calcium pyrophosphate dihydrate deposition disease resulting in cervical myelopathy. *Can J Neurol Sci J Can Sci Neurol* 2005; 32: 109–111.

123. Choy G. An update on the treatment options for gout and calcium pyrophosphate deposition. *Expert Opin Pharmacother* 2005; 6: 2443–2453.

124. Dalbeth N, Haskard DO. Inflammation and tissue damage in crystal deposition diseases. *Curr Opin Rheumatol* 2005; 17: 314–318.

125. Paolini S, Ciappetta P, Guiducci A, et al. Foraminal deposition of calcium pyrophosphate dihydrate crystals in the thoracic spine: possible relationship with disc herniation and implications for surgical planning. Report of two cases. *J Neurosurg Spine* 2005; 2: 75–78.

126. Papakonstantinou O, Mohana-Borges AVR, Campell L, et al. Hip arthropathy in a patient with primary hemochromatosis: MR imaging findings with pathologic correlation. *Skeletal Radiol* 2005; 34: 180–184.

127. Smolka W, Eggensperger N, Stauffer-Brauch EJ, et al. Calcium pyrophosphate dihydrate crystal deposition disease of the temporomandibular joint. *Oral Dis* 2005; 11: 104–108.

128. Holt G, Vass C, Kumar CS. Acute crystal arthritis mimicking infection after total knee arthroplasty. *BMJ* 2005; 331: 1322–1323.

129. Gupta R, Hu V, Reynolds T, et al. Sclerochoroidal calcification associated with Gitelman syndrome and calcium pyrophosphate dihydrate deposition. *J Clin Pathol* 2005; 58: 1334–1335.

130. Frediani B, Filippou G, Falsetti P, et al. Diagnosis of calcium pyrophosphate dihydrate crystal deposition disease: ultrasonographic criteria proposed. *Ann Rheum Dis* 2005; 64: 638–640.

131. Ea H-K, Blanchard A, Dougados M, et al. Chondrocalcinosis secondary to hypomagnesemia in Gitelman’s syndrome. *J Rheumatol* 2005; 32: 1840–1842.

132. Pascual E, Jovaní V. Synovial fluid analysis. *Best Pract Res Clin Rheumatol* 2005; 19: 371–386.

133. Shih VC, Kamen LB, Kaplan RJ, et al. Rehabilitation of orthopedic and rheumatologic disorders. 4. Rheumatoid arthritis and calcium pyrophosphate deposition disease. *Arch Phys Med Rehabil* 2005; 86: S61-68.

134. Pego-Reigosa JM, Rodriguez-Rodriguez M, Hurtado-Hernandez Z, et al. Calcium pyrophosphate deposition disease mimicking polymyalgia rheumatica: a prospective followup study of predictive factors for this condition in patients presenting with polymyalgia symptoms. *Arthritis Rheum* 2005; 53: 931–938.

135. Shah EN, Reddy NP, Rothschild BM. Fractal analysis of acceleration signals from patients with CPPD, rheumatoid arthritis, and spondyloarthroparthy of the finger joint. *Comput Methods Programs Biomed* 2005; 77: 233–239.

136. Suan JC, Chhem RK, Gati JS, et al. 4 T MRI of chondrocalcinosis in combination with three-dimensional CT, radiography, and arthroscopy: a report of three cases. *Skeletal Radiol* 2005; 34: 714–721.

137. Swayamprakasam AP, Taqvi S, Hossain S. A case of mistaken identity: pseudogout in a prosthetic knee. *Br J Hosp Med Lond Engl 2005* 2013; 74: 54–55.

138. Richette P, Ayoub G, Bardin T, et al. Hypomagnesemia and chondrocalcinosis in short bowel syndrome. *J Rheumatol* 2005; 32: 2434–2436.

139. Sato T, Hagiwara K, Sasaki M, et al. Crowned dens syndrome. *Intern Med Tokyo Jpn* 2005; 44: 160.

140. Tshering Vogel DW, Steinbach LS, Hertel R, et al. Acromioclavicular joint cyst: nine cases of a pseudotumor of the shoulder. *Skeletal Radiol* 2005; 34: 260–265.

141. Young-Min SA, Herbert L, Dick M, et al. Weekly alendronate-induced acute pseudogout. *Rheumatol Oxf Engl* 2005; 44: 131–132.

142. Wu DW, Reginato AJ, Torriani M, et al. The crowned dens syndrome as a cause of neck pain: report of two new cases and review of the literature. *Arthritis Rheum* 2005; 53: 133–137.

143. Wise CM. Crystal-associated arthritis in the elderly. *Clin Geriatr Med* 2005; 21: 491–511, v–vi.

144. Molloy ES, McCarthy GM. Calcium crystal deposition diseases: update on pathogenesis and manifestations. *Rheum Dis Clin North Am* 2006; 32: 383–400, vii.

145. Atzeni F, Sarzi-Puttini P, Bevilacqua M. Calcium deposition and associated chronic diseases (atherosclerosis, diffuse idiopathic skeletal hyperostosis, and others). *Rheum Dis Clin North Am* 2006; 32: 413–426, viii.

146. Lin S-H, Hsieh E-T, Wu T-Y, et al. Cervical myelopathy induced by pseudogout in ligamentum flavum and retro-odontoid mass: a case report. *Spinal Cord* 2006; 44: 692–694.

147. Cascone P, Rivaroli A, Arangio P, et al. Chondrocalcinosis: rare localization in the temporomandibular joint. *J Craniofac Surg* 2006; 17: 1189–1192.

148. Choi MH, MacKenzie JD, Dalinka MK. Imaging features of crystal-induced arthropathy. *Rheum Dis Clin North Am* 2006; 32: 427–446, viii.

149. Cohen MMJ. The new bone biology: pathologic, molecular, and clinical correlates. *Am J Med Genet A* 2006; 140: 2646–2706.

150. Rosenthal AK. Calcium crystal deposition and osteoarthritis. *Rheum Dis Clin North Am* 2006; 32: 401–412, vii.

151. Neogi T, Nevitt M, Niu J, et al. Lack of association between chondrocalcinosis and increased risk of cartilage loss in knees with osteoarthritis: results of two prospective longitudinal magnetic resonance imaging studies. *Arthritis Rheum* 2006; 54: 1822–1828.

152. Devauchelle-Pensec V, Berthelot JM, Jousse S, et al. Performance of hand radiographs in predicting the diagnosis in patients with early arthritis. *J Rheumatol* 2006; 33: 1511–1515.

153. De Filippo M, Sudberry JJ, Lombardo E, et al. Pathogenesis and evolution of carpal instability: imaging and topography. *Acta Bio-Medica Atenei Parm* 2006; 77: 168–180.

154. Harrington L, Schneider JI. Atraumatic joint and limb pain in the elderly. *Emerg Med Clin North Am* 2006; 24: 389–412, vii.

155. Gerster JC, Varisco PA, Kern J, et al. CPPD crystal deposition disease in patients with rheumatoid arthritis. *Clin Rheumatol* 2006; 25: 468–469.

156. Grassi W, Meenagh G, Pascual E, et al. ‘Crystal clear’-sonographic assessment of gout and calcium pyrophosphate deposition disease. *Semin Arthritis Rheum* 2006; 36: 197–202.

157. Feydy A, Lioté F, Carlier R, et al. Cervical spine and crystal-associated diseases: imaging findings. *Eur Radiol* 2006; 16: 459–468.

158. Ellman MH, Becker MA. Crystal-induced arthropathies: recent investigative advances. *Curr Opin Rheumatol* 2006; 18: 249–255.

159. Pytel P, Wollmann RL, Fessler RG, et al. Degenerative spine disease : pathologic findings in 985 surgical specimens. *Am J Clin Pathol* 2006; 125: 193–202.

160. Rajakulendran S, Smith D, Deighton C. Re: Aouba et al. Crowned dens syndrome misdiagnosed as polymyalgia rheumatica, giant cell arteritis, meningitis or spondylitis. *Rheumatol Oxf Engl* 2006; 45: 360–361.

161. Taggarshe D, Ng CH, Molokwu C, et al. Acute pseudogout following contrast angiography. *Clin Rheumatol* 2006; 25: 115–116.

162. Zhang Y, Terkeltaub R, Nevitt M, et al. Lower prevalence of chondrocalcinosis in Chinese subjects in Beijing than in white subjects in the United States: the Beijing Osteoarthritis Study. *Arthritis Rheum* 2006; 54: 3508–3512.

163. Watanabe H, Yamada S, Anayama S, et al. Pseudogout attack induced during etidronate disodium therapy. *Mod Rheumatol* 2006; 16: 117–119.

164. Josefina M, Ana CJ, Ariel V, et al. Development of pseudogout during etanercept treatment. *J Clin Rheumatol Pract Rep Rheum Musculoskelet Dis* 2007; 13: 177.

165. Mitsuyama H, Healey RM, Terkeltaub RA, et al. Calcification of human articular knee cartilage is primarily an effect of aging rather than osteoarthritis. *Osteoarthritis Cartilage* 2007; 15: 559–565.

166. Ames PRJ, Rainey MG. Consecutive pseudogout attacks after repetitive granulocyte colony-stimulating factor administration for neutropenia. *Mod Rheumatol* 2007; 17: 445–446.

167. Ariyoshi D, Imai K, Yamamoto S, et al. Subcutaneous tendon rupture of extensor tendons on bilateral wrists associated with calcium pyrophosphate dihydrate crystal deposition disease. *Mod Rheumatol* 2007; 17: 348–351.

168. Lam HY, Cheung KY, Law SW, et al. Crystal arthropathy of the lumbar spine: a report of 4 cases. *J Orthop Surg Hong Kong* 2007; 15: 94–101.

169. Cameron CR, Burgess CD. Recurrent back pain and fevers. *Med J Aust* 2007; 186: 208–209.

170. Sonsale PD, Philipson MR. Pseudogout after total knee arthroplasty. *J Arthroplasty* 2007; 22: 271–272.

171. Chollet-Janin A, Finckh A, Dudler J, et al. Methotrexate as an alternative therapy for chronic calcium pyrophosphate deposition disease: an exploratory analysis. *Arthritis Rheum* 2007; 56: 688–692.

172. Chong YY, Fong KY, Thumboo J. The value of joint aspirations in the diagnosis and management of arthritis in a hospital-based rheumatology service. *Ann Acad Med Singapore* 2007; 36: 106–109.

173. Nicholas BD, Smith JL 2nd, Kellman RM. Calcium pyrophosphate deposition of the temporomandibular joint with massive bony erosion. *J Oral Maxillofac Surg Off J Am Assoc Oral Maxillofac Surg* 2007; 65: 2086–2089.

174. De Geeter F, Goethals L, Piette Y, et al. Correlative imaging in crowned dens syndrome. *Clin Nucl Med* 2007; 32: 854–857.

175. Delle Sedie A, Riente L, Iagnocco A, et al. Ultrasound imaging for the rheumatologist X. Ultrasound imaging in crystal-related arthropathies. *Clin Exp Rheumatol* 2007; 25: 513–517.

176. Carlson AP, Yonas HM, Turner PT. Disorders of tumoral calcification of the spine: illustrative case study and review of the literature. *J Spinal Disord Tech* 2007; 20: 97–103.

177. Hirose CB, Wright RW. Calcium pyrophosphate dihydrate deposition disease (pseudogout) after total knee arthroplasty. *J Arthroplasty* 2007; 22: 273–276.

178. Giulioni M, Zucchelli M, Damiani S. Thoracic myelopathy caused by calcified ligamentum flavum. *Joint Bone Spine* 2007; 74: 504–505.

179. Goto S, Umehara J, Aizawa T, et al. Crowned Dens syndrome. *J Bone Joint Surg Am* 2007; 89: 2732–2736.

180. Filippou G, Frediani B, Gallo A, et al. A ‘new’ technique for the diagnosis of chondrocalcinosis of the knee: sensitivity and specificity of high-frequency ultrasonography. *Ann Rheum Dis* 2007; 66: 1126–1128.

181. Epis O, Caporali R, Scirè CA, et al. Efficacy of tidal irrigation in Milwaukee shoulder syndrome. *J Rheumatol* 2007; 34: 1545–1550.

182. Doumas C, Vazirani RM, Clifford PD, et al. Acute calcific periarthritis of the hand and wrist: a series and review of the literature. *Emerg Radiol* 2007; 14: 199–203.

183. Doita M, Shimomura T, Maeno K, et al. Calcium pyrophosphate dihydrate deposition in the transverse ligament of the atlas: an unusual cause of cervical myelopathy. *Skeletal Radiol* 2007; 36: 699–702.

184. Peach CA, Zhang Y, Dunford JE, et al. Cuff tear arthropathy: evidence of functional variation in pyrophosphate metabolism genes. *Clin Orthop* 2007; 462: 67–72.

185. Shah K, Spear J, Nathanson LA, et al. Does the presence of crystal arthritis rule out septic arthritis? *J Emerg Med* 2007; 32: 23–26.

186. Sethi KS, Garg A, Sharma MC, et al. Cervicomedullary compression secondary to massive calcium pyrophosphate crystal deposition in the atlantoaxial joint with intradural extension and vertebral artery encasement. *Surg Neurol* 2007; 67: 200–203.

187. Scutellari PN, Galeotti R, Leprotti S, et al. The crowned dens syndrome. Evaluation with CT imaging. *Radiol Med (Torino)* 2007; 112: 195–207.

188. Richette P, Ayoub G, Lahalle S, et al. Hypomagnesemia associated with chondrocalcinosis: a cross-sectional study. *Arthritis Rheum* 2007; 57: 1496–1501.

189. Scavarda D, Litre CF, Froelich S, et al. Cervical tumoral calcium pyrophosphate dihydrate deposition disease 28 years after suboccipital craniotomy: case report. *Neurosurgery* 2007; 60: E1151; discussion E1151.

190. Rothschild B. Suppressive treatment of calcium pyrophosphate deposition disease. *Arthritis Rheum* 2007; 56: 3172.

191. Rosenthal AK. Update in calcium deposition diseases. *Curr Opin Rheumatol* 2007; 19: 158–162.

192. Viriyavejkul P, Wilairatana V, Tanavalee A, et al. Comparison of characteristics of patients with and without calcium pyrophosphate dihydrate crystal deposition disease who underwent total knee replacement surgery for osteoarthritis. *Osteoarthritis Cartilage* 2007; 15: 232–235.

193. Volpe A, Caramaschi P, Thalheimer U, et al. Familiar association of Gitelman’s syndrome and calcium pyrophosphate dihydrate crystal deposition disease--a case report. *Rheumatol Oxf Engl* 2007; 46: 1506–1508.

194. Wakefield R, O’Connor P. All that glimmers is not gold. *Semin Arthritis Rheum* 2007; 37: 133–134.

195. Jennings F, Lambert E, Fredericson M. Rheumatic diseases presenting as sports-related injuries. *Sports Med Auckl NZ* 2008; 38: 917–930.

196. Albert A, Forthomme J-P, Vandenhooft A, et al. Are lesions of the posterior cruciate ligament predictable before knee arthroplasty? A histological study of 434 ligaments in osteoarthritic knees. *Acta Orthop Belg* 2008; 74: 652–658.

197. Announ N, Guerne P-A. Treating difficult crystal pyrophosphate dihydrate deposition disease. *Curr Rheumatol Rep* 2008; 10: 228–234.

198. Naqvi AH, Abraham JL, Kellman RM, et al. Calcium pyrophosphate dihydrate deposition disease (CPPD)/Pseudogout of the temporomandibular joint - FNA findings and microanalysis. *CytoJournal* 2008; 5: 8.

199. Ascani G, Pieramici T, Filosa A, et al. Pseudogout of the temporomandibular joint: a case report. *J Oral Maxillofac Surg Off J Am Assoc Oral Maxillofac Surg* 2008; 66: 386–388.

200. Martens HA, Lebbink HR, Posthumus MD. Clinical images: a patient with a tumor of the foot. *Arthritis Rheum* 2008; 58: 1085.

201. Brunot S, Fabre T, Lepreux S, et al. Pseudotumoral presentation of calcium pyrophosphate dihydrate crystal deposition disease. *J Rheumatol* 2008; 35: 727–729.

202. O’Connor KM, Williams P, Pergam SA. An unusual case of knee pain: pseudogout and Abiotrophia defectiva infection. *South Med J* 2008; 101: 961–962.

203. Ding T, Roddy E, Pande I. Tophaceous pseudogout--an unusual cause of nodulosis in rheumatoid arthritis. *Rheumatol Oxf Engl* 2008; 47: 608.

204. McGonagle D, Tan AL, Madden J, et al. Successful treatment of resistant pseudogout with anakinra. *Arthritis Rheum* 2008; 58: 631–633.

205. Nuki G. Colchicine: its mechanism of action and efficacy in crystal-induced inflammation. *Curr Rheumatol Rep* 2008; 10: 218–227.

206. Mikami T, Takeda Y, Ohira A, et al. Tumoral calcium pyrophosphate dihydrate crystal deposition disease of the temporomandibular joint: identification on crystallography. *Pathol Int* 2008; 58: 723–729.

207. Das SK, Farooqi A. Osteoarthritis. *Best Pract Res Clin Rheumatol* 2008; 22: 657–675.

208. Del Rincón I, Fernandez MP. Clinical images: compressive cervical myelopathy due to calcium pyrophosphate. *Arthritis Rheum* 2008; 58: 3631.

209. Checa A. Significance of meniscus extrusion in chondrocalcinosis: a sonographic and arthroscopic perspective. *J Rheumatol* 2008; 35: 1676.

210. Sivera F, Aragon R, Pascual E. First metatarsophalangeal joint aspiration using a 29-gauge needle. *Ann Rheum Dis* 2008; 67: 273–275.

211. Pawlotsky Y, Massart C, Guggenbuhl P, et al. Elevated parathyroid hormone 44-68 in idiopathic calcium pyrophosphate dihydrate crystal deposition disease. Role of menopause and iron metabolism? *J Rheumatol* 2008; 35: 315–318.

212. Pritzker KPH. Crystal deposition in joints: prevalence and relevance for arthritis. *J Rheumatol* 2008; 35: 958–959.

213. Sander O, Scherer A. Mimicry of a rheumatoid nodule by tophaceous pseudogout at the elbow. *J Rheumatol* 2008; 35: 1419.

214. Queiro R, Cañete J, Arostegui JI, et al. May some cases of intermittent hydrarthrosis represent an atypical form of calcium pyrophosphate dihydrate crystal deposition disease? Usefulness of mutational analysis of the MEFV gene. *Semin Arthritis Rheum* 2008; 37: 269–270.

215. Su K-Y, Lee H-T, Tsai C-Y. Recurrent calcium pyrophosphate dihydrate crystal deposition disease in a patient with rheumatoid arthritis--associated osteoporosis. *Eur J Intern Med* 2008; 19: 555–556.

216. Tan K-B, Scolyer RA, McCarthy SW, et al. Tumoural calcium pyrophosphate dihydrate crystal deposition disease (tophaceous pseudogout) of the hand: a report of two cases including one with a previously unreported associated florid reactive myofibroblastic proliferation. *Pathology (Phila)* 2008; 40: 719–722.

217. Rood MJ, van Laar JM, de Schepper AM, et al. The Milwaukee shoulder/knee syndrome. *J Clin Rheumatol Pract Rep Rheum Musculoskelet Dis* 2008; 14: 249–250.

218. Sarraf P, Kay J, Reginato AM. Non-crystalline and crystalline rheumatic disorders in chronic kidney disease. *Curr Rheumatol Rep* 2008; 10: 235–248.

219. Yamazaki H, Uchiyama S, Kato H. Median nerve and ulnar nerve palsy caused by calcium pyrophosphate dihydrate crystal deposition disease: case report. *J Hand Surg* 2008; 33: 1325–1328.

220. Valenti L, Fracanzani AL, Rossi V, et al. The hand arthropathy of hereditary hemochromatosis is strongly associated with iron overload. *J Rheumatol* 2008; 35: 153–158.

221. Yabuki S, Kikuchi S. Endoscopic surgery for cervical myelopathy due to calcification of the ligamentum flavum. *J Spinal Disord Tech* 2008; 21: 518–523.

222. Whelan BR, O’Shea F, McCarthy G. Pseudoneuropathic CPPD arthropathy: magnesium matters. *Rheumatol Oxf Engl* 2008; 47: 551–552.

223. Wendling D, Tisserand G, Griffond V, et al. Acute pseudogout after pamidronate infusion. *Clin Rheumatol* 2008; 27: 1205–1206.

224. Kalish LH, Ng T, Kalnins I, et al. Pseudogout mimicking an infratemporal fossa tumor. *Head Neck* 2010; 32: 127–132.

225. Nachimuthu S, Balamuthusamy S, Irimpen A, et al. Calcific constrictive pericarditis with refractory hypokalemia in a patient with Gitelman’s syndrome. *Am J Med Sci* 2009; 337: 74–76.

226. Announ N, Palmer G, Guerne P-A, et al. Anakinra is a possible alternative in the treatment and prevention of acute attacks of pseudogout in end-stage renal failure. *Joint Bone Spine* 2009; 76: 424–426.

227. Armas JB, Couto AR, Bettencourt BF. Spondyloarthritis, diffuse idiopathic skeletal hyperostosis (DISH) and chondrocalcinosis. *Adv Exp Med Biol* 2009; 649: 37–56.

228. Beck C, Morbach H, Richl P, et al. How can calcium pyrophosphate crystals induce inflammation in hypophosphatasia or chronic inflammatory joint diseases? *Rheumatol Int* 2009; 29: 229–238.

229. Bullocks JM, Downey CR, Gibler DPG, et al. Crystal deposition disease masquerading as proliferative tenosynovitis and its associated sequelae. *Ann Plast Surg* 2009; 62: 128–133.

230. Chen K-H, Li M-J, Cheng W-T, et al. Identification of monoclinic calcium pyrophosphate dihydrate and hydroxyapatite in human sclera using Raman microspectroscopy. *Int J Exp Pathol* 2009; 90: 74–78.

231. Ciapetti A, Filippucci E, Gutierrez M, et al. Calcium pyrophosphate dihydrate crystal deposition disease: sonographic findings. *Clin Rheumatol* 2009; 28: 271–276.

232. Richette P, Bardin T, Doherty M. An update on the epidemiology of calcium pyrophosphate dihydrate crystal deposition disease. *Rheumatol Oxf Engl* 2009; 48: 711–715.

233. Pascual E, Doherty M. Aspiration of normal or asymptomatic pathological joints for diagnosis and research: indications, technique and success rate. *Ann Rheum Dis* 2009; 68: 3–7.

234. Courtney P, Doherty M. Joint aspiration and injection and synovial fluid analysis. *Best Pract Res Clin Rheumatol* 2009; 23: 161–192.

235. Covani U, Orlando B, Galletti C, et al. Chondrocalcinosis of the temporomandibular joint: clinical considerations and case report. *Cranio J Craniomandib Pract* 2009; 27: 134–139.

236. Dalbeth N, McQueen FM. Use of imaging to evaluate gout and other crystal deposition disorders. *Curr Opin Rheumatol* 2009; 21: 124–131.

237. Rosenthal AK. Calcium crystals and arthritis: what is new under polarizing light? *J Clin Rheumatol Pract Rep Rheum Musculoskelet Dis* 2009; 15: 42–45.

238. Mehta BP, Shmerling RH, Moss AC. Pseudogout after polyethylene glycol bowel cleansing. *J Clin Gastroenterol* 2009; 43: 95–96.

239. Siva C, Velazquez C, Mody A, et al. Diagnosing acute monoarthritis in adults: a practical approach for the family physician. *Am Fam Physician* 2003; 68: 83–90.

240. Hamilton LC, Biant LC, Temple LN, et al. Isolated pseudogout diagnosed on hip arthroscopy. *J Bone Joint Surg Br* 2009; 91: 533–535.

241. Galed-Placed I, Carro-Rey E. Calcium pyrophosphate dihydrate crystal-induced arthritis: diagnosis by synovial fluid examination. *Acta Cytol* 2009; 53: 118–119.

242. Fuerst M, Niggemeyer O, Lammers L, et al. Articular cartilage mineralization in osteoarthritis of the hip. *BMC Musculoskelet Disord* 2009; 10: 166.

243. Fuerst M, Bertrand J, Lammers L, et al. Calcification of articular cartilage in human osteoarthritis. *Arthritis Rheum* 2009; 60: 2694–2703.

244. Frey ME, Dery FJJ, Cifu DX. C1-2 steroid injection for crowned dens syndrome. *PM R* 2009; 1: 379–382.

245. Filippucci E, Riveros MG, Georgescu D, et al. Hyaline cartilage involvement in patients with gout and calcium pyrophosphate deposition disease. An ultrasound study. *Osteoarthritis Cartilage* 2009; 17: 178–181.

246. Ellman MH. Images in clinical medicine. Chondrocalcinosis and hypomagnesemia. *N Engl J Med* 2009; 360: 71.

247. Ea H-K, Lioté F. Advances in understanding calcium-containing crystal disease. *Curr Opin Rheumatol* 2009; 21: 150–157.

248. Pritzker KPH. Counterpoint: Hydroxyapatite crystal deposition is not intimately involved in the pathogenesis and progression of human osteoarthritis. *Curr Rheumatol Rep* 2009; 11: 148–153.

249. Schlesinger N, Hassett AL, Neustadter L, et al. Does acute synovitis (pseudogout) occur in patients with chronic pyrophosphate arthropathy (pseudo-osteoarthritis)? *Clin Exp Rheumatol* 2009; 27: 940–944.

250. Unlu Z, Tarhan S, Ozmen EM. An idiopathic case of calcium pyrophosphate dihydrate crystal deposition disease with crowned dens syndrome in a young patient. *South Med J* 2009; 102: 949–951.

251. Yanai H, Yoshida H, Tada N. Clinical, radiological, and biochemical characteristics in patients with diseases mimicking polymyalgia rheumatica. *Clin Interv Aging* 2009; 4: 391–395.

252. Volpe A, Guerriero A, Marchetta A, et al. Familial hypocalciuric hypercalcemia revealed by chondrocalcinosis. *Joint Bone Spine* 2009; 76: 708–710.

253. Wener MH. Destructive temporomandibular joint disease: also consider tophaceous voracious (hydroxy)apatite. *J Rheumatol* 2009; 36: 453–454; author reply 454.

254. Maggio R, Garcia-Gonzalez E, Selvi E, et al. An acutely swollen knee. *BMJ* 2010; 341: c4441.

255. Ishikawa K, Furuya T, Noda K, et al. Crowned dens syndrome mimicking meningitis. *Intern Med Tokyo Jpn* 2010; 49: 2023.

256. Brent A, Hartley R. An unusual cause of recurrent falls in an older gentleman. *BMJ Case Rep*; 2010. Epub ahead of print 12 October 2010. DOI: 10.1136/bcr.04.2010.2910.

257. Carda S, Invernizzi M, Sainaghi PP, et al. Acute pseudogout following intravenous neridronate for osteoporosis. *J Rheumatol* 2010; 37: 1076.

258. Hsu Y-C, Chang C-W, Lin C-L, et al. Calcium pyrophosphate dihydrate deposition disease of the spleen. *Am J Surg* 2010; 200: e28-29.

259. Córdoba-Fernández A, Rayo-Rosado R. Pseudogout of the first metatarsophalangeal joint associated with hallux valgus: an atypical bilateral case. *J Am Podiatr Med Assoc* 2010; 100: 138–142.

260. Cranenburg ECM, Koos R, Schurgers LJ, et al. Characterisation and potential diagnostic value of circulating matrix Gla protein (MGP) species. *Thromb Haemost* 2010; 104: 811–822.

261. Dala-Ali BM, Welck M, Lloyd MA, et al. Pseudogout Associated Hip Pain in a Patient with HIV Infection. *Case Rep Med* 2010; 2010: 842814.

262. Dallos T, Sahinbegovic E, Aigner E, et al. Validation of a radiographic scoring system for haemochromatosis arthropathy. *Ann Rheum Dis* 2010; 69: 2145–2151.

263. Kathju S, Cohen R, Lasko L-A, et al. Pseudogout of the temporomandibular joint: immediate reconstruction with total joint arthroplasty. *Head Neck* 2010; 32: 406–410.

264. Kato H, Nishimoto K, Yoshikawa T, et al. Tophaceous pseudogout in the knee joint mimicking a soft-tissue tumour: a case report. *J Orthop Surg Hong Kong* 2010; 18: 118–121.

265. Nowatzky J, Howard R, Pillinger MH, et al. The role of uric acid and other crystals in osteoarthritis. *Curr Rheumatol Rep* 2010; 12: 142–148.

266. Kishore B, Khare P, Gupta RJ, et al. Tumoral calcium pyrophosphate dihydrate crystal deposition disease: a rare diagnosis by fine-needle aspiration. *Diagn Cytopathol* 2010; 38: 47–50.

267. Omura Y, Okamoto R, Konno M, et al. Problems in polarized light microscopy observation of birefringence of calcium pyrophosphate dihydrate crystals. *Micron Oxf Engl 1993* 2010; 41: 974–982.

268. Gutierrez M, Di Geso L, Filippucci E, et al. Calcium pyrophosphate crystals detected by ultrasound in patients without radiographic evidence of cartilage calcifications. *J Rheumatol* 2010; 37: 2602–2603.

269. Gutierrez M, Silveri F, Bertolazzi C, et al. Gitelman syndrome, calcium pyrophosphate dihydrate deposition disease and crowned dens syndrome. A new association? *Rheumatol Oxf Engl* 2010; 49: 610–613.

270. Fuerst M, Lammers L, Schäfer F, et al. Investigation of calcium crystals in OA knees. *Rheumatol Int* 2010; 30: 623–631.

271. Filippucci E, Scirè CA, Delle Sedie A, et al. Ultrasound imaging for the rheumatologist. XXV. Sonographic assessment of the knee in patients with gout and calcium pyrophosphate deposition disease. *Clin Exp Rheumatol* 2010; 28: 2–5.

272. Emary PC. Manual labor metacarpophalangeal arthropathy in a truck driver: a case report. *J Chiropr Med* 2010; 9: 193–199.

273. Dufauret-Lombard C, Vergne-Salle P, Simon A, et al. Ultrasonography in chondrocalcinosis. *Joint Bone Spine* 2010; 77: 218–221.

274. Sekijima Y, Yoshida T, Ikeda S-I. CPPD crystal deposition disease of the cervical spine: a common cause of acute neck pain encountered in the neurology department. *J Neurol Sci* 2010; 296: 79–82.

275. Pollock J, Giachino AA, Rakhra K, et al. SLAC wrist in the absence of recognised trauma and CPPD. *Hand Surg Int J Devoted Hand Up Limb Surg Relat Res J Asia-Pac Fed Soc Surg Hand* 2010; 15: 193–201.

276. Sahinbegovic E, Dallos T, Aigner E, et al. Musculoskeletal disease burden of hereditary hemochromatosis. *Arthritis Rheum* 2010; 62: 3792–3798.

277. Tamborrini G, Distler O, Schmet M, et al. Mixed crystal-induced arthropathy--a rare finding. *Clin Exp Rheumatol* 2010; 28: 801.

278. Taniguchi A, Ogita K, Murata T, et al. Painful neck on rotation: diagnostic significance for crowned dens syndrome. *J Neurol* 2010; 257: 132–135.

279. Zadaka A, Gioe T, Gertner E. Acute crystal-induced arthritis following arthroplasty. *J Knee Surg* 2010; 23: 17–20.

280. Viana SL, Fernandes JL, De Araújo Coimbra PP, et al. The ‘crowned dens’ revisited: imaging findings in calcium crystal deposition diseases around the odontoid. *J Neuroimaging Off J Am Soc Neuroimaging* 2010; 20: 311–323.

281. Wen HY, Schumacher HRJ, Zhang LY. Parathyroid disease. *Rheum Dis Clin North Am* 2010; 36: 647–664.

282. Meng J, Guo C, Luo H, et al. A case of destructive calcium pyrophosphate dihydrate crystal deposition disease of the temporomandibular joint: a diagnostic challenge. *Int J Oral Maxillofac Surg* 2011; 40: 1431–1437.

283. Jansen TL, Rasker JJ. Therapeutic consequences of crystals in the synovial fluid: a review for clinicians. *Clin Exp Rheumatol* 2011; 29: 1032–1039.

284. Mebarek S, Hamade E, Thouverey C, et al. Ankylosing spondylitis, late osteoarthritis, vascular calcification, chondrocalcinosis and pseudo gout: toward a possible drug therapy. *Curr Med Chem* 2011; 18: 2196–2203.

285. Abhishek A, Doherty M. Pathophysiology of articular chondrocalcinosis--role of ANKH. *Nat Rev Rheumatol* 2011; 7: 96–104.

286. Abhishek A, Doherty S, Maciewicz RA, et al. Self-reported knee malalignment in early adult life as an independent risk for knee chondrocalcinosis. *Arthritis Care Res* 2011; 63: 1550–1557.

287. Ali S, Hoch M, Dadhania V, et al. CPPD crowned dens syndrome with clivus destruction: a case report. *J Radiol Case Rep* 2011; 5: 30–37.

288. Arauz-Rivera R, Garcia-Porrua C. Crowned dens syndrome resembling meningitis as the first manifestation of calcium crystal deposition disease. *J Am Geriatr Soc* 2012; 60: 374–375.

289. Aran S, Malekzadeh S, Seifirad S. A double-blind randomized controlled trial appraising the symptom-modifying effects of colchicine on osteoarthritis of the knee. *Clin Exp Rheumatol* 2011; 29: 513–518.

290. Checa A, Wong H, Chun W. Sonographic characterization of mixed crystal arthropathy due to monosodium urate and calcium pyrophosphate dihydrate: the triple-contour sign. *J Ultrasound Med Off J Am Inst Ultrasound Med* 2011; 30: 861–862.

291. Siau K, Lee M, Laversuch CJ. Acute pseudogout of the neck--the crowned dens syndrome: 2 case reports and review of the literature. *Rheumatol Int* 2011; 31: 85–88.

292. Lahmer T, Ingerl D, Heemann U, et al. If the knee hurts, don’t forget the spine! *J Clin Neurosci Off J Neurosurg Soc Australas* 2011; 18: 424–425.

293. Dodakundi C, Hattori Y, Doi K, et al. Asymptomatic calcium pyrophosphate dihydrate deposition disease causing carpal tunnel syndrome: case report. *Hand Surg Int J Devoted Hand Up Limb Surg Relat Res J Asia-Pac Fed Soc Surg Hand* 2012; 17: 89–92.

294. Nguyen C, Ea HK, Thiaudiere D, et al. Calcifications in human osteoarthritic articular cartilage: ex vivo assessment of calcium compounds using XANES spectroscopy. *J Synchrotron Radiat* 2011; 18: 475–480.

295. Mukhopadhyay S, Guha A, Perera A. Monoarticular pseudogout of the hip presenting as septic arthritis: a case report. *Acta Orthop Traumatol Turc* 2011; 45: 200–202.

296. Di Geso L, Tardella M, Gutierrez M, et al. Crystal deposition at elbow hyaline cartilage: the sonographic perspective. *J Clin Rheumatol Pract Rep Rheum Musculoskelet Dis* 2011; 17: 344–345.

297. Niggemeyer O, Steinhagen J, Zustin J, et al. The value of routine histopathology during hip arthroplasty in patients with degenerative and inflammatory arthritis. *Hip Int J Clin Exp Res Hip Pathol Ther* 2011; 21: 98–106.

298. De Marco G, Rampini C, Ferri E, et al. A rare case of chondrocalcinosis in the left sterno-clavicular joint. *Rheumatol Oxf Engl* 2011; 50: 2317–2318.

299. Demertzis JL, Rubin DA. MR imaging assessment of inflammatory, crystalline-induced, and infectious arthritides. *Magn Reson Imaging Clin N Am* 2011; 19: 339–363.

300. Pandit H, Jenkins C, Gill HS, et al. Unnecessary contraindications for mobile-bearing unicompartmental knee replacement. *J Bone Joint Surg Br* 2011; 93: 622–628.

301. Sklenicka S, Dierks EJ, Jarmin J, et al. Pseudogout of the temporomandibular joint: an uncommon cause of temporomandibular joint pain and swelling. *Oral Surg Oral Med Oral Pathol Oral Radiol Endod* 2011; 111: 709–714.

302. Skeete K, Hess EP, Clark T, et al. Epidemiology of suspected wrist joint infection versus inflammation. *J Hand Surg* 2011; 36: 469–474.

303. Hernigou P, Pascale W, Pascale V, et al. Does primary or secondary chondrocalcinosis influence long-term survivorship of unicompartmental arthroplasty? *Clin Orthop* 2012; 470: 1973–1979.

304. Favero M, Calò LA, Schiavon F, et al. Miscellaneous non-inflammatory musculoskeletal conditions. Bartter’s and Gitelman’s diseases. *Best Pract Res Clin Rheumatol* 2011; 25: 637–648.

305. Erhardt MA, Vetter SY, Suda AJ, et al. Mimicry in older patients: tophaceous pseudogout as a tumorlike lesion: a case report. *J Am Podiatr Med Assoc* 2011; 101: 462–465.

306. Ea H-K, Nguyen C, Bazin D, et al. Articular cartilage calcification in osteoarthritis: insights into crystal-induced stress. *Arthritis Rheum* 2011; 63: 10–18.

307. Seybold JD, Dahl WJ, Kadakia AR. Tophaceous pseudogout of the ankle: case report. *Foot Ankle Int* 2011; 32: 717–721.

308. Rim PC, Keith MP. Chondrocalcinosis and hypomagnesemia in a 26-year-old woman. *J Clin Rheumatol Pract Rep Rheum Musculoskelet Dis* 2011; 17: 334–335.

309. Robier C, Neubauer M, Stettin M, et al. Microscopic examination of stained cytospin preparations is a reliable method for the detection of calcium pyrophosphate crystals in synovial fluid. *Scand J Rheumatol* 2011; 40: 406–407.

310. Robier C, Neubauer M, Quehenberger F, et al. Coincidence of calcium pyrophosphate and monosodium urate crystals in the synovial fluid of patients with gout determined by the cytocentrifugation technique. *Ann Rheum Dis* 2011; 70: 1163–1164.

311. Robier C, Neubauer M, Quehenberger F, et al. Calcium pyrophosphate and monosodium urate crystals in synovial fluid as a cause of pseudoeosinophilia. *Clin Chem Lab Med* 2011; 49: 1345–1347.

312. Robier C, Neubauer M, Gross H, et al. Crystal-associated pseudoeosinophilia of synovial fluid. *Clin Chem Lab Med* 2011; 49: 163–164.

313. Rosenthal AK. Crystals, inflammation, and osteoarthritis. *Curr Opin Rheumatol* 2011; 23: 170–173.

314. Rosenthal AK, Ryan LM. Crystal arthritis: calcium pyrophosphate deposition-nothing ‘pseudo’ about it! *Nat Rev Rheumatol* 2011; 7: 257–258.

315. Zhang W, Doherty M, Bardin T, et al. European League Against Rheumatism recommendations for calcium pyrophosphate deposition. Part I: terminology and diagnosis. *Ann Rheum Dis* 2011; 70: 563–570.

316. Zhang W, Doherty M, Pascual E, et al. EULAR recommendations for calcium pyrophosphate deposition. Part II: management. *Ann Rheum Dis* 2011; 70: 571–575.

317. Yoo Y, Seo Y-J, Huh M, et al. Gout and coexisting pseudogout in the knee joint. *Knee Surg Sports Traumatol Arthrosc Off J ESSKA* 2011; 19: 553–555.

318. Ogawa Y, Nagatsuma M, Kubota G, et al. Acute lumbar spinal pseudogout attack after instrumented surgery. *Spine* 2012; 37: E1529-1533.

319. Levi GS, Sadr K, Scuderi GR. Bilateral pseudogout 8 years after bilateral total knee arthroplasty. *Orthop Clin North Am* 2012; 43: e59-62.

320. Ivory D, Velázquez CR. The forgotten crystal arthritis: calcium pyrophosphate deposition. *Mo Med* 2012; 109: 64–68.

321. Agrawal A, Purandare N, Sridhar E, et al. Imaging findings in a rare case of extra-articular chondrocalcinosis. *Clin Nucl Med* 2012; 37: 184–187.

322. Abhishek A, Doherty S, Maciewicz R, et al. Chondrocalcinosis is common in the absence of knee involvement. *Arthritis Res Ther* 2012; 14: R205.

323. Hunte TC, Bernstein HM, Dickinson GM. Acute crystalline arthritis in an artificial knee. *J Clin Rheumatol Pract Rep Rheum Musculoskelet Dis* 2012; 18: 203–204.

324. Hujazi I, Ambler G, Arora A, et al. Role of Newman’s classification in predicting outcomes in patients with crystal arthritis. *Int Orthop* 2012; 36: 1287–1290.

325. Andres M, Sivera F, Pascual E. Methotrexate is an option for patients with refractory calcium pyrophosphate crystal arthritis. *J Clin Rheumatol Pract Rep Rheum Musculoskelet Dis* 2012; 18: 234–236.

326. Antohe JL, Delvecchio B, Harrington TM. An unusual presentation of acute calcium pyrophosphate dihydrate arthropathy of the sternoclavicular joint in a patient with systemic lupus erythematosus. *J Clin Rheumatol Pract Rep Rheum Musculoskelet Dis* 2012; 18: 162.

327. Namazie MR bin M, Fosbender MR. Calcium pyrophosphate dihydrate crystal deposition of multiple lumbar facet joints: a case report. *J Orthop Surg Hong Kong* 2012; 20: 254–256.

328. Lee J, Cho K-T, Kim E-J. Cauda equina syndrome caused by pseudogout involving the lumbar intervertebral disc. *J Korean Med Sci* 2012; 27: 1591–1594.

329. Bilge ŞY, Korkmaz C. Calcium metabolism disorders simulating rheumatologic diseases. *Rheumatol Int* 2012; 32: 1503–1505.

330. Beggs AE, Reeves DJ, Noel NS. Leukopenia associated with long-term colchicine administration. *Am J Health-Syst Pharm AJHP Off J Am Soc Health-Syst Pharm* 2012; 69: 2147–2148.

331. Moltó A, Ea H-K, Richette P, et al. Efficacy of anakinra for refractory acute calcium pyrophosphate crystal arthritis. *Joint Bone Spine* 2012; 79: 621–623.

332. Zweifel D, Ettlin D, Schuknecht B, et al. Tophaceuos calcium pyrophosphate dihydrate deposition disease of the temporomandibular joint: the preferential site? *J Oral Maxillofac Surg Off J Am Assoc Oral Maxillofac Surg* 2012; 70: 60–67.

333. Matsumura M, Hara S. Images in clinical medicine. Crowned dens syndrome. *N Engl J Med* 2012; 367: e34.

334. Ciancio G, Bortoluzzi A, Govoni M. Epidemiology of gout and chondrocalcinosis. *Reumatismo* 2012; 63: 207–220.

335. Magarelli N, Amelia R, Melillo N, et al. Imaging of chondrocalcinosis: calcium pyrophosphate dihydrate (CPPD) crystal deposition disease -- imaging of common sites of involvement. *Clin Exp Rheumatol* 2012; 30: 118–125.

336. Couderc M, Soubrier M. Reply to the comment by Verhoeven et al. about the article ‘Efficacy of anakinra in articular chondrocalcinosis--report of three cases’ Joint Bone Spine 2012;79:330-1. *Joint Bone Spine* 2013; 80: 116.

337. Diamantopoulos AP, Brodin C, Hetland H, et al. Interleukin 1β blockade improves signs and symptoms of chronic calcium pyrophosphate crystal arthritis resistant to treatment. *J Clin Rheumatol Pract Rep Rheum Musculoskelet Dis* 2012; 18: 310–311.

338. Minoda M, Matsumoto T, Kubo S, et al. Multiple huge subchondral cysts associated with pseudogout in the bilateral knees: a case report and review of the literatures. *J Orthop Sci Off J Jpn Orthop Assoc* 2012; 17: 817–821.

339. Di Geso L, Filippucci E, Gutierrez M, et al. Calcium pyrophosphate deposition: sonographic features in a familial case. *J Rheumatol* 2012; 39: 1488–1490.

340. Marson P, Pasero G. Some historical remarks on microcrystalline arthritis (gout and chondrocalcinosis). *Reumatismo* 2012; 63: 199–206.

341. Matsumura Y, Nomura J, Nakanishi K, et al. Synovial chondromatosis of the temporomandibular joint with calcium pyrophosphate dihydrate crystal deposition disease (pseudogout). *Dento Maxillo Facial Radiol* 2012; 41: 703–707.

342. Macmullan P, McCarthy G. Treatment and management of pseudogout: insights for the clinician. *Ther Adv Musculoskelet Dis* 2012; 4: 121–131.

343. Oda Y, Ooi S, Urushidani Y, et al. Crowned dens syndrome. *Intern Med Tokyo Jpn* 2012; 51: 231.

344. Odate S, Shikata J, Fujibayashi S, et al. Progressive thoracic myelopathy caused by spinal calcium pyrophosphate crystal deposition because of proximal junctional vertebral compression fracture after lumbopelvic fusion. *Eur Spine J Off Publ Eur Spine Soc Eur Spinal Deform Soc Eur Sect Cerv Spine Res Soc* 2012; 21: 2436–2442.

345. Hosseinian Amiri A, Rafiei A. Alkaptonuria in a middle-aged female. *Casp J Intern Med* 2012; 3: 554–556.

346. Gruber BL, Couto AR, Armas JB, et al. Novel ANKH amino terminus mutation (Pro5Ser) associated with early-onset calcium pyrophosphate disease with associated phosphaturia. *J Clin Rheumatol Pract Rep Rheum Musculoskelet Dis* 2012; 18: 192–195.

347. Hahn M, Raithel M, Hagel A, et al. Chronic calcium pyrophosphate crystal inflammatory arthritis induced by extreme hypomagnesemia in short bowel syndrome. *BMC Gastroenterol* 2012; 12: 129.

348. Filippou G, Frediani B. The diagnosis of calcium pyrophosphate dihydrate crystal deposition disease: the good, the bad and… ultrasonography! *Reumatismo* 2012; 64: 125–127.

349. Filippou G, Bozios P, Gambera D, et al. Ultrasound detection of calcium pyrophosphate dihydrate crystal deposits in menisci: a pilot in vivo and ex vivo study. *Ann Rheum Dis* 2012; 71: 1426–1427.

350. Filippucci E, Di Geso L, Grassi W. Tips and tricks to recognize microcrystalline arthritis. *Rheumatol Oxf Engl* 2012; 51 Suppl 7: vii18-21.

351. Ellabban AS, Kamel SR, Omar HASA, et al. Ultrasonographic diagnosis of articular chondrocalcinosis. *Rheumatol Int* 2012; 32: 3863–3868.

352. Ellabban AS, Kamel SR, Abo Omar HAS, et al. Ultrasonographic findings of Achilles tendon and plantar fascia in patients with calcium pyrophosphate deposition disease. *Clin Rheumatol* 2012; 31: 697–704.

353. Doghramji PP, Mandell BF, Pope RS. Casebook consults: improving outcomes in gout (multimedia activity). *Am J Med* 2012; 125: S1.

354. Seror P, Vuillemin V. Ulnar nerve lesion at the wrist related to pisotriquetral joint arthropathy. *Muscle Nerve* 2013; 47: 600–604.

355. Popov HI, Gherman C, Rogojan L, et al. Milwaukee shoulder syndrome associated with pigmented villonodular synovitis. Case report. *Med Ultrason* 2012; 14: 67–70.

356. Salar O, Mushtaq F, Ahmed M. Calcium pyrophosphate dihydrate deposition in the trochanteric hip bursa presenting as acute hip pain. *BMJ Case Rep*; 2012. Epub ahead of print 10 July 2012. DOI: 10.1136/bcr.12.2011.5426.

357. Reed MJ, Carachi A. Management of the nontraumatic hot swollen joint. *Eur J Emerg Med Off J Eur Soc Emerg Med* 2012; 19: 103–107.

358. Srinivasan V, Kesler H, Johnson M, et al. Tophaceous pseudogout of the thoracic spine. *Acta Neurochir (Wien)* 2012; 154: 747–750; discussion 750.

359. Srinivasan V, Wensel A, Dutcher P, et al. Calcium pyrophosphate deposition disease of the temporomandibular joint. *J Neurol Surg Rep* 2012; 73: 6–8.

360. Sussmann AR, Cohen J, Nomikos GC, et al. Magnetic resonance imaging of shoulder arthropathies. *Magn Reson Imaging Clin N Am* 2012; 20: 349–371, xi–xii.

361. Rho YH, Zhu Y, Zhang Y, et al. Risk factors for pseudogout in the general population. *Rheumatol Oxf Engl* 2012; 51: 2070–2074.

362. Rosales Alexander JL, Magro Checa C, Salvatierra J, et al. Recurrent parotid pseudogout. *Rheumatol Oxf Engl* 2012; 51: 2169.

363. Terkeltaub R. Imaging joints for calcium pyrophosphate crystal deposition: a knock to the knees. *Arthritis Res Ther* 2012; 14: 128.

364. Wendling D, Martin M, Guillot X, et al. Interspinous bursitis and chondrocalcinosis. *Joint Bone Spine* 2012; 79: 516.

365. Roubille F, Kritikou E, Busseuil D, et al. Colchicine: an old wine in a new bottle? *Anti-Inflamm Anti-Allergy Agents Med Chem* 2013; 12: 14–23.

366. Lv H, Fan Z, Han Y, et al. A case of pseudogout of the temporomandibular joint with giant cell reparative granuloma of the temporal bone. *Am J Otolaryngol* 2013; 34: 762–765.

367. Kenzaka T, Wakabayashi T, Morita Y. Acute crystal deposition arthritis of the pubic symphysis. *BMJ Case Rep*; 2013. Epub ahead of print 16 April 2013. DOI: 10.1136/bcr-2013-009239.

368. Abhishek A, Doherty S, Maciewicz R, et al. Evidence of a systemic predisposition to chondrocalcinosis and association between chondrocalcinosis and osteoarthritis at distant joints: a cross-sectional study. *Arthritis Care Res* 2013; 65: 1052–1058.

369. Adinolfi A, Picerno V, Di Sabatino V, et al. Inquiry is fatal to certainty-is the ultrasonography double contour sign specific for uric acid-induced arthritis? *Arthritis Rheum* 2013; 65: 1952.

370. Bahk W-J, Chang E-D, Lee A-H, et al. Huge tophaceous pseudogout associated with tenosynovial chondromatosis arising from flexor digitorum tendon sheaths of the foot: a case report. *Skeletal Radiol* 2013; 42: 1755–1759.

371. Barskova VG, Kudaeva FM, Bozhieva LA, et al. Comparison of three imaging techniques in diagnosis of chondrocalcinosis of the knees in calcium pyrophosphate deposition disease. *Rheumatol Oxf Engl* 2013; 52: 1090–1094.

372. Cacciotti G, Novegno F, Fiume D. Calcium pyrophosphate dihydrate deposition disease of the filum terminale. *Eur Spine J Off Publ Eur Spine Soc Eur Spinal Deform Soc Eur Sect Cerv Spine Res Soc* 2013; 22 Suppl 3: S501-505.

373. Chakravarty SD, Markenson JA. Rheumatic manifestations of endocrine disease. *Curr Opin Rheumatol* 2013; 25: 37–43.

374. Chang EY, Lim WY, Wolfson T, et al. Frequency of atlantoaxial calcium pyrophosphate dihydrate deposition at CT. *Radiology* 2013; 269: 519–524.

375. Dirim B, Resnick D, Abreu M, et al. Relationship between the degeneration of the cruciate ligaments and calcium pyrophosphate dihydrate crystal deposition: anatomic, radiologic study with histologic correlation. *Clin Imaging* 2013; 37: 342–347.

376. Morita T, Tanimoto T, Kaji S, et al. Poststroke crowned dens syndrome. *Spine J Off J North Am Spine Soc* 2013; 13: 1161–1162.

377. Damian LO, Felea I, Boloşiu C, et al. A case of alkaptonuria - ultrasonographic findings. *Med Ultrason* 2013; 15: 321–325.

378. O’Connor PJ. Crystal deposition disease and psoriatic arthritis. *Semin Musculoskelet Radiol* 2013; 17: 74–79.

379. Oliviero F, Scanu A, Galozzi P, et al. Prevalence of calcium pyrophosphate and monosodium urate crystals in synovial fluid of patients with previously diagnosed joint diseases. *Joint Bone Spine* 2013; 80: 287–290.

380. Ottaviani S, Brunier L, Sibilia J, et al. Efficacy of anakinra in calcium pyrophosphate crystal-induced arthritis: a report of 16 cases and review of the literature. *Joint Bone Spine* 2013; 80: 178–182.

381. Hakozaki M, Fukuda H, Tajino T, et al. Remitting seronegative symmetrical synovitis with pitting edema syndrome caused by crystal-induced arthritis of the wrist: a case report. *Med Princ Pract Int J Kuwait Univ Health Sci Cent* 2013; 22: 307–310.

382. Godfrin-Valnet M, Godfrin G, Godard J, et al. Eighteen cases of crowned dens syndrome: Presentation and diagnosis. *Neurochirurgie* 2013; 59: 115–120.

383. Garcia-Gonzalez E, Baldi C, Guidelli GM, et al. Crowned dens syndrome and cervical interspinous bursitis mimicking acute meningitis. *J Clin Rheumatol Pract Rep Rheum Musculoskelet Dis* 2013; 19: 357–358.

384. Filippou G, Adinolfi A, Bozios P, et al. Do not hallow until you are out of the wood! Ultrasonographic detection of CPP crystal deposits in menisci: facts and pitfalls. *ScientificWorldJournal* 2013; 2013: 181826.

385. Filippou G, Filippucci E, Tardella M, et al. Extent and distribution of CPP deposits in patients affected by calcium pyrophosphate dihydrate deposition disease: an ultrasonographic study. *Ann Rheum Dis* 2013; 72: 1836–1839.

386. Filippucci E, Delle Sedie A, Riente L, et al. Ultrasound imaging for the rheumatologist. XLVII. Ultrasound of the shoulder in patients with gout and calcium pyrophosphate deposition disease. *Clin Exp Rheumatol* 2013; 31: 659–664.

387. Parperis K, Carrera G, Baynes K, et al. The prevalence of chondrocalcinosis (CC) of the acromioclavicular (AC) joint on chest radiographs and correlation with calcium pyrophosphate dihydrate (CPPD) crystal deposition disease. *Clin Rheumatol* 2013; 32: 1383–1386.

388. Suresh E. Problem based review: The patient with acute monoarthritis. *Acute Med* 2013; 12: 111–116.

389. Robier C, Neubauer M, Fritz K, et al. The detection of calcium pyrophosphate crystals in sequential synovial fluid examinations of patients with osteoarthritis: once positive, always positive. *Clin Rheumatol* 2013; 32: 671–672.

390. Takahashi T, Minakata Y, Tamura M, et al. A rare case of crowned dens syndrome mimicking aseptic meningitis. *Case Rep Neurol* 2013; 5: 40–46.

391. Russell E, Checa A. Potential pitfalls in the diagnosis of crystal-induced arthritis and in the sonographic measurement of hyaline cartilage due to the presence of joint effusion. *J Clin Rheumatol Pract Rep Rheum Musculoskelet Dis* 2013; 19: 98–100.

392. Tausche A-K, Gehrisch S, Panzner I, et al. A 3-day delay in synovial fluid crystal identification did not hinder the reliable detection of monosodium urate and calcium pyrophosphate crystals. *J Clin Rheumatol Pract Rep Rheum Musculoskelet Dis* 2013; 19: 241–245.

393. Touraine S, Ea HK, Bousson V, et al. Chondrocalcinosis of femoro-tibial and proximal tibio-fibular joints in cadaveric specimens: a high-resolution CT imaging study of the calcification distribution. *PloS One* 2013; 8: e54955.

394. Uh M, Dewar C, Spouge D, et al. Crowned dens syndrome: a rare cause of acute neck pain. *Clin Rheumatol* 2013; 32: 711–714.

395. Ungprasert P, Kaewpoowat Q, Ratapano S, et al. Presence of crystals is not an evidence of absence of infection. *Am J Emerg Med* 2013; 31: 455.e1–2.

396. Rothschild BM. Distinguishing erosive osteoarthritis and calcium pyrophosphate deposition disease. *World J Orthop* 2013; 4: 29–31.

397. Yamazaki Y, Kanaya Y, Naka H, et al. Severe occipital pain caused by periodontoid calcifications: crowned dens syndrome. *Cephalalgia Int J Headache* 2013; 33: 425.

398. Verhoeven F, Prati C, Godfrin-Valnet M, et al. IL1 blockade in crystal-induced arthritis: impact of disease duration and the inflammatory syndrome. Comments on the article by Couderc M. et al. ‘Efficacy of anakinra in articular chondrocalcinosis’. *Joint Bone Spine* 2013; 80: 115–116.

399. Wakasugi T, Shirasaka R, Kimura H, et al. Flexor tendon rupture of the little finger caused by calcium pyrophosphate dihydrate crystal deposition disease of the pisotriquetrum joint. *Hand Surg Int J Devoted Hand Up Limb Surg Relat Res J Asia-Pac Fed Soc Surg Hand* 2013; 18: 413–415.

400. Reis GF, Perry A. A 67-year-old man with a lumbar spine lesion. *Brain Pathol Zurich Switz* 2014; 24: 547–548.

401. Matsuda S, Kawasaki M, Ichinari H, et al. A pseudo-iliopsoas abscess. *Intern Med Tokyo Jpn* 2014; 53: 1729.

402. Kobayashi T, Miyakoshi N, Konno N, et al. Acute neck pain caused by arthritis of the lateral atlantoaxial joint. *Spine J* 2014; 14: 1909–1913.

403. Jimbo R, Iida H, Ishibashi Y. Tophaceous pseudogout in a patient undergoing peritoneal dialysis. *CEN Case Rep* 2014; 3: 148–151.

404. Abdelsayed RA, Said-Al-Naief N, Salguerio M, et al. Tophaceous pseudogout of the temporomandibular joint: a series of 3 cases. *Oral Surg Oral Med Oral Pathol Oral Radiol* 2014; 117: 369–375.

405. Abhishek A, Doherty S, Maciewicz R, et al. The association between ANKH promoter polymorphism and chondrocalcinosis is independent of age and osteoarthritis: results of a case-control study. *Arthritis Res Ther* 2014; 16: R25.

406. Aichmair A, Herzog RJ, Perino G, et al. Recovery after cervical decompression surgery for the treatment of crowned dens syndrome causing progressive neurological decline: a case report. *HSS J Musculoskelet J Hosp Spec Surg* 2014; 10: 83–87.

407. Abhishek A. Calcium pyrophosphate deposition. *Br J Hosp Med Lond Engl 2005* 2014; 75: C61-64.

408. Abhishek A, Doherty M. Epidemiology of calcium pyrophosphate crystal arthritis and basic calcium phosphate crystal arthropathy. *Rheum Dis Clin North Am* 2014; 40: 177–191.

409. Abhishek A, Doherty S, Maciewicz R, et al. Association between low cortical bone mineral density, soft-tissue calcification, vascular calcification and chondrocalcinosis: a case-control study. *Ann Rheum Dis* 2014; 73: 1997–2002.

410. Husar-Memmer E, Stadlmayr A, Datz C, et al. HFE-related hemochromatosis: an update for the rheumatologist. *Curr Rheumatol Rep* 2014; 16: 393.

411. Lee GS, Kim RS, Park HK, et al. Crowned dens syndrome: a case report and review of the literature. *Korean J Spine* 2014; 11: 15–17.

412. Bruges-Armas J, Bettencourt BF, Couto AR, et al. Effectiveness and safety of infliximab in two cases of severe chondrocalcinosis: nine years of follow-up. *Case Rep Rheumatol* 2014; 2014: 536856.

413. Kuriyama A. Crowned dens syndrome. *CMAJ Can Med Assoc J* 2014; 186: 293.

414. Kim H-R, Lee J-H, Kim NR, et al. Detection of calcium pyrophosphate dihydrate crystal deposition disease by dual-energy computed tomography. *Korean J Intern Med* 2014; 29: 404–405.

415. Huang M, Schweitzer ME. The role of radiology in the evolution of the understanding of articular disease. *Radiology* 2014; 273: S1-22.

416. Lin Y-Y, Wang T-G, Li K-J, et al. Imaging characteristics of calcium pyrophosphate dihydrate crystal deposition disease. *Am J Phys Med Rehabil* 2014; 93: 272–273.

417. McQueen FM, Doyle A, Dalbeth N. Imaging in the crystal arthropathies. *Rheum Dis Clin North Am* 2014; 40: 231–249.

418. Patel J, Girishkumar, Mruthyunjaya, et al. Bilateral Olecranon Bursitis - A Rare Clinical presentation of Calcium Pyrophosphate Crystal Deposition Disease. *J Orthop Case Rep* 2014; 4: 3–6.

419. MuȘetescu AE, Ciurea P, RoȘu A. Musculoskeletal Ultrasound Diagnosis in Calcium Pyrophosphate Dihydrate Crystal Deposition Disease. *Curr Health Sci J* 2014; 40: 293–295.

420. Nguyen JC, De Smet AA, Graf BK, et al. MR imaging-based diagnosis and classification of meniscal tears. *Radiogr Rev Publ Radiol Soc N Am Inc* 2014; 34: 981–999.

421. Klineberg E, Bui T, Schlenk R, et al. Retro-odontoid calcium pyrophosphate dehydrate deposition: surgical management and review of the literature. *Evid-Based Spine-Care J* 2014; 5: 63–69.

422. Daoussis D, Antonopoulos I, Andonopoulos AP. ACTH as a treatment for acute crystal-induced arthritis: update on clinical evidence and mechanisms of action. *Semin Arthritis Rheum* 2014; 43: 648–653.

423. deSouza RM, Uff C, Galloway M, et al. Spinal epidural hematoma caused by pseudogout: a case report and literature review. *Glob Spine J* 2014; 4: 105–108.

424. Hanai S, Sato T, Nagatani K, et al. Pseudogout of the sternoclavicular joints. *Intern Med Tokyo Jpn* 2014; 53: 521–522.

425. Gutierrez M, Di Geso L, Salaffi F, et al. Ultrasound detection of cartilage calcification at knee level in calcium pyrophosphate deposition disease. *Arthritis Care Res* 2014; 66: 69–73.

426. Grobost V, Vayssade M, Roche A, et al. Axial calcium pyrophosphate dihydrate deposition disease revealed by recurrent sterile spondylodiscitis and epidural abscess. *Joint Bone Spine* 2014; 81: 180–182.

427. Finckh A, Mc Carthy GM, Madigan A, et al. Methotrexate in chronic-recurrent calcium pyrophosphate deposition disease: no significant effect in a randomized crossover trial. *Arthritis Res Ther* 2014; 16: 458.

428. Shirazian H, Chang EY, Wolfson T, et al. Prevalence of sternoclavicular joint calcium pyrophosphate dihydrate crystal deposition on computed tomography. *Clin Imaging* 2014; 38: 380–383.

429. Filippou G, Picerno V, Adinolfi A, et al. Change perspective to increase diagnostic accuracy of ultrasonography in calcium pyrophosphate dehydrate deposition disease! A new approach: the axial scan of the meniscus. *Reumatismo* 2015; 66: 318–321.

430. Filippucci E, Di Geso L, Girolimetti R, et al. Ultrasound in crystal-related arthritis. *Clin Exp Rheumatol* 2014; 32: S42-47.

431. Ea H-K, Lioté F. Diagnosis and clinical manifestations of calcium pyrophosphate and basic calcium phosphate crystal deposition diseases. *Rheum Dis Clin North Am* 2014; 40: 207–229.

432. Durcan L, Bolster F, Kavanagh EC, et al. The structural consequences of calcium crystal deposition. *Rheum Dis Clin North Am* 2014; 40: 311–328.

433. Pascart T, Richette P, Flipo R-M. Treatment of nongout joint deposition diseases: an update. *Arthritis* 2014; 2014: 375202.

434. Shamil E, Willems S, Grolman W, et al. Pseudogout in the middle ear. *Otol Neurotol Off Publ Am Otol Soc Am Neurotol Soc Eur Acad Otol Neurotol* 2014; 35: e202-203.

435. Seeger LL, Sako E, Motamedi K, et al. Intra-articular calcifications following arthroscopic ACL reconstruction: prevalence and possible significance. *Skeletal Radiol* 2014; 43: 209–212.

436. Ramonda R, Frallonardo P, Oliviero F, et al. Pain and microcrystalline arthritis. *Reumatismo* 2014; 66: 48–56.

437. Robier C, Quehenberger F, Neubauer M, et al. The cytospin technique improves the detection of calcium pyrophosphate crystals in synovial fluid samples with a low leukocyte count. *Rheumatol Int* 2014; 34: 773–776.

438. Rosales-Alexander JL, Balsalobre Aznar J, Magro-Checa C. Calcium pyrophosphate crystal deposition disease: diagnosis and treatment. *Open Access Rheumatol Res Rev* 2014; 6: 39–47.

439. Theiler G, Quehenberger F, Rainer F, et al. The detection of calcium pyrophosphate crystals in the synovial fluid of patients with rheumatoid arthritis using the cytospin technique: prevalence and clinical correlation. *Rheumatol Int* 2014; 34: 137–139.

440. Ryu K, Iriuchishima T, Oshida M, et al. The prevalence of and factors related to calcium pyrophosphate dihydrate crystal deposition in the knee joint. *Osteoarthritis Cartilage* 2014; 22: 975–979.

441. Tischler BT, Diaz LE, Murakami AM, et al. Scapholunate advanced collapse: a pictorial review. *Insights Imaging* 2014; 5: 407–417.

442. Toprak H, Kılıç E, Serter A, et al. Doppler US in rheumatic diseases with special emphasis on rheumatoid arthritis and spondyloarthritis. *Diagn Interv Radiol Ank Turk* 2014; 20: 72–77.

443. Rosenthal AK, Ryan LM. Nonpharmacologic and pharmacologic management of CPP crystal arthritis and BCP arthropathy and periarticular syndromes. *Rheum Dis Clin North Am* 2014; 40: 343–356.

444. Viana M, Sainaghi PP, Stecco A, et al. Headache in a patient with crowned dens: report of a new case. *Headache* 2014; 54: 1211–1216.

445. Wenham CYJ, Grainger AJ, Conaghan PG. The role of imaging modalities in the diagnosis, differential diagnosis and clinical assessment of peripheral joint osteoarthritis. *Osteoarthritis Cartilage* 2014; 22: 1692–1702.

446. Watura C, Saifuddin A. Tophaceous calcium pyrophosphate dihydrate deposition disease of the knee mimicking an aggressive soft tissue tumour. *BMJ Case Rep*; 2014. Epub ahead of print 10 June 2014. DOI: 10.1136/bcr-2014-203998.

447. Kahloune M, Libouton X, Omoumi P, et al. Osteoarthritis and scapholunate instability in chondrocalcinosis. *Diagn Interv Imaging* 2015; 96: 115–119.

448. Nakamura O, Kaji Y, Yamagami Y, et al. A Rare Case of Tumoral Calcium Pyrophosphate Dihydrate Crystal Deposition Disease of the Wrist Joint. *Case Rep Orthop* 2015; 2015: 313291.

449. Inokuchi R, Ohshima K, Yamamoto M, et al. Crowned dens syndrome. *Spine J Off J North Am Spine Soc* 2015; 15: 1499–1500.

450. Alix L, Guggenbuhl P. Familial hypocalciuric hypercalcemia associated with crystal deposition disease. *Joint Bone Spine* 2015; 82: 60–62.

451. Aouba A, Deshayes S, Frenzel L, et al. Efficacy of anakinra for various types of crystal-induced arthritis in complex hospitalized patients: a case series and review of the literature. *Mediators Inflamm* 2015; 2015: 792173.

452. Mori K, Imai S, Nishizawa K, et al. Cervical myelopathy due to calcification of the posterior atlantoaxial membrane associated with generalized articular deposition of calcium pyrophosphate dihydrate: a case report and review of the literature. *J Orthop Sci Off J Jpn Orthop Assoc* 2015; 20: 1136–1141.

453. Bartels CM, Singh JA, Parperis K, et al. Validation of administrative codes for calcium pyrophosphate deposition: a Veterans Administration study. *J Clin Rheumatol Pract Rep Rheum Musculoskelet Dis* 2015; 21: 189–192.

454. Boroda K, Chaudhry A, Clarke G, et al. An Unusual MRI Appearance of Osseous Metastases. *Cureus* 2015; 7: e300.

455. Borowski A, Heikaus S, Kurt M. Calcium Pyrophosphate Dihydrate Crystal Deposition Disease of the Sternoclavicular Joint. *Thorac Cardiovasc Surg Rep* 2015; 4: 46–48.

456. Koda R, Tsuchida Y, Yoshizawa K, et al. Crowned Dens Syndrome as an Initial Manifestation of Crystalline Deposition Disease. *Intern Med Tokyo Jpn* 2015; 54: 2405–2408.

457. Brennan KE, McGrory BJ. Monoarticular pseudogout of the hip: a case report. *Hosp Pract 1995* 2011; 39: 74–77.

458. Lomax A, Ferrero A, Cullen N, et al. Destructive pseudo-neuroarthropathy associated with calcium pyrophosphate deposition. *Foot Ankle Int* 2015; 36: 383–390.

459. Löffler C, Sattler H, Peters L, et al. Distinguishing gouty arthritis from calcium pyrophosphate disease and other arthritides. *J Rheumatol* 2015; 42: 513–520.

460. Chang WJ, Hamm B, Williams T, et al. Chronic Axial Neck Pain with Underlying Crowned Dens Syndrome. *Am J Phys Med Rehabil* 2015; 94: e128-129.

461. Checa A, Chun W. Rates of meniscal tearing in patients with chondrocalcinosis. *Clin Rheumatol* 2015; 34: 573–577.

462. Maravic M, Ea H-K. Hospital burden of gout, pseudogout and other crystal arthropathies in France. *Joint Bone Spine* 2015; 82: 326–329.

463. Miksanek J, Rosenthal AK. Imaging of calcium pyrophosphate deposition disease. *Curr Rheumatol Rep* 2015; 17: 20.

464. Cimbek EA, Şen Y, Yuca SA, et al. Chondrocalcinosis related to familial hypomagnesemia with hypercalciuria and nephrocalcinosis. *J Pediatr Endocrinol Metab JPEM* 2015; 28: 713–716.

465. Moses V, Parmar HA, Sawalha AH. Magnetic Resonance Imaging and Computed Tomography in the Evaluation of Crowned Dens Syndrome Secondary to Calcium Pyrophosphate Dihydrate. *J Clin Rheumatol Pract Rep Rheum Musculoskelet Dis* 2015; 21: 368–369.

466. Couturier M, Arbault A, Laroche D, et al. Impact of systematic ultrasound of the knee on the rheumatologist’s clinical decision in patients consulting for knee pain. *Rheumatol Int* 2016; 36: 283–288.

467. Kocyigit F, Kuyucu E, Kocyigit A. Monoarticular hip involvement in pseudogout. *Case Rep Rheumatol* 2015; 2015: 302389.

468. Laviv A, Sadow PM, Keith DA. Pseudogout in the temporomandibular joint with imaging, arthroscopic, operative, and pathologic findings. Report of an unusual case. *J Oral Maxillofac Surg Off J Am Assoc Oral Maxillofac Surg* 2015; 73: 1106–1112.

469. Di Carlo M, Draghessi A, Carotti M, et al. An Unusual Association: Iliopsoas Bursitis Related to Calcium Pyrophosphate Crystal Arthritis. *Case Rep Rheumatol* 2015; 2015: 935835.

470. Ochoa Escudero M, Juliano AF, Curtin HD. Inferior displacement of the lower belly of the lateral pterygoid muscle: a sign of temporomandibular joint lesions. *J Comput Assist Tomogr* 2015; 39: 340–342.

471. Oka A, Okazaki K, Takeno A, et al. Crowned Dens Syndrome: Report of Three Cases and a Review of the Literature. *J Emerg Med* 2015; 49: e9–e13.

472. Ottaviani S, Juge P-A, Aubrun A, et al. Sensitivity and Reproducibility of Ultrasonography in Calcium Pyrophosphate Crystal Deposition in Knee Cartilage: A Cross-sectional Study. *J Rheumatol* 2015; 42: 1511–1513.

473. Singh JA, Dalbeth N. Is the double contour sign specific for gout? Or only for crystal arthritis? *J Rheumatol* 2015; 42: 353–354.

474. Hong J-Y, Chang Y-S, Chou C-T, et al. Calcium Pyrophosphate Deposition Pseudoabscess. *J Clin Rheumatol Pract Rep Rheum Musculoskelet Dis* 2015; 21: 270.

475. Harato K, Yoshida H. Pseudogout at the knee joint will frequently occur after hip fracture and lead to the knee pain in the early postoperative period. *J Orthop Surg* 2015; 10: 4.

476. Grassi W, Okano T, Filippucci E. Use of ultrasound for diagnosis and monitoring of outcomes in crystal arthropathies. *Curr Opin Rheumatol* 2015; 27: 147–155.

477. Gamon E, Combe B, Barnetche T, et al. Diagnostic value of ultrasound in calcium pyrophosphate deposition disease: a systematic review and meta-analysis. *RMD Open* 2015; 1: e000118.

478. Pascual E, Andrés M, Sivera F. Methotrexate: should it still be considered for chronic calcium pyrophosphate crystal disease? *Arthritis Res Ther* 2015; 17: 89.

479. Patel T, Ryan L, Yu A. Crystal-induced delirium. *J Clin Rheumatol Pract Rep Rheum Musculoskelet Dis* 2015; 21: 108–109.

480. Pollet S, Coiffier G, Albert J-D, et al. Concordance between fresh joint fluid analysis by the rheumatologist and joint fluid analysis at the laboratory: Prospective single-center study of 180 samples. *Joint Bone Spine* 2015; 82: 161–165.

481. Ramonda R, Oliviero F, Galozzi P, et al. Molecular mechanisms of pain in crystal-induced arthritis. *Best Pract Res Clin Rheumatol* 2015; 29: 98–110.

482. Strub B, Von Campe A, Meuli-Simmen. The value of different inflammatory markers in distinguishing deep closed hand infections from non-infective causes. *J Hand Surg Eur Vol* 2015; 40: 207–208.

483. Tagoe CE, Raza Y. Acute crystal-induced arthritis and rheumatoid factor seropositivity. *Joint Bone Spine* 2015; 82: 135–136.

484. Takeda K, Minota S, Akiyama Y, et al. Pseudorheumatoid Arthritis Caused by Calcium Pyrophosphate Dihydrate Deposition. *Intern Med Tokyo Jpn* 2015; 54: 2957.

485. Taljanovic MS, Melville DM, Gimber LH, et al. High-Resolution US of Rheumatologic Diseases. *Radiogr Rev Publ Radiol Soc N Am Inc* 2015; 35: 2026–2048.

486. Ruban TN, Albert L. Wrist Involvement of Calcium Hydroxyapatite Deposition Disease. *J Rheumatol* 2015; 42: 1724–1725.

487. Zufferey P, Valcov R, Fabreguet I, et al. A prospective evaluation of ultrasound as a diagnostic tool in acute microcrystalline arthritis. *Arthritis Res Ther* 2015; 17: 188.

488. Zhang H, Jin D, Sun E. The early and late stages of crowned dens syndrome: two case reports. *Spine J Off J North Am Spine Soc* 2015; 15: e65-68.

489. Zaman M, Sabir N, Mills SP, et al. Pseudogout: A Rare Cause of Acute Arthritis Following Arthroscopic Anterior Cruciate Ligament Reconstruction. *Knee Surg Relat Res* 2015; 27: 194–196.

490. Yamada T, Saitoh T, Hozumi H, et al. Crowned dens syndrome. *Acute Med Surg* 2015; 2: 273.

491. Ward IM, Scott JN, Mansfield LT, et al. Dual-Energy Computed Tomography Demonstrating Destructive Calcium Pyrophosphate Deposition Disease of the Distal Radioulnar Joint Mimicking Tophaceous Gout. *J Clin Rheumatol Pract Rep Rheum Musculoskelet Dis* 2015; 21: 314–317.

492. Jungraithmayr W, Tzafos S, Distler O, et al. Rapid Growth of Lung Nodules due to Combined Pulmonary Vasculitis, Silicoanthracosis, and Chondrocalcinosis. *Can Respir J* 2016; 2016: 9254374.

493. Kobayashi T, Miyakoshi N, Abe T, et al. Acute neck pain caused by pseudogout attack of calcified cervical yellow ligament: a case report. *J Med Case Reports* 2016; 10: 133.

494. Rosen T, Furman J. Acute calcium pyrophosphate deposition arthropathy. *JAAPA Off J Am Acad Physician Assist* 2016; 29: 1–3.

495. Krochak R, Culbertson MD, Vigorita V, et al. Atypical Tumoral Presentation of Calcium Pyrophosphate Deposition Disease: A Case Report. *JBJS Case Connect* 2016; 6: e86.

496. Afzal W, Wali OM, Cervellione KL, et al. Coexistent Pseudogout and Mycobacterium avium-intracellulare Septic Arthritis in a Patient with HIV and ESRD. *Case Rep Rheumatol* 2016; 2016: 5495928.

497. Abhishek A, Doherty S, Maciewicz RA, et al. Does Chondrocalcinosis Associate With a Distinct Radiographic Phenotype of Osteoarthritis in Knees and Hips? A Case-Control Study. *Arthritis Care Res* 2016; 68: 211–216.

498. Abhishek A. Calcium pyrophosphate deposition disease: a review of epidemiologic findings. *Curr Opin Rheumatol* 2016; 28: 133–139.

499. Abhishek A, Doherty M. Update on calcium pyrophosphate deposition. *Clin Exp Rheumatol* 2016; 34: 32–38.

500. Iqbal Z, Mead P, Sayer JA. Case Report: Cervical chondrocalcinosis as a complication of Gitelman syndrome. *F1000Research* 2016; 5: 875.

501. Iqbal Z, Sayer JA. Chondrocalcinosis and Gitelman syndrome. *QJM Mon J Assoc Physicians* 2016; 109: 563–564.

502. Akkoç F, Tikiz C, Akgül Ö. Acute Pseudogout Arthritis in a Patient Using Diuretics. *Arch Rheumatol* 2016; 31: 297–298.

503. Hubert J, Hawellek T, Hischke S, et al. Hyaline cartilage calcification of the first metatarsophalangeal joint is associated with osteoarthritis but independent of age and BMI. *BMC Musculoskelet Disord* 2016; 17: 474.

504. Nakano H, Nakahara K, Michikawa Y, et al. Crowned dens syndrome developed after an endoscopic retrograde cholangiopancreatography procedure. *World J Gastroenterol* 2016; 22: 8849–8852.

505. Cammelli D, Vitiello G, Palterer B, et al. A case of acute febrile neck pain. *Intern Emerg Med* 2017; 12: 551–552.

506. Hoxha A, Ruffatti A, Alberioli E, et al. Erosive osteoarthritis, psoriatic arthritis and pseudogout; a casual association? *Clin Rheumatol* 2016; 35: 1885–1889.

507. Soloway S, Tucker BS. Calcium Pyrophosphate Dihydrate Deposition Disease in a Knee With Total Joint Replacement. *J Clin Rheumatol Pract Rep Rheum Musculoskelet Dis* 2016; 22: 277.

508. Kurihara M, Tokuda Y. Jolt accentuation of neck pain: a novel physical finding for crowned dens syndrome. *BMJ Case Rep*; 2016. Epub ahead of print 12 October 2016. DOI: 10.1136/bcr-2016-217128.

509. Dietvorst M, Roerdink R, Leenders ACAP, et al. Acute Mono-Arthritis of the Knee: A Case Report of Infection with Parvimonas Micra and Concomitant Pseudogout. *J Bone Jt Infect* 2016; 1: 65–67.

510. Cozzani E, Basso D, Cimmino MA, et al. Generalized annular granuloma associated with crowned dens syndrome, which resolved with colchicine treatment. *Clin Exp Dermatol* 2016; 41: 640–642.

511. Dadlani R, Dadlani R, Manam G, et al. Unilateral spontaneous ankylosis of the C1/C2 joint with ‘crowned dens syndrome’: Computed tomography scan is imperative in diagnosis. *J Neurosci Rural Pract* 2016; 7: 594–595.

512. Ng IB-Y, Arkun K, Riesenburger RI. Posterior C1-C2 calcium pyrophosphate dihydrate crystal deposition disease. *BMJ Case Rep*; 2016. Epub ahead of print 14 March 2016. DOI: 10.1136/bcr-2016-214771.

513. Omoumi P, Zufferey P, Malghem J, et al. Imaging in Gout and Other Crystal-Related Arthropathies. *Rheum Dis Clin North Am* 2016; 42: 621–644.

514. Pappu R, Jabbour SA, Reginato AM, et al. Musculoskeletal manifestations of primary hyperparathyroidism. *Clin Rheumatol* 2016; 35: 3081–3087.

515. Park HJ, Chung HW, Oh TS, et al. Tumoral pseudogout of the proximal interphalangeal joint of a finger: a case report and literature review. *Skeletal Radiol* 2016; 45: 1007–1012.

516. Sivera F, Andrés M, Pascual E. Current advances in therapies for calcium pyrophosphate crystal arthritis. *Curr Opin Rheumatol* 2016; 28: 140–144.

517. Horino T, Matsumoto T, Terada Y, et al. Lipoma arborescens in a patient with pseudogout on hemodialysis. *QJM Mon J Assoc Physicians* 2016; 109: 827–828.

518. Higgins PA. Gout and pseudogout. *JAAPA Off J Am Acad Physician Assist* 2016; 29: 50–52.

519. Gersing AS, Schwaiger BJ, Heilmeier U, et al. Evaluation of Chondrocalcinosis and Associated Knee Joint Degeneration Using MR Imaging: Data from the Osteoarthritis Initiative. *Eur Radiol* 2017; 27: 2497–2506.

520. Glanville JRW, Higgens C, Mouyis M. An approach to joint pain and inflammatory arthropathies. *Br J Hosp Med Lond Engl 2005* 2016; 77: C109-111.

521. Galed-Placed I, Hernández-Cancela R, Vázquez-Bueno JÁ. Coincidence of monosodium urate monohydrate, calcium pyrophosphate dihydrate, and basic calcium phosphate crystals in the synovial fluid from a single joint. *Scand J Rheumatol* 2016; 45: 167–169.

522. Galozzi P, Oliviero F, Frallonardo P, et al. The prevalence of monosodium urate and calcium pyrophosphate crystals in synovial fluid from wrist and finger joints. *Rheumatol Int* 2016; 36: 443–446.

523. Fung CS, Tam GK. Crowned dens syndrome: an uncommon cause of cord compression. *Hong Kong Med J Xianggang Yi Xue Za Zhi* 2016; 22: 399.e4–5.

524. Frallonardo P, Oliviero F, Peruzzo L, et al. Detection of Calcium Crystals in Knee Osteoarthritis Synovial Fluid: A Comparison Between Polarized Light and Scanning Electron Microscopy. *J Clin Rheumatol Pract Rep Rheum Musculoskelet Dis* 2016; 22: 369–371.

525. Filippou G, Adinolfi A, Iagnocco A, et al. Ultrasound in the diagnosis of calcium pyrophosphate dihydrate deposition disease. A systematic literature review and a meta-analysis. *Osteoarthritis Cartilage* 2016; 24: 973–981.

526. Filippou G, Tacchini D, Adinolfi A, et al. Histology of the synovial membrane of patients affected by osteoarthritis and calcium pyrophosphate dihydrate crystal deposition disease vs. osteoarthritis alone: a pilot study. *Scand J Rheumatol* 2016; 45: 538–539.

527. Filippou G, Adinolfi A, Cimmino MA, et al. Diagnostic accuracy of ultrasound, conventional radiography and synovial fluid analysis in the diagnosis of calcium pyrophosphate dihydrate crystal deposition disease. *Clin Exp Rheumatol* 2016; 34: 254–260.

528. Escrivá-Fornés M, González-Puig L, Román-Ivorra JA, et al. Pseudogout in a patient with bilateral total knee prosthesis: A challenging diagnosis. *Joint Bone Spine* 2016; 83: 463–464.

529. Patel T, Ryan L, Dubois M, et al. The prevalence of chondrocalcinosis of the symphysis pubis on CT scan and correlation with calcium pyrophosphate dihydrate crystal deposition disease. *Clin Rheumatol* 2016; 35: 771–773.

530. Pawelek SA, Illes JD, Taylor JA. Calcium Pyrophosphate Dihydrate Crystal Deposition Disease Simulating Osteoarthrosis of the Knee: A Case Report. *J Chiropr Med* 2016; 15: 219–223.

531. Stensby JD, Lawrence DA, Patrie JT, et al. Prevalence of asymptomatic chondrocalcinosis in the pelvis. *Skeletal Radiol* 2016; 45: 949–954.

532. Tagami S, Inokuchi R, Awaji K, et al. Crowned dens syndrome and interspinous ligament inflammation due to calcium pyrophosphate deposition in an elderly man. *Spine J Off J North Am Spine Soc* 2016; 16: e453-454.

533. Tajima K, Ueda T, Ueno K, et al. Crowned dens syndrome: reports of six cases and review of the literature. *Acute Med Surg* 2016; 3: 155–158.

534. Rheinboldt M, Scher C. Musculoskeletal ultrasonography in the diagnosis of acute crystalline synovitis. *Emerg Radiol* 2016; 23: 623–632.

535. Ryosuke, Takasago T, Nakamura M, et al. Chondrogenesis in the synovial tissue is associated with the onset of pseudogout after total knee arthroplasty. *Arthroplasty Today* 2016; 2: 101–104.

536. Ruta S, Catay E, Marin J, et al. Knee effusion: ultrasound as a useful tool for the detection of calcium pyrophosphate crystals. *Clin Rheumatol* 2016; 35: 1087–1091.

537. Rosenthal AK, Ryan LM. Calcium Pyrophosphate Deposition Disease. *N Engl J Med* 2016; 374: 2575–2584.

538. Zabotti A, Della Siega P, Picco L, et al. Gitelman syndrome disclosed by calcium pyrophosphate deposition disease: early diagnosis by ultrasonographic study. *Reumatismo* 2016; 68: 53–55.

539. Verhoeven F, Chouk M, Wendling D. Ultrasonography: A Useful Tool for the Diagnosis of Chondrocalcinosis of the Sternoclavicular Joint. *J Rheumatol* 2016; 43: 1148.

540. Yahia SA, Zeller V, Desplaces N, et al. Crystal-induced arthritis after arthroplasty: 7 cases. *Joint Bone Spine* 2016; 83: 559–562.

541. Wu Y, Chen K, Terkeltaub R. Systematic review and quality analysis of emerging diagnostic measures for calcium pyrophosphate crystal deposition disease. *RMD Open* 2016; 2: e000339.

542. Wadhwa V, Cho G, Moore D, et al. T2 black lesions on routine knee MRI: differential considerations. *Eur Radiol* 2016; 26: 2387–2399.

543. Joshi A, Siva C. Magnesium disorders can cause calcium pyrophosphate deposition disease: A case report and literature review. *Eur J Rheumatol* 2018; 5: 53–57.

544. Kudoh K, Kudoh T, Tsuru K, et al. A case of tophaceous pseudogout of the temporomandibular joint extending to the base of the skull. *Int J Oral Maxillofac Surg* 2017; 46: 355–359.

545. Lim CH, Lin C-T, Chen Y-H. Acute calcific tendinitis of gluteus maximus tendon due to tumoral calcium pyrophosphate dihydrate deposition disease. *Int J Rheum Dis* 2017; 20: 2249–2252.

546. Jiang M, Navanathan S. Crowned dens syndrome: a rare cause of neck pain and fever. *Med J Aust* 2017; 206: 199.

547. Jens S, Luijkx T, Smithuis FF, et al. Diagnostic modalities for distal radioulnar joint. *J Hand Surg Eur Vol* 2017; 42: 395–404.

548. Jacques T, Michelin P, Badr S, et al. Conventional Radiology in Crystal Arthritis: Gout, Calcium Pyrophosphate Deposition, and Basic Calcium Phosphate Crystals. *Radiol Clin North Am* 2017; 55: 967–984.

549. Jacobson JA, Roberts CC, Bencardino JT, et al. ACR Appropriateness Criteria(®) Chronic Extremity Joint Pain-Suspected Inflammatory Arthritis. *J Am Coll Radiol JACR* 2017; 14: S81–S89.

550. Imamura T, Lotterman S, Glazer C. Elderly Male With Neck Stiffness and Fever. *Ann Emerg Med* 2017; 69: 665–673.

551. Inoue A, Kohno K, Ninomiya S, et al. Usefulness of cervical computed tomography and magnetic resonance imaging for rapid diagnosis of crowned dens syndrome: A case report and review of the literature. *Int J Surg Case Rep* 2017; 30: 50–54.

552. Ankli B, Kyburz D, Hirschmann A, et al. Calcium pyrophosphate deposition disease: a frequent finding in patients with long-standing erosive gout. *Scand J Rheumatol* 2018; 47: 127–130.

553. Kleiber Balderrama C, Rosenthal AK, Lans D, et al. Calcium Pyrophosphate Deposition Disease and Associated Medical Comorbidities: A National Cross-Sectional Study of US Veterans. *Arthritis Care Res* 2017; 69: 1400–1406.

554. Awan R, Ghazanfar H, Martes Pena KA, et al. An Unusual Case of Acute Chondrocalcinosis in Wrist Joint Presenting as Cellulitis. *Cureus* 2017; 9: e1916.

555. Baudart P, Molin A, Cesini J, et al. Calcium pyrophosphate deposition disease revealing a hypersensitivity to vitamin D. *Joint Bone Spine* 2017; 84: 349–351.

556. Berendsen D, Neogi T, Taylor WJ, et al. Crystal identification of synovial fluid aspiration by polarized light microscopy. An online test suggesting that our traditional rheumatologic competence needs renewed attention and training. *Clin Rheumatol* 2017; 36: 641–647.

557. Boumans D, Hettema ME, Vonkeman HE, et al. The added value of synovial fluid centrifugation for monosodium urate and calcium pyrophosphate crystal detection. *Clin Rheumatol* 2017; 36: 1599–1605.

558. Kohno N, Kobori Y, Yamaguchi S. Crowned Dens Syndrome Associated with Bowel Cleaning for Colonoscopy. *Intern Med Tokyo Jpn* 2017; 56: 2645–2647.

559. Bridges KJ, Bullis CL, Wanchu A, et al. Pseudogout of the cervical and thoracic spine mimicking infection after lumbar fusion: case report. *J Neurosurg Spine* 2017; 27: 145–149.

560. Buckens CF, Terra MP, Maas M. Computed Tomography and MR Imaging in Crystalline-Induced Arthropathies. *Radiol Clin North Am* 2017; 55: 1023–1034.

561. Bui PV, Tuan NM, Nghia HTN, et al. Mineral and Bone Disorder in Chronic Kidney Disease: A Case Report from Vietnam. *Blood Purif* 2017; 44 Suppl 1: 46–51.

562. Can B, Kara M, Kara Ö, et al. The value of musculoskeletal ultrasound in geriatric care and rehabilitation. *Int J Rehabil Res Int Z Rehabil Rev Int Rech Readaptation* 2017; 40: 285–296.

563. Carlson AK, McCutchen CN, June RK. Mechanobiological implications of articular cartilage crystals. *Curr Opin Rheumatol* 2017; 29: 157–162.

564. Priesand S, Wyckoff J, Wrobel J, et al. Acute pseudogout of the foot following Parathyroidectomy: a case report. *Clin Diabetes Endocrinol* 2017; 3: 10.

565. Carpenter L, Juliano N, Herb R. Recurrent Painful Calcium Pyrophosphate Arthropathy. *J Am Osteopath Assoc* 2017; 117: 199.

566. Castro AM, Saraiva F, Polido Pereira J, et al. Calcium Pyrophosphate Dihydrate Deposition in a Pseudarthrosis: A New Location for an Old Disease. *J Clin Rheumatol Pract Rep Rheum Musculoskelet Dis* 2017; 23: 176–178.

567. Chen K-C, Chu P. The Case | Hypomagnesemia with knee pain. *2017* 2017; 91: 1261–1262.

568. Couto AR, Parreira B, Thomson R, et al. Combined approach for finding susceptibility genes in DISH/chondrocalcinosis families: whole-genome-wide linkage and IBS/IBD studies. *Hum Genome Var* 2017; 4: 17041.

569. Couture G, Delzor F, Bagheri H, et al. First cases of calcium pyrophosphate deposition disease after zoledronic acid therapy. *Joint Bone Spine* 2017; 84: 213–215.

570. Di Matteo A, Filippucci E, Salaffi F, et al. Diagnostic accuracy of musculoskeletal ultrasound and conventional radiography in the assessment of the wrist triangular fibrocartilage complex in patients with definite diagnosis of calcium pyrophosphate dihydrate deposition disease. *Clin Exp Rheumatol* 2017; 35: 647–652.

571. Karimzadeh H, Sirous M, Sadati SN, et al. Prevalence of Chondrocalcinosis in Patients above 50 Years and the Relationship with Osteoarthritis. *Adv Biomed Res* 2017; 6: 98.

572. Singh N, Vogelgesang SA. Monoarticular Arthritis. *Med Clin North Am* 2017; 101: 607–613.

573. Han BK, Kim W, Niu J, et al. Association of Chondrocalcinosis in Knee Joints With Pain and Synovitis: Data From the Osteoarthritis Initiative. *Arthritis Care Res* 2017; 69: 1651–1658.

574. Ferreyra M, Coiffier G, Albert J-D, et al. Combining cytology and microcrystal detection in nonpurulent joint fluid benefits the diagnosis of septic arthritis. *Joint Bone Spine* 2017; 84: 65–70.

575. Filippou G, Scirè CA, Damjanov N, et al. Definition and Reliability Assessment of Elementary Ultrasonographic Findings in Calcium Pyrophosphate Deposition Disease: A Study by the OMERACT Calcium Pyrophosphate Deposition Disease Ultrasound Subtask Force. *J Rheumatol* 2017; 44: 1744–1749.

576. Shikino K, Ota T, Ikusaka M. Crowned Dens Syndrome. *Am J Med* 2017; 130: e111–e112.

577. Shapiro JR, Lewiecki EM. Hypophosphatasia in Adults: Clinical Assessment and Treatment Considerations. *J Bone Miner Res Off J Am Soc Bone Miner Res* 2017; 32: 1977–1980.

578. Petit H, Marcellin L, Chatelus E. Lumbar Spine Chondrocalcinosis. *J Rheumatol* 2017; 44: 1288–1289.

579. Stainsby BE, Ellul N, Dimopoulos A, et al. Calcium pyrophosphate deposition disease in the ankle joint: a case report. *J Can Chiropr Assoc* 2017; 61: 40–44.

580. Takahashi T, Tamura M, Takasu T, et al. Clinical and quantitative analysis of patients with crowned dens syndrome. *J Neurol Sci* 2017; 376: 52–59.

581. Roddy E, Muller S, Paskins Z, et al. Incident acute pseudogout and prior bisphosphonate use: Matched case-control study in the UK-Clinical Practice Research Datalink. *Medicine (Baltimore)* 2017; 96: e6177.

582. Tedeschi SK, Stone RM, Helfgott SM. Calcium Pyrophosphate Crystal Inflammatory Arthritis (Pseudogout) with Myelodysplastic Syndrome: A New Paraneoplastic Syndrome? *J Rheumatol* 2017; 44: 1101–1102.

583. Testa EJ, McGrory BJ. Adverse reaction to metal debris with concomitant incidental crystalline arthropathy in hip arthroplasty. *Arthroplasty Today* 2017; 3: 19–23.

584. Rothschild B. Correlation of Periodontal Disease With Inflammatory Arthritis in the Time Before Modern Medical Intervention. *J Periodontol* 2017; 88: 266–272.

585. Zeng C, Wei J, Terkeltaub R, et al. Dose-response relationship between lower serum magnesium level and higher prevalence of knee chondrocalcinosis. *Arthritis Res Ther* 2017; 19: 236.

586. Younis AA-R. Crowned Dens Syndrome as a cause of acute neck pain: a Case Report and Review of the Literature. *Mediterr J Rheumatol* 2017; 28: 101–105.

587. Villion A, Arinzon Z, Feldman J, et al. Crystal-Induced Arthropathy in Elderly Patients Hospitalized for Acute Conditions. *Isr Med Assoc J IMAJ* 2017; 19: 183–185.

588. Wickrematilake GW. Calcium Pyrophosphate Dihydrate Deposition Disease in Young Patients: Two Case Reports. *Arch Rheumatol* 2017; 32: 80–83.

589. Watanabe K, Yamazaki Y, Sugawara M. Tuberculosis of the Wrist Accompanied with Calcium Pyrophosphate. *Intern Med Tokyo Jpn* 2017; 56: 3389–3394.

590. Kobayashi T, Miyakoshi N, Konno N, et al. Age-Related Prevalence of Periodontoid Calcification and Its Associations with Acute Cervical Pain. *Asian Spine J* 2018; 12: 1117–1122.

591. Muangchan C, Bhurihirun T. An investigation of the independent risk factors that differentiate gout from pseudogout in patients with crystal-induced acute arthritis: a cross-sectional study. *Rheumatol Int* 2018; 38: 89–95.

592. Masmoudi K, Elleuch E, Akrout R, et al. Bilateral septic arthritis of the sternoclavicular joint complicating infective endocarditis: a case report. *J Med Case Reports* 2018; 12: 205.

593. McCarthy GM, Dunne A. Calcium crystal deposition diseases - beyond gout. *Nat Rev Rheumatol* 2018; 14: 592–602.

594. Abhishek A, Neogi T, Choi H, et al. Review: Unmet Needs and the Path Forward in Joint Disease Associated With Calcium Pyrophosphate Crystal Deposition. *Arthritis Rheumatol Hoboken NJ* 2018; 70: 1182–1191.

595. Abhishek A, Iagnocco A, Bijlsma JWJ, et al. Cross-sectional survey of the undergraduate rheumatology curriculum in European medical schools: a EULAR School of Rheumatology initiative. *RMD Open* 2018; 4: e000743.

596. Hubert J, Weiser L, Hischke S, et al. Cartilage calcification of the ankle joint is associated with osteoarthritis in the general population. *BMC Musculoskelet Disord* 2018; 19: 169.

597. Andrés M, Sivera F, Pascual E. Therapy for CPPD: Options and Evidence. *Curr Rheumatol Rep* 2018; 20: 31.

598. Argyropoulos M, Iyengar KP, Suraliwala KH. First Presentation of Acute Pseudogout Following Total Knee Replacement. *J Orthop Case Rep* 2018; 8: 32–34.

599. Martens HA, van Bokhoven SC, Stenger AAME. Calcium pyrophosphate deposition disease induced sacroiliitis. *Rheumatol Oxf Engl*. Epub ahead of print 26 March 2018. DOI: 10.1093/rheumatology/key098.

600. Kwon K-J, Seok H, Lee J-H, et al. Calcium pyrophosphate dihydrate deposition disease in the temporomandibular joint: diagnosis and treatment. *Maxillofac Plast Reconstr Surg* 2018; 40: 19.

601. Ledingham D, Cappelen-Smith C, Cordato D. Crowned dens syndrome. *Pract Neurol* 2018; 18: 57–59.

602. Pavic K, Pandya J, Sebak S, et al. Acute arthritis: predictive factors and current practice in the approach to diagnosis and management across two hospitals in Sydney. *Intern Med J* 2018; 48: 1087–1095.

603. Chang I, Gazeley D. Crystalline arthropathy and bone health. *Curr Opin Rheumatol* 2018; 30: 173–176.

604. Chiba D, Tsuda E, Sasaki E, et al. Low prevalence of knee chondrocalcinosis and its catabolic association with serum matrix metalloproteinase 3: A rural Japanese population study. *Int J Rheum Dis* 2018; 21: 2011–2018.

605. Cho NH, Song Y, Lee S, et al. Incidence of knee chondrocalcinosis and its risk factors in a community-based cohort. *Int J Rheum Dis* 2018; 21: 1391–1397.

606. Colaco C, Dotel R. Coexisting polyarticular septic arthritis, gout and pseudogout. *BMJ Case Rep* 2018; 2018: bcr-2018-226810.

607. Conway R, McCarthy GM. Calcium-Containing Crystals and Osteoarthritis: an Unhealthy Alliance. *Curr Rheumatol Rep* 2018; 20: 13.

608. Ottaviani S, Forien M, Dieudé P. A Radiographic Double Contour Sign Mimicking Pseudogout. *J Clin Rheumatol Pract Rep Rheum Musculoskelet Dis* 2018; 24: 92.

609. Coutier F, Sondag M, Wendling D. Coexisting Gout and Chondrocalcinosis on Hand Radiograph. *J Rheumatol* 2018; 45: 723–724.

610. Curzi D, Fardetti F, Beccarini A, et al. Chondroptotic chondrocytes in the loaded area of chondrocalcinotic cartilage: A clinical proposal? *Clin Anat N Y N* 2018; 31: 1188–1192.

611. Lans J, Machol JA 4th, Deml C, et al. Nonrheumatoid Arthritis of the Hand. *J Hand Surg* 2018; 43: 61–67.

612. Mijola L, Amouzougan A, Barral F-G, et al. Retro-odontoid pseudo-tumor due to calcium pyrophosphate crystal deposits with spinal cord compression and histopathological confirmation. *Joint Bone Spine* 2018; 85: 497–498.

613. Madhavan K, Chieng LO, Gaynor BG, et al. Transdural approach to resection of retro-odontoid cysts in elderly patients: report of 3 cases. *J Neurosurg Spine* 2018; 28: 236–243.

614. Delpont B, Blanc C, Osseby GV, et al. Pain after stroke: A review. *Rev Neurol (Paris)* 2018; 174: 671–674.

615. Heck A, Nolan N, Rojas-Moreno C. Crowned Dens Syndrome: Calcium Pyrophosphate Deposition Disease Masquerading as Osteomyelitis. *J Rheumatol* 2018; 45: 1422–1423.

616. Hatayama M, Ikuta K, Ishioh M, et al. Pseudogout Attack after Pegfilgrastim Administration in Anaplastic Large Cell Lymphoma. *Intern Med Tokyo Jpn* 2018; 57: 1779–1782.

617. Goel P, Premnath N, Agrawal M, et al. Unusual cause of neck pain. *BMJ Case Rep* 2018; 2018: bcr-2018-224183.

618. Grana E, Da Costa F de Frias Pinto A, Carda S. Acute pseudogout after cinacalcet treatment. *Kidney Int* 2018; 94: 831–832.

619. Fuentes-Martinez N, Tani E, Darai-Ramqvist E, et al. Case report: Calcium pyrophosphate dihydrate deposition of the temporomandibular joint diagnosed by fine-needle aspiration cytology. *Diagn Cytopathol* 2018; 46: 610–612.

620. Freire V, Moser TP, Lepage-Saucier M. Radiological identification and analysis of soft tissue musculoskeletal calcifications. *Insights Imaging* 2018; 9: 477–492.

621. Sidari A, Hill E. Diagnosis and Treatment of Gout and Pseudogout for Everyday Practice. *Prim Care* 2018; 45: 213–236.

622. Forien M, Combier A, Gardette A, et al. Comparison of ultrasonography and radiography of the wrist for diagnosis of calcium pyrophosphate deposition. *Joint Bone Spine* 2018; 85: 615–618.

623. Frallonardo P, Ramonda R, Peruzzo L, et al. Basic calcium phosphate and pyrophosphate crystals in early and late osteoarthritis: relationship with clinical indices and inflammation. *Clin Rheumatol* 2018; 37: 2847–2853.

624. Filippou G, Scirè CA, Adinolfi A, et al. Identification of calcium pyrophosphate deposition disease (CPPD) by ultrasound: reliability of the OMERACT definitions in an extended set of joints-an international multiobserver study by the OMERACT Calcium Pyrophosphate Deposition Disease Ultrasound Subtask Force. *Ann Rheum Dis* 2018; 77: 1194–1199.

625. Sano M, Yamashita S, Aiba T. The prevalence of calcification around odontoid process and the incidence of crowned dens syndrome in the neurosurgical ward: A single institution’s analysis. *Mod Rheumatol* 2018; 28: 182–187.

626. Subramanian H, Gochhait D, Ganesh RN, et al. Diagnosis of pseudo-gout (calcium pyrophosphate deposition disease) clinched on cytology. *Diagn Cytopathol* 2018; 46: 748–751.

627. Tai CH, Oh HB, Seet JE, et al. Pseudogout - a rare manifestation of hungry bone syndrome after focused parathyroidectomy. *Ann R Coll Surg Engl* 2018; 100: e106–e108.

628. Schlee S, Bollheimer LC, Bertsch T, et al. Crystal arthritides - gout and calcium pyrophosphate arthritis : Part 1: Epidemiology and pathophysiology. *Z Gerontol Geriatr* 2018; 51: 453–460.

629. Rigsbee CA, Sizemore TC, Lohr KM. Severe calcium pyrophosphate dihydrate deposition disease of the metacarpophalangeal joints. *BMJ Case Rep* 2018; 2018: bcr-2018-226132.

630. Takahashi T, Hanakita J, Minami M. Pathophysiology of Calcification and Ossification of the Ligamentum Flavum in the Cervical Spine. *Neurosurg Clin N Am* 2018; 29: 47–54.

631. Tanikawa H, Ogawa R, Okuma K, et al. Detection of calcium pyrophosphate dihydrate crystals in knee meniscus by dual-energy computed tomography. *J Orthop Surg* 2018; 13: 73.

632. Tedeschi SK, Solomon DH, Liao KP. Pseudogout among Patients Fulfilling a Billing Code Algorithm for Calcium Pyrophosphate Deposition Disease. *Rheumatol Int* 2018; 38: 1083–1088.

633. Sabchyshyn V, Konon I, Ryan LM, et al. Concurrence of rheumatoid arthritis and calcium pyrophosphate deposition disease: A case collection and review of the literature. *Semin Arthritis Rheum* 2018; 48: 9–11.

634. Rothschild BM. Differential diagnostic perspectives provided by en face microscopic examination of articular surface defects. *Clin Rheumatol* 2018; 37: 831–836.

635. Zamudio-Cuevas Y, Fernández-Torres J, Martínez-Nava GA, et al. Emergent nanotherapies in microcrystal-induced arthritis. *Int Immunopharmacol* 2018; 61: 197–203.

636. Yamamura M. Acute CPP Crystal Arthritis Causing Carpal Tunnel Syndrome. *Intern Med Tokyo Jpn* 2018; 57: 2767–2768.

637. Vele P, Simon S-P, Damian L, et al. Clinical and ultrasound findings in patients with calcium pyrophosphate dihydrate deposition disease. *Med Ultrason* 2018; 20: 159–163.

638. Vellone V, Bracciolini V, Ramieri V, et al. Synovial Chondromatosis and Calcium Pyrophosphate Deposition of the Temporomandibular Joint: Challenging Diagnosis. *J Craniofac Surg* 2018; 29: e792–e794.

639. Wu OC, Atli K, Kasliwal MK. Images in neuroscience: Cervical bony lesion. *J Clin Neurosci Off J Neurosurg Soc Australas* 2018; S0967-5868(18)30858–0.

640. Wold A, Petscavage-Thomas J, Walker EA. Non-union rate of type II and III odontoid fractures in CPPD versus a control population. *Skeletal Radiol* 2018; 47: 1499–1504.

641. Wada N, Yamashita K, Hiwatashi A, et al. Calcium pyrophosphate dihydrate crystal deposition disease of the spinal dura mater: a case report. *BJR Case Rep* 2018; 4: 20170049.

642. Shen G, Su M, Liu B, et al. A Case of Tophaceous Pseudogout on 18F-FDG PET/CT Imaging. *Clin Nucl Med* 2019; 44: e98–e100.

643. Kaffel D, Maatallah K, Ferjani H, et al. Clinical Images: Pseudogout of the sternoclavicular joint. *ACR Open Rheumatol* 2019; 1: 365.

644. Joyce AA, Williams JN, Shi J, et al. Atlanto-axial Pannus in Patients with and without Rheumatoid Arthritis. *J Rheumatol* 2019; 46: 1431–1437.

645. Jeong JH, Jung JH, Lee JS, et al. Prominent Inflammatory Features of Monocytes/Macrophages in Acute Calcium Pyrophosphate Crystal Arthritis: a Comparison with Acute Gouty Arthritis. *Immune Netw* 2019; 19: e21.

646. Iwasaki K, Nakamura T, Shin S, et al. Calcium pyrophosphate deposition disease after total knee arthroplasty: Comparison with periprosthetic joint infection. *J Orthop* 2019; 16: 128–132.

647. Iqbal SM, Qadir S, Aslam HM, et al. Updated Treatment for Calcium Pyrophosphate Deposition Disease: An Insight. *Cureus* 2019; 11: e3840.

648. Huang Y-J, Kuo C-F. Can drugs trigger CPPD acute attacks? *Joint Bone Spine* 2019; 86: 131–134.

649. Amouzougan A, Vassal F, Peoc’h M, et al. Calcium Pyrophosphate Deposition Disease Arthropathy-Related Sciatica. *Arthritis Rheumatol Hoboken NJ* 2019; 71: 2099.

650. Andrés M, Vela P, Jovaní V, et al. Most needle-shaped calcium pyrophosphate crystals lack birefringence. *Rheumatol Oxf Engl* 2019; 58: 1095–1098.

651. Ariyawatkul T, Pichaisak W, Chavasiri C, et al. The Role of Calcium Pyrophosphate Dihydrate Deposition in the Postoperative Outcome of Lumbar Spinal Stenosis Patients. *Asian Spine J* 2019; 13: 1001–1009.

652. Lim CH, Ng BH, Teh HL. Calcium pyrophosphate dihydrate deposition disease: a forgotten common arthritis in the elderly. *BMJ Case Rep*; 12. Epub ahead of print 21 November 2019. DOI: 10.1136/bcr-2019-232828.

653. Baillet A, Trocmé C, Romand X, et al. Calprotectin discriminates septic arthritis from pseudogout and rheumatoid arthritis. *Rheumatol Oxf Engl* 2019; 58: 1644–1648.

654. Bangert E, Hofkirchner A, Towheed TE. Concomitant Parvimonas micra Septic Arthritis and Pseudogout After Total Knee Arthroplasty. *J Clin Rheumatol Pract Rep Rheum Musculoskelet Dis* 2019; 25: 47–50.

655. Bansal A, Gupta M. Crowned dens syndrome presenting as pyrexia of unknown origin (PUO). *Romanian J Intern Med Rev Roum Med Interne* 2019; 57: 266–269.

656. Becce F. Diagnosis of calcium pyrophosphate deposition by imaging - current state and challenges remaining. *Osteoarthritis Cartilage* 2019; 27: 545–546.

657. Becce F, Viry A, Stamp LK, et al. Winds of change in imaging of calcium crystal deposition diseases. *Joint Bone Spine* 2019; 86: 665–668.

658. Lee K-A, Lee S-H, Kim H-R. Diagnostic value of ultrasound in calcium pyrophosphate deposition disease of the knee joint. *Osteoarthritis Cartilage* 2019; 27: 781–787.

659. Soma T, Asoda S, Kimura M, et al. Acute odontogenic infection combined with crowned dens syndrome: a case report. *J Med Case Reports* 2019; 13: 143.

660. Sousa APD, Moura C, da Hora PR, et al. Calcium crystal-associated arthropathy mimicking a febrile systemic inflammatory disease in an elderly patient. *Oxf Med Case Rep* 2019; 2019: omz030.

661. Sadiq M, Nayak M, Farheen A, et al. An Unusual Case of Huge Tophaceous Pseudogout Mimicking as a Tumor-Like Lesion around the Ankle Joint: A Case Report and Literature Review. *Case Rep Orthop* 2019; 2019: 9617184.

662. Chouk M, Verhoeven F, Sondag M, et al. Value of serum procalcitonin for the diagnosis of bacterial septic arthritis in daily practice in rheumatology. *Clin Rheumatol* 2019; 38: 2265–2273.

663. Cooper R, Dudley J, Farmakiotis D. A Man With Headache, Fever, and Neck Stiffness. *JAMA* 2019; 321: 1624–1625.

664. Rothschild BM. A Rose by Any Other Name: Classified Accelerated Erosive Osteoarthritis or Calcium Pyrophosphate Deposition Disease, a Clarion for Aggressive Intervention. *J Rheumatol* 2019; 46: 867.

665. Khmelinskii N, Polido-Pereira J. Mixed crystal arthropathy of the wrist: The contribution of imaging in the diagnostic approach. *Reumatol Clin* 2019; 15: e140–e141.

666. Di Matteo A, Filippucci E, Cipolletta E, et al. Hip Involvement in Patients With Calcium Pyrophosphate Deposition Disease: Potential and Limits of Musculoskeletal Ultrasound. *Arthritis Care Res* 2019; 71: 1671–1677.

667. Maloney KD, Balakumar A, McGann S, et al. Pseudogout Mimicking Aortic Dissection: A Case Report. *Cureus* 2019; 11: e6239.

668. Kim ST, Bittar M, Kim HJ, et al. Recurrent pseudogout after therapy with immune checkpoint inhibitors: a case report with immunoprofiling of synovial fluid at each flare. *J Immunother Cancer* 2019; 7: 126.

669. Northrup EN, Pflederer BR. Calcium pyrophosphate dihydrate crystal deposition disease and MRSA septic arthritis of the atlantoaxial joint in a patient with Tourette syndrome. *BMJ Case Rep*; 12. Epub ahead of print 22 March 2019. DOI: 10.1136/bcr-2018-228102.

670. Moshrif A, Laredo JD, Bassiouni H, et al. Spinal involvement with calcium pyrophosphate deposition disease in an academic rheumatology center: A series of 37 patients. *Semin Arthritis Rheum* 2019; 48: 1113–1126.

671. Mohammed RHA, Kotb H, Amir M, et al. Subclinical crystal arthropathy: a silent contributor to inflammation and functional disability in knees with osteoarthritis-an ultrasound study. *J Med Ultrason 2001* 2019; 46: 137–146.

672. Mizokami F, Takahashi Y, Isogai Z. Two cases of pressure ulcers related to acute calcium pyrophosphate crystal arthritis: A new concept of ‘disease-specific unexpected external forces’. *Int Wound J* 2019; 16: 556–558.

673. Loizidis G, Stern J, Baker JF. When Calcium Pyrophosphate Deposition Disease Masquerades as Spinal Infection. *J Clin Rheumatol Pract Rep Rheum Musculoskelet Dis* 2019; 25: e118–e122.

674. De Jong M, Candanedo C, Keidar Haran T, et al. A Curious Case of Crystal Deposit Disease in the Petrous Bone. *Cureus* 2019; 11: e6375.

675. De la Garza-Montaño P, Pineda C, Lozada-Pérez CA, et al. Prevalence of chondrocalcinosis in a Mexican tertiary care institution of musculoskeletal disorders. *Clin Rheumatol* 2019; 38: 2595–2602.

676. Paalanen K, Rannio K, Rannio T, et al. Does early seronegative arthritis develop into rheumatoid arthritis? A 10-year observational study. *Clin Exp Rheumatol* 2019; 37: 37–43.

677. Slostad JA, Wild EM, Anderson CM, et al. Intractable Neck Pain in a Patient With Newly Diagnosed AML: An Underrecognized Cause of a Treatable Syndrome. *J Pain Symptom Manage* 2019; 57: e3–e5.

678. Hosu CD, Moisoiu V, Stefancu A, et al. Raman spectroscopy applications in rheumatology. *Lasers Med Sci* 2019; 34: 827–834.

679. Ho K-Y, Liang JN. Calcium Pyrophosphate Deposition Disease in the Achilles Tendon. *J Belg Soc Radiol* 2019; 103: 79.

680. Hameed M, Turkiewicz A, Englund M, et al. Prevalence and incidence of non-gout crystal arthropathy in southern Sweden. *Arthritis Res Ther* 2019; 21: 291.

681. Gumucio R, Azuaga AB, Isern-Kebschull J, et al. A Case Report of Exuberant Chondrocalcinosis of the Ankle: An Unusual Presentation. *J Clin Rheumatol Pract Rep Rheum Musculoskelet Dis* 2021; 27: e30–e31.

682. Guillot X, Tordi N, Laheurte C, et al. Local ice cryotherapy decreases synovial interleukin 6, interleukin 1β, vascular endothelial growth factor, prostaglandin-E2, and nuclear factor kappa B p65 in human knee arthritis: a controlled study. *Arthritis Res Ther* 2019; 21: 180.

683. Halupa AJ, Strony RJ, Bulbin DH, et al. Pseudogout Diagnosed By Point-of-care Ultrasound. *Clin Pract Cases Emerg Med* 2019; 3: 425–427.

684. Hakozaki M, Sekine T, Otani K, et al. Acute pseudogout lumbar discitis resembling acute pyelonephritis in an elderly woman. *Intern Med J* 2019; 49: 1048–1050.

685. Hajri R, Hajdu SD, Hügle T, et al. Dual-Energy Computed Tomography for the Noninvasive Diagnosis of Coexisting Gout and Calcium Pyrophosphate Deposition Disease. *Arthritis Rheumatol Hoboken NJ* 2019; 71: 1392.

686. George MP, Ernste FC, Tande A, et al. Clinical Presentation, Management, and Prognosis of Pseudogout in Joint Arthroplasty: A Retrospective Cohort Study. *J Bone Jt Infect* 2019; 4: 20–26.

687. Galeano-Valle F, Vengoechea J, Galindo RJ. A rare mutation in hypophosphatasia: a case report of adult form and review of the literature. *Arch Endocrinol Metab* 2019; 63: 89–93.

688. Gama RM, Barkham N, Ward J, et al. Acute pseudogout - Measure serum magnesium. *Ann Clin Biochem* 2019; 56: 411–414.

689. García-Fontana C, Villa-Suárez JM, Andújar-Vera F, et al. Epidemiological, Clinical and Genetic Study of Hypophosphatasia in A Spanish Population: Identification of Two Novel Mutations in The Alpl Gene. *Sci Rep* 2019; 9: 9569.

690. Gao L, Oláh T, Cucchiarini M, et al. Asymptomatic focal calcium pyrophosphate crystal deposition within partially failed repair tissue after matrix-assisted autologous chondrocyte implantation. *Knee Surg Sports Traumatol Arthrosc Off J ESSKA* 2019; 27: 1939–1942.

691. Finkenstaedt T, Biswas R, Abeydeera NA, et al. Ultrashort Time to Echo Magnetic Resonance Evaluation of Calcium Pyrophosphate Crystal Deposition in Human Menisci. *Invest Radiol* 2019; 54: 349–355.

692. Ellis JM. Acute monoarthritis. *JAAPA Off J Am Acad Physician Assist* 2019; 32: 25–31.

693. Pascart T, Norberciak L, Legrand J, et al. Dual-energy computed tomography in calcium pyrophosphate deposition: initial clinical experience. *Osteoarthritis Cartilage* 2019; 27: 1309–1314.

694. Stamp LK, Anderson NG, Becce F, et al. Clinical Utility of Multi-Energy Spectral Photon-Counting Computed Tomography in Crystal Arthritis. *Arthritis Rheumatol Hoboken NJ* 2019; 71: 1158–1162.

695. Sahu KK, Mishra AK, Nandagopal D. Pseudogout: a rare cause of joint swelling during postoperative period. *BMJ Case Rep*; 12. Epub ahead of print 13 May 2019. DOI: 10.1136/bcr-2019-230089.

696. Scheldeman L, Van Hoydonck M, Vanheste R, et al. Crowned dens syndrome: a neurologist’s perspective. *Acta Neurol Belg* 2019; 119: 561–565.

697. Tedeschi SK. Issues in CPPD Nomenclature and Classification. *Curr Rheumatol Rep* 2019; 21: 49.

698. Thomas M, Forien M, Palazzo E, et al. Efficacy and tolerance of anakinra in acute calcium pyrophosphate crystal arthritis: a retrospective study of 33 cases. *Clin Rheumatol* 2019; 38: 425–430.

699. Turaga S, Thomas M, Savy L, et al. Pseudogout or pseudolymphoma? Calcium pyrophosphate deposition disease of the cervical spine: a rare presentation and literature review. *BMJ Case Rep*; 12. Epub ahead of print 2 December 2019. DOI: 10.1136/bcr-2019-231508.

700. Ujihara T, Yamamoto K, Kitaura T, et al. Calcium Pyrophosphate Deposition Disease Involving a Lumbar Facet Joint Following Urinary Tract Infection. *Intern Med Tokyo Jpn* 2019; 58: 1787–1789.

701. Urits I, Peck J, Chesteen G, et al. An acute presentation of cervical pain: Crowned dens syndrome. *J Clin Anesth* 2019; 58: 117–118.

702. Zell M, Zhang D, FitzGerald J. Diagnostic advances in synovial fluid analysis and radiographic identification for crystalline arthritis. *Curr Opin Rheumatol* 2019; 31: 134–143.

703. Vasishta S, Patel S. Elevated Procalcitonin in Acute Pseudogout Flare: A Case Report. *Cureus* 2019; 11: e4853.

704. Wang YH, Ho S. A Rare Case of Gout and Pseudogout Occurring in the Same Joint. *Malays Orthop J* 2019; 13: 54–56.

705. Wang Y, Wei J, Zeng C, et al. Association between chondrocalcinosis and osteoarthritis: A systematic review and meta-analysis. *Int J Rheum Dis* 2019; 22: 1175–1182.

706. Desmarais J, Chu C-Q. Utility of Anakinra in Acute Crystalline Diseases: A Retrospective Study Comparing a University Hospital with a Veterans Affairs Medical Center. *J Rheumatol* 2019; 46: 748–750.

707. Saylısoy S. A case of acute calcium pyrophosphate arthritis in two rare sites of involvement: The cervical facet and atlantoaxial joint. *Jt Dis Relat Surg* 2020; 31: 395–398.

708. Ojemolon PE, Edigin E, Annapureddy N, et al. A Case of Acute Neck Pain: The Crowned Dens Syndrome. *Cureus* 2020; 12: e9555.

709. Konig MF, Faller GT. Acute Bilateral Pseudogout of the Temporomandibular Joint. *Arthritis Rheumatol* 2020; 72: 1159.

710. Kimura R, Miyakoshi N, Kobayashi T, et al. Acute exacerbation of cervical myelopathy caused by pseudogout of the cervical ligamentum flavum after cervical spinal cord injury: a case report. *Spinal Cord Ser Cases* 2020; 6: 98.

711. McCarthy GM, Dunne A. Calcium crystals and auto-inflammation. *Rheumatology* 2020; 59: 247–248.

712. Abou-Foul AK, Saeed NR. Treatment of calcium pyrophosphate deposition in the temporomandibular joint with resection and simultaneous reconstruction using a custom joint prosthesis. *Oral Maxillofac Surg* 2020; 24: 235–238.

713. Hubert J, Beil FT, Rolvien T, et al. Cartilage calcification is associated with histological degeneration of the knee joint: a highly prevalent, age-independent systemic process. *Osteoarthritis Cartilage* 2020; 28: 1351–1361.

714. Aliste-Fernández M, San-José P, Aguadero V. White blood cell count and total protein concentration to predict the absence of microcrystals in synovial fluid. *Clin Biochem* 2020; 83: 81–85.

715. Andrés M, Sivera F, Pascual E. Progresses in the imaging of calcium pyrophosphate crystal disease. *Curr Opin Rheumatol* 2020; 32: 140–145.

716. Kleyer A, Knitza J, Schett G, et al. Calcium pyrophosphate deposition disease induced inflammatory back pain. *Rheumatol Oxf Engl* 2020; 59: 456.

717. Moon AS, Mabry S, Pittman JL. Calcium pyrophosphate deposition disease of the cervical and thoracolumbar spine: A report of two cases. *North Am Spine Soc J* 2020; 3: 100026.

718. Loro LL, Bjørnland T. Calcium pyrophosphate deposition disease: A case report with bilateral involvement of the temporomandibular joints and concurrence of psoriatic arthritis. *Clin Case Rep* 2020; 8: 640–643.

719. Awisat A, Rosner I, Rimar D, et al. Crowned dens syndrome, yet another rheumatic disease imposter. *Clin Rheumatol* 2020; 39: 571–574.

720. Bousson V, Bardin T, Zeitoun D, et al. Monosodium urate deposition in the articular cartilage and meniscus can mimic chondrocalcinosis. *Joint Bone Spine* 2020; 87: 95–96.

721. McCarron EP, Wilson J, Galkin S, et al. Crowned dens syndrome: an easily overlooked cause of fever and neck stiffness. *QJM Mon J Assoc Physicians* 2020; 113: 52–53.

722. Cai K, Fuller A, Hensey O, et al. Outcome domains reported in calcium pyrophosphate deposition studies: A scoping review by the OMERACT CPPD working group. *Semin Arthritis Rheum* 2020; 50: 719–727.

723. Catelli A, Venetucci P, Castaldo A, et al. Calcium pyrophosphate deposition disease: The role of imaging in their detection and in differential diagnosis of crystal arthropathies. *Radiol Case Rep* 2020; 15: 1773–1776.

724. Hsieh S-F, Wu S-Y, Hung Y-C, et al. Concurrence of Pigmented Villonodular Synovitis with Calcium Pyrophosphate Deposition in a Postacute Stroke Patient. *J Med Ultrasound* 2020; 28: 188–191.

725. Chang D-G, Park J-B, Jung H-Y, et al. Cervical myelopathy due to subaxial calcium pyrophosphate dihydrate (CPPD) deposition with simultaneous asymptomatic crowned dens syndrome: two case reports. *BMC Musculoskelet Disord* 2020; 21: 713.

726. Chernoff DJ, Barker JP, Wingerter SA, et al. Gout After Total Knee Arthroplasty. *Arthroplasty Today* 2020; 6: 278–282.

727. Chisari E, Yaghmour KM, Peat F, et al. Patients Presenting with a Hot, Swollen Joint: A Single-Centre Retrospective Analysis. *Curr Rheumatol Rev* 2020; 16: 38–42.

728. Choi DD, Smith D, Davis CM, et al. Arthroscopic diagnosis and medical management of calcium pyrophosphate deposition disease in the temporomandibular joint. *Int J Oral Maxillofac Surg* 2020; 49: 1618–1621.

729. Ciaffi J, Borlandelli E, Mancarella L, et al. Sclerochoroidal calcifications associated with early-onset calcium pyrophosphate deposition disease. *Clin Rheumatol* 2020; 39: 2825–2826.

730. Cipolletta E, Smerilli G, Mashadi Mirza R, et al. Sonographic assessment of calcium pyrophosphate deposition disease at wrist. A focus on the dorsal scapho-lunate ligament. *Joint Bone Spine* 2020; 87: 611–617.

731. Cipolletta E, Di Matteo A, Scanu A, et al. Biologics in the treatment of calcium pyrophosphate deposition disease: a systematic literature review. *Clin Exp Rheumatol* 2020; 38: 1001–1007.

732. Cipolletta E, Di Matteo A, Filippucci E, et al. Calcium Pyrophosphate Deposition Disease in a Patient with Familial Hypokalemia-Hypomagnesemia (Gitelman’s-Syndrome): A Case Report - CPPD in Gitelman’s syndrome. *Ultraschall Med Stuttg Ger 1980* 2020; 41: 695–697.

733. Collinot J-A, Pascart T, Budzik J-F, et al. Non-invasive characterization of intra-articular mineralization using dual-energy computed tomography. *Rheumatol Oxf Engl* 2020; 59: 3997–3998.

734. Conticini E, Negro A, Magnani L, et al. Gitelman syndrome associated with chondrocalcinosis and severe neuropathy: a novel heterozygous mutation in SLC12A3 gene. *Reumatismo* 2020; 72: 67–70.

735. Conticini E, Di Martino V, De Stefano R, et al. Crowned Dens Syndrome Presenting as Hemiplegia and Hypoesthesia. *J Clin Rheumatol Pract Rep Rheum Musculoskelet Dis* 2020; 26: e312–e313.

736. Zimmer V. Crowned dens syndrome. *Clin Case Rep* 2020; 8: 2088–2089.

737. Slouma M, Rahmouni S, Dhahri R, et al. Bicipitoradial bursitis: A diagnosis challenge! *Clin Case Rep* 2020; 8: 2265–2268.

738. Ziegeler K, Hermann S, Hermann KGA, et al. Dual-energy CT in the differentiation of crystal depositions of the wrist: does it have added value? *Skeletal Radiol* 2020; 49: 707–713.

739. Lu P-L, Niu Z-F, Qiu C-W, et al. Odontoid calcification and crowned dens syndrome: data from a Chinese center. *Neurol Res* 2020; 42: 930–935.

740. Nguyen C-D, Morel V, Pierache A, et al. Bone and joint complications in patients with hereditary hemochromatosis: a cross-sectional study of 93 patients. *Ther Adv Musculoskelet Dis* 2020; 12: 1759720X20939405.

741. Kenny G, MacMahon P, Dempsey P, et al. The need for computed tomography imaging to differentiate the crowned dens syndrome from vertebral osteomyelitis. *Scand J Rheumatol* 2020; 49: 249–250.

742. Miura K, Koda M, Abe T, et al. Thoracic myelopathy caused by calcification of the ligamentum flavum. *J Rural Med JRM* 2020; 15: 65–67.

743. Latourte A, Ea H-K, Frazier A, et al. Tocilizumab in symptomatic calcium pyrophosphate deposition disease: a pilot study. *Ann Rheum Dis* 2020; 79: 1126–1128.

744. Oduyale O, Bailey A, MacKenzie J, et al. Two Birds, One Stone-An Odd Case of Oligoarthritis. *Am J Med* 2020; 133: 679–681.

745. De Silva T, Rischin A. Crowned Dens Syndrome Illustrated by Dual Energy Computed Tomography Scan. *J Clin Rheumatol Pract Rep Rheum Musculoskelet Dis* 2020; 26: e293.

746. Singla A, Ryan A, Bennett DL, et al. Non-infectious thoracic discitis: A diagnostic and management dilemma. A report of two cases with review of the literature. *Clin Neurol Neurosurg* 2020; 190: 105648.

747. Hotokezaka Y, Hotokezaka H, Katayama I, et al. A case of tophaceous pseudogout of the temporomandibular joint extending into the cranium. *Oral Radiol* 2020; 36: 203–208.

748. He H, Wang Y, Yang Z, et al. Association between serum zinc and copper concentrations and copper/zinc ratio with the prevalence of knee chondrocalcinosis: a cross-sectional study. *BMC Musculoskelet Disord* 2020; 21: 97.

749. Hage S, Hage V, El-Khoury N, et al. Musculoskeletal disorders in hemodialysis patients: different disease clustering according to age and dialysis vintage. *Clin Rheumatol* 2020; 39: 533–539.

750. Greca I, Ben Gabr J, Perl A, et al. Trauma Induced Calcium Pyrophosphate Deposition Disease of the Lumbar Spine. *Case Rep Rheumatol* 2020; 2020: 3218350.

751. Guermazi A, Jarraya M, Lynch JA, et al. Reliability of a new scoring system for intraarticular mineralization of the knee: Boston University Calcium Knee Score (BUCKS). *Osteoarthritis Cartilage* 2020; 28: 802–810.

752. Simon S, Resch H. Treatment of hypophosphatasia. *Wien Med Wochenschr 1946* 2020; 170: 112–115.

753. Haikal A, Everist BM, Jetanalin P, et al. Cervical CT-Dependent Diagnosis of Crowned Dens Syndrome in Calcium Pyrophosphate Dihydrate Crystal Deposition Disease. *Am J Med* 2020; 133: e32–e37.

754. Gomez Serrano M, Anne Watson N, Selvadurai D. A description of unilateral conductive hearing loss from pseudogout: a case report and review of the literature. *JRSM Open* 2020; 11: 2054270419894818.

755. Gandikota G, Fakuda T, Finzel S. Computed tomography in rheumatology - From DECT to high-resolution peripheral quantitative CT. *Best Pract Res Clin Rheumatol* 2020; 34: 101641.

756. Foreman SC, Gersing AS, von Schacky CE, et al. Chondrocalcinosis is associated with increased knee joint degeneration over 4 years: data from the Osteoarthritis Initiative. *Osteoarthritis Cartilage* 2020; 28: 201–207.

757. Forlizzi JM, Ryan JM, Galow KE, et al. Acute pseudogout presenting as an exception to Musculoskeletal Infection Society criteria in total knee arthroplasty: a case report. *AME Case Rep* 2020; 4: 21.

758. Filippou G, Pascart T, Iagnocco A. Utility of Ultrasound and Dual Energy CT in Crystal Disease Diagnosis and Management. *Curr Rheumatol Rep* 2020; 22: 15.

759. Filippou G, Scanu A, Adinolfi A, et al. Criterion validity of ultrasound in the identification of calcium pyrophosphate crystal deposits at the knee: an OMERACT ultrasound study. *Ann Rheum Dis* 2021; 80: 261–267.

760. Filippucci E, Reginato AM, Thiele RG. Imaging of crystalline arthropathy in 2020. *Best Pract Res Clin Rheumatol* 2020; 34: 101595.

761. Falkowski AL, Jacobson JA, Kalia V, et al. Cartilage icing and chondrocalcinosis on knee radiographs in the differentiation between gout and calcium pyrophosphate deposition. *PloS One* 2020; 15: e0231508.

762. Dupré A, Collins M, Nocturne G, et al. Articular manifestations in patients with inflammatory bowel disease treated with vedolizumab. *Rheumatol Oxf Engl* 2020; 59: 3275–3283.

763. Pascart T, Falgayrac G, Norberciak L, et al. Dual-energy computed-tomography-based discrimination between basic calcium phosphate and calcium pyrophosphate crystal deposition in vivo. *Ther Adv Musculoskelet Dis* 2020; 12: 1759720X20936060.

764. Pascual E, Sivera F, Andres M. Mixed Crystal Disease: A Tale of 2 Crystals. *J Rheumatol* 2020; 47: 1158–1159.

765. Pastor S, Bernal J-A, Caño R, et al. Persistence of Crystals in Stored Synovial Fluid Samples. *J Rheumatol* 2020; 47: 1416–1423.

766. Persons B, Kissin EY. Scruples over Speckles. *J Med Ultrasound* 2020; 28: 179–180.

767. Salles JP. Hypophosphatasia: Biological and Clinical Aspects, Avenues for Therapy. *Clin Biochem Rev* 2020; 41: 13–27.

768. Quin K, Madhoun HM. Ultrasound as a Biomarker in Rheumatic Diseases. *Diagn Basel Switz*; 10. Epub ahead of print 10 November 2020. DOI: 10.3390/diagnostics10110933.

769. Ramonda R, Cristiani B, Oliviero F, et al. Severe Abdominal Pain as a Manifestation of Pseudogout in Pubic Symphysis. *J Clin Rheumatol Pract Rep Rheum Musculoskelet Dis* 2020; 26: e30–e31.

770. Sugimoto H, Hayashi T, Nakadomari S, et al. Delayed diagnosis of an upper cervical epidural abscess masked due to crowned dens syndrome. *BMJ Case Rep*; 13. Epub ahead of print 20 May 2020. DOI: 10.1136/bcr-2020-235126.

771. Sakellariou G, Scirè CA, Adinolfi A, et al. Differential Diagnosis of Inflammatory Arthropathies by Musculoskeletal Ultrasonography: A Systematic Literature Review. *Front Med* 2020; 7: 141.

772. Tang J, Li J, Wu C, et al. Report of four cases of crowned dens syndrome: Clinical presentation, CT findings and treatment. *Exp Ther Med* 2020; 20: 3853–3859.

773. Scarlini S, Cavallieri F, Fiorini M, et al. Idiopathic brain calcification in a patient with hereditary hemochromatosis. *BMC Neurol* 2020; 20: 113.

774. Tedeschi SK, Solomon DH, Yoshida K, et al. A prospective study of dual-energy CT scanning, US and X-ray in acute calcium pyrophosphate crystal arthritis. *Rheumatol Oxf Engl* 2020; 59: 900–903.

775. Thomas J, Ninan K. ‘Combining Old and New’: Conventional Radiography and DECT for Diagnosing CPPD. *J Clin Rheumatol Pract Rep Rheum Musculoskelet Dis* 2020; 26: e2–e3.

776. Zell M, Aung T, Kaldas M, et al. Calcium pyrophosphate crystal size and characteristics. *Osteoarthr Cartil Open* 2021; 3: 100133.

777. Zavisanos A, Hasenstein T, Meyr AJ. Level of Agreement with the Microscopic Analysis of Joint Aspirate for the Diagnosis of Gout in the Lower Extremity. *J Am Podiatr Med Assoc* 2020; 110: Article_6.

778. Yates KA, Yoshida K, Xu C, et al. Acute Calcium Pyrophosphate Crystal Arthritis Flare Rate and Risk Factors for Recurrence. *J Rheumatol* 2020; 47: 1261–1266.

779. Vanni D, Berjano P. Spinal pseudogout is a joker. Commentary on ‘Calcium pyrophosphate deposition disease of the cervical and thoracolumbar spine: A report of two cases’. *North Am Spine Soc J* 2020; 3: 100028.

780. Willems JH, Rassir R, Sierevelt IN, et al. There is no difference in postoperative pain, function and complications in patients with chondrocalcinosis in the outcome of total knee arthroplasty for end-stage osteoarthritis. *Knee Surg Sports Traumatol Arthrosc Off J ESSKA* 2020; 28: 2970–2979.

781. Willekens I, Fares A, Devos H, et al. Prevalence of chondrocalcinosis in the temporomandibular joint in patients with chondrocalcinosis of the knee or wrist. *Dento Maxillo Facial Radiol* 2020; 49: 20190450.

782. Chotard E, Blanchard A, Ostertag A, et al. Calcium pyrophosphate crystal deposition in a cohort of 57 patients with Gitelman syndrome. *Rheumatol Oxf Engl* 2022; 61: 2494–2503.

783. Dang RR, Noonan V, Chigurupati R, et al. Treatment of tophaceous pseudogout in the temporomandibular joint with resection and alloplastic reconstruction: a single-staged approach. *Oral Maxillofac Surg* 2022; 26: 505–509.

784. Kravchenko D, Karakostas P, Kuetting D, et al. The role of dual energy computed tomography in the differentiation of acute gout flares and acute calcium pyrophosphate crystal arthritis. *Clin Rheumatol* 2022; 41: 223–233.

785. Mank VMF, Goldstein E, Babb S, et al. 20 Years of Radiographic Imaging: Crystalline Deposits Causing Severe Arthropathy and Erosions. *Mil Med* 2021; usab129.

786. Kamalaksha S, Wilkinson L, Ghosh S. Crowned dens syndrome: a key differential for acute neck pain in the elderly. *N Z Med J* 2021; 134: 130–133.

787. Qin X, Hu X, Wang Q, et al. A rare acute neck pain cause that can have misdiagnosis or missed diagnosis-crowned dens syndrome: description of two cases and a literature analysis. *Quant Imaging Med Surg* 2021; 11: 4491–4496.

788. Altomare A, Corrado A, Maruotti N, et al. The role of Interleukin-1 receptor antagonist as a treatment option in calcium pyrophosphate crystal deposition disease. *Mol Biol Rep* 2021; 48: 4789–4796.

789. Aoki N, Miyagami T, Shikino K, et al. Polymyalgia Rheumatica in a Patient with Pseudogout and Dementia. *Am J Case Rep* 2021; 22: e933926.

790. Mazzoni D, Xiao Y, Loch-Wilkinson T, et al. Calcium pyrophosphate deposition disease of the spine mimicking septic arthritis. *Intern Med J* 2021; 51: 1357–1358.

791. Bamgboje AO, Mohandas N. Crowned Dens Syndrome Masquerading as Meningitis. *Cureus* 2021; 13: e12678.

792. Barge L, Gogna G. Crowned dens syndrome in a patient with severe secondary iron overload. *Intern Med J* 2021; 51: 1549–1550.

793. Bashir M, Sherman KA, Solomon DH, et al. Cardiovascular disease risk in calcium pyrophosphate deposition disease: A nationwide study of veterans. *Arthritis Care Res*. Epub ahead of print 14 September 2021. DOI: 10.1002/acr.24783.

794. Liao J-H, Huang K-C, Hsieh C-T, et al. Cervical myeloradiculopathy as an initial presentation of pseudogout. *Neurosci Riyadh Saudi Arab* 2021; 26: 93–96.

795. Bernabei I, Sayous Y, Raja AY, et al. Multi-energy photon-counting computed tomography versus other clinical imaging techniques for the identification of articular calcium crystal deposition. *Rheumatol Oxf Engl* 2021; 60: 2483–2485.

796. Maheswaranathan M, Houk JL, Range DE, et al. Clinical Image: Pseudogout of the temporomandibular joint. *ACR Open Rheumatol* 2021; 3: 464–465.

797. Brotherton T, Miller CS. Infective endocarditis initially manifesting as pseudogout. *Proc Bayl Univ Med Cent* 2021; 34: 496–497.

798. Budzik J-F, Marzin C, Legrand J, et al. Can Dual-Energy Computed Tomography Be Used to Identify Early Calcium Crystal Deposition in the Knees of Patients With Calcium Pyrophosphate Deposition? *Arthritis Rheumatol Hoboken NJ* 2021; 73: 687–692.

799. Mizumoto J. Crowned dens syndrome: Caution about asymptomatic calcification. *Cleve Clin J Med* 2021; 88: 204–205.

800. Cai K, Tedeschi SK. Review: Outcome measures in calcium pyrophosphate deposition. *Best Pract Res Clin Rheumatol* 2021; 35: 101724.

801. Cai K, Fuller A, Zhang Y, et al. Towards development of core domain sets for short term and long term studies of calcium pyrophosphate crystal deposition (CPPD) disease: A framework paper by the OMERACT CPPD working group. *Semin Arthritis Rheum* 2021; 51: 946–950.

802. Sirotti S, Gutierrez M, Pineda C, et al. Accuracy of synovial fluid analysis compared to histology for the identification of calcium pyrophosphate crystals: an ancillary study of the OMERACT US Working Group - CPPD subgroup. *Reumatismo* 2021; 73: 106–110.

803. Cenin DA, Freyer CW, Ligon CB, et al. Tacrolimus induced pseudogout following allogeneic hematopoietic cell transplant. *J Oncol Pharm Pract Off Publ Int Soc Oncol Pharm Pract* 2021; 27: 771–775.

804. Chakravorty A, Murambi RT, Cherukuri RKV. Intradural calcium pyrophosphate dihydrate deposition disease: case report. *J Neurosurg Spine* 2021; 1–4.

805. Cho YJ, Han SI, Lim S-C. Cryptococcal infection presenting as soft tissue abscess and arthritis: Case report. *Medicine (Baltimore)* 2021; 100: e26656.

806. Cipolletta E, Filippou G, Scirè CA, et al. The diagnostic value of conventional radiography and musculoskeletal ultrasonography in calcium pyrophosphate deposition disease: a systematic literature review and meta-analysis. *Osteoarthritis Cartilage* 2021; 29: 619–632.

807. Doaré E, Robin F, Racapé H, et al. Features and Outcomes of Microcrystalline Arthritis Treated by Biologics: A Retrospective Study. *Rheumatol Ther* 2021; 8: 1241–1253.

808. Rammanohar J, Zhang C, Sidhu AA. An unusual case of neck pain: crowned dens syndrome. *Rheumatol Oxf Engl* 2021; 61: 471.

809. Lo P-C, Yue C-T, Kung W-M. Lumbar Extradural Pseudogout Mass Manifesting as Radiculopathy: A Case Report. *J Multidiscip Healthc* 2021; 14: 1593–1598.

810. Slouma M, Gharsallah I. Bilateral subacromial-subdeltoid bursitis in elderly patients: a diagnostic challenge. *Clin Exp Rheumatol* 2021; 39 Suppl 128: 14–15.

811. Ziegeler K, Richter S-T, Hermann S, et al. Dual-energy CT collagen density mapping of wrist ligaments reveals tissue remodeling in CPPD patients: first results from a clinical cohort. *Skeletal Radiol* 2021; 50: 417–423.

812. Cudrici CD, Newman KA, Ferrante EA, et al. Multifocal calcific periarthritis with distinctive clinical and radiological features in patients with CD73 deficiency. *Rheumatol Oxf Engl* 2021; 61: 163–173.

813. Lu Y-H, Lin H-H, Chen H-Y, et al. Multilevel calcium pyrophosphate dihydrate deposition in cervical ligamentum flavum: clinical characteristics and imaging features. *BMC Musculoskelet Disord* 2021; 22: 929.

814. Kim ST, Tayar J, Fu S, et al. Newly developed pseudogout arthritis after therapy with MAGE-A4 directed TCR T cells responded to treatment with tocilizumab. *J Immunother Cancer*; 9. Epub ahead of print July 2021. DOI: 10.1136/jitc-2021-002716.

815. Dermawan JK, Goldblum A, Reith JD, et al. The Incidence and Significance of Calcium Pyrophosphate Dihydrate Deposits in Histologic Examinations of Total Hip, Knee, and Shoulder Joint Arthroplasties. *Arch Pathol Lab Med* 2021; 145: 1558–1563.

816. Okamoto T, Ikeya T, Fukuda K. Crowned Dens Syndrome Occurring after Endoscopic Submucosal Dissection for Early Gastric Cancer. *Case Rep Gastroenterol* 2021; 15: 22–27.

817. Hill AC, Al Asmar R, Olajide AA, et al. A Case of Pseudogout Following Zoledronic Acid Administration. *Cureus* 2021; 13: e15627.

818. Ham Y, Mack H, Colville D, et al. Gitelman syndrome and ectopic calcification in the retina and joints. *Clin Kidney J* 2021; 14: 2023–2028.

819. Grunz J-P, Gietzen CH, Grunz K, et al. Imaging of Carpal Instabilities. *ROFO Fortschr Geb Rontgenstr Nuklearmed* 2021; 193: 139–150.

820. Haller C, Dietrich T, Neumann T. Coincident cervical rheumatoid arthritis and CPP-D associated crowned dens syndrome. *Rheumatol Oxf Engl* 2021; 60: 2466.

821. Germann C, Galley J, Falkowski AL, et al. Ultra-high resolution 3D MRI for chondrocalcinosis detection in the knee-a prospective diagnostic accuracy study comparing 7-tesla and 3-tesla MRI with CT. *Eur Radiol* 2021; 31: 9436–9445.

822. Gewolb DP, Entezami P, German JW, et al. Rare presentation of intradural calcium pyrophosphate dihydrate crystal deposition. *Neuroradiology* 2021; 63: 1735–1737.

823. Goldman S, Matson A. A Pain in the Neck Diagnosis: Crowned Dens Syndrome. *R I Med J 2013* 2021; 104: 40.

824. Grassi A, Dal Fabbro G, Fini M, et al. Case Report: Anterior Cruciate Ligament Calcification in a Patient With Chondrocalcinosis: Micro-Computed Tomography Presentation. *Front Surg* 2021; 8: 680234.

825. Fuller A, Cai K, Filippou G, et al. Experience and impact of crystal pyrophosphate deposition (CPPD) from a patient and caregiver perspective: A qualitative exploration from the OMERACT CPPD working group. *Semin Arthritis Rheum* 2021; 51: 655–660.

826. Fuller A, Cai K, Diaz-Torne C, et al. Outcome domains reported by patients, caregivers, healthcare professionals and stakeholders for calcium pyrophosphate deposition (CPPD): A content analysis based on semi-structured qualitative interviews from the OMERACT CPPD working group. *Semin Arthritis Rheum* 2021; 51: 650–654.

827. Finkelstein D, Foremny G, Singer A, et al. Differential diagnosis of T2 hypointense masses in musculoskeletal MRI. *Skeletal Radiol* 2021; 50: 1981–1994.

828. Fitzgerald J, Dalbeth N. New advances in crystal arthritis. *Best Pract Res Clin Rheumatol* 2021; 35: 101733.

829. Filippou G, Filippucci E, Mandl P, et al. A critical review of the available evidence on the diagnosis and clinical features of CPPD: do we really need imaging? *Clin Rheumatol* 2021; 40: 2581–2592.

830. Filippou G, Scanu A, Adinolfi A, et al. The two faces of the same medal… or maybe not? Comparing osteoarthritis and calcium pyrophosphate deposition disease: a laboratory and ultrasonographic study. *Clin Exp Rheumatol* 2021; 39: 66–72.

831. Falsetti P, Conticini E, Baldi C, et al. Ultrasound evaluation of the scapholunate ligament and scapholunate joint space in patients with wrist complaints in a rheumatologic setting. *J Ultrason* 2021; 21: e105–e111.

832. Ea H-K, Gauffenic A, Nguyen QD, et al. Calcium Pyrophosphate Dihydrate Crystal Deposition in Gouty Tophi. *Arthritis Rheumatol Hoboken NJ* 2021; 73: 324–329.

833. Dumusc A, So A. Answer to Cipolletta et al. ‘Treatment of acute CPP crystal arthritis: What are we missing?’. Joint Bone Spine 2021;88:105217. *Joint Bone Spine* 2021; 88: 105223.

834. Dumusc A, Pazar Maldonado B, Benaim C, et al. Anakinra compared to prednisone in the treatment of acute CPPD crystal arthritis: A randomized controlled double-blinded pilot study. *Joint Bone Spine* 2021; 88: 105088.

835. Parperis K, Papachristodoulou E, Kakoullis L, et al. Management of calcium pyrophosphate crystal deposition disease: A systematic review. *Semin Arthritis Rheum* 2021; 51: 84–94.

836. Shiraishi Y, Kanzawa Y, Ishimaru N, et al. Iliopsoas Bursitis Related to Calcium Pyrophosphate Deposition Disease. *Intern Med Tokyo Jpn* 2021; 60: 2515–2516.

837. Pongmanee S, Kaensuk S, Suppagornmongkol W, et al. Symptomatic retro-odontoid pseudotumor causing calcium pyrophosphate dihydrate deposition combined with multilevel cervical spondylotic myelopathy. *Int J Surg Case Rep* 2021; 89: 106622.

838. Schwabl C, Taljanovic M, Widmann G, et al. Ultrasonography and dual-energy computed tomography: impact for the detection of gouty deposits. *Ultrason Seoul Korea* 2021; 40: 197–206.

839. Schroeder AN, Johnson SE, Sellon JL. Pseudogout flare after platelet-rich plasma injection: A case series. *PM R*. Epub ahead of print 17 September 2021. DOI: 10.1002/pmrj.12708.

840. Qureshi AM, Tariq S, Javed N, et al. One Joint Aspirate: Three Diagnoses. *Cureus* 2021; 13: e17714.

841. Stack J, McCarthy G. Calcium pyrophosphate deposition (CPPD) disease – Treatment options. *Best Pract Res Clin Rheumatol* 2021; 35: 101720.

842. Stücker S, Bollmann M, Garbers C, et al. The role of calcium crystals and their effect on osteoarthritis pathogenesis. *Best Pract Res Clin Rheumatol* 2021; 35: 101722.

843. Sullivan J, Pillinger MH, Toprover M. Chondrocalcinosis: Advances in Diagnostic Imaging. *Curr Rheumatol Rep* 2021; 23: 77.

844. Tagoe CE, Wang W, Wang S, et al. Association of anti-thyroid antibodies with radiographic knee osteoarthritis and chondrocalcinosis: a NHANES III study. *Ther Adv Musculoskelet Dis* 2021; 13: 1759720X211035199.

845. Tang T, Han F-G. Calcium pyrophosphate deposition disease of the temporomandibular joint invading the middle cranial fossa: Two case reports. *World J Clin Cases* 2021; 9: 2662–2670.

846. Tedeschi SK, Cai T, He Z, et al. Classifying Pseudogout Using Machine Learning Approaches With Electronic Health Record Data. *Arthritis Care Res* 2021; 73: 442–448.

847. Tedeschi SK, Pascart T, Latourte A, et al. Identifying Potential Classification Criteria for Calcium Pyrophosphate Deposition Disease: Item Generation and Item Reduction. *Arthritis Care Res*. Epub ahead of print 10 May 2021. DOI: 10.1002/acr.24619.

848. Tedeschi SK, Yoshida K, Huang W, et al. Confirming Prior and Identifying Novel Correlates of Acute Calcium Pyrophosphate Crystal Arthritis. *Arthritis Care Res*. Epub ahead of print 16 August 2021. DOI: 10.1002/acr.24770.

849. Rosenthal AK. Calcium pyrophosphate deposition and crowned dens syndrome. *Cleve Clin J Med* 2021; 88: 206–207.

850. Zamudio-Cuevas Y, Martínez-Nava GA, Martínez-Flores K, et al. Synovial fluid analysis for the enhanced clinical diagnosis of crystal arthropathies in a tertiary care institution. *Clin Rheumatol* 2021; 40: 3239–3246.

851. Yurube T, Iguchi T, Kinoshita K, et al. Upper Cervical Compression Myelopathy Caused by the Retro-Odontoid Pseudotumor With Degenerative Osteoarthritis and Calcium Pyrophosphate Dihydrate Disease: A Case Report and Literature Review. *Neurospine* 2021; 18: 903–913.

852. Williams CJ, Rosenthal AK. Pathogenesis of calcium pyrophosphate deposition disease. *Best Pract Res Clin Rheumatol* 2021; 35: 101718.

853. Jeong MG, Park BS, Son E-S, et al. Crowned dens syndrome as a rare cause of anterior neck pain after transurethral resection of the prostate: a case report. *J Yeungnam Med Sci*. Epub ahead of print 5 August 2022. DOI: 10.12701/jyms.2022.00388.

854. Abdelkefi I, Saidenberg-Kermanac’h N, Boissier M-C, et al. Crowned dens syndrome. *Joint Bone Spine* 2022; 89: 105288.

855. Huang P, Xu M, He X-Y. Crowned Dens Syndrome: A Case Report and Literature Review. *Front Med* 2021; 8: 528663.

856. Lee J, Cheah J, Paudel P. Crowned Dens Syndrome Presenting as Altered Mental Status. *Am J Med* 2022; 135: e366–e367.

857. Bougioukas L, Vicks E, Hale AJ, et al. Rat bite fever in a patient with human immunodeficiency virus. *IDCases* 2022; 29: e01526.

858. Bustamante S, Boin M, Dankert J, et al. The utility of routine cultures, cell count, and crystal evaluation of aspirate from aseptic olecranon bursitis. *JSES Int* 2022; 6: 709–712.

859. Cadiou S, Le Gruyer A, Giguet B, et al. Calcium pyrophosphate deposition (CPPD) in a liver transplant patient: are hypomagnesemia, tacrolimus or both guilty? A case-based literature review. *Rheumatol Int* 2022; 42: 1105–1112.

860. Perozzo FAG, Punzi L, Costa AL, et al. Acute Calcium Pyrophosphate Crystal Arthritis of the Wrist Elicited by Anti-COVID-19 Vaccination After Carpal Tunnel Release. *Am J Case Rep* 2022; 23: e934833.

861. Krekeler M, Baraliakos X, Tsiami S, et al. High prevalence of chondrocalcinosis and frequent comorbidity with calcium pyrophosphate deposition disease in patients with seronegative rheumatoid arthritis. *RMD Open*; 8. Epub ahead of print June 2022. DOI: 10.1136/rmdopen-2022-002383.

862. Cipolletta E, Filippucci E, Abhishek A, et al. In patients with acute mono-oligoarthritis, a targeted ultrasound scanning protocol shows great accuracy for the diagnosis of gout and CPPD. *Rheumatol Oxf Engl* 2022; keac479.

863. Cipolletta E, Di Matteo A, Smerilli G, et al. Ultrasound findings of calcium pyrophosphate deposition disease at metacarpophalangeal joints. *Rheumatol Oxf Engl* 2022; keac063.

864. Liew JW, Peloquin C, Tedeschi SK, et al. Proton-Pump Inhibitors and Risk of Calcium Pyrophosphate Deposition in a Population-Based Study. *Arthritis Care Res*. Epub ahead of print 4 March 2022. DOI: 10.1002/acr.24876.

865. Nogueira Gomes T, Camelo Pereira M, Pinheiro Alves A, et al. Crowned Dens Syndrome: A Rare Complication of Calcium Pyrophosphate Crystal Deposition Disease. *Cureus* 2022; 14: e25593.

866. Muller R, Bernit E, Ebbo M, et al. Unusual calcium pyrophosphate deposition disease. *Rheumatol Oxf Engl* 2022; 61: 1297.

867. Ohyama Y, Yazawa M, Haji Y, et al. Calcium pyrophosphate deposition disease involving ‘the largest’ Bursa in the human body. *J Nephrol* 2022; 35: 687–688.

868. Parmar MS, Muppidi V, Bashir K. Gitelman Syndrome. In: *StatPearls*. Treasure Island (FL): StatPearls Publishing, 2022.

869. Haas P, Hauser T-K, Kandilaris K, et al. Case Report: Posterolateral Epidural Supra-C2-Root Approach (PESCA) for Biopsy of a Retro-Odontoid Lesions in Same Sitting After Occipitocervical Fixation and Decompression in a Case of Crowned Dens Syndrome With Brainstem Compression and Displacement. *Front Surg* 2022; 9: 797495.

870. Geneva-Popova M, Popova-Belova S, Popova V, et al. Assessment of Crystals in the Synovial Fluid of Psoriatic Arthritis Patients in Relation to Disease Activity. *Diagn Basel Switz*; 12. Epub ahead of print 18 May 2022. DOI: 10.3390/diagnostics12051260.

871. Francia A, Conte G, Platania G, et al. Teaching Neuroimage: Crowned Dens Syndrome, an Acute Attack of Calcium Pyrophosphate Deposition Disease Mimicking Acute Meningitis. *Neurology* 2022; 10.1212/WNL.0000000000200949.

872. Drosos AA, Pelechas E, Voulgari PV. A Patient with Symmetrical Polyarthritis. The Value of Conventional Radiography for a Correct Diagnosis. *Rheumatol Ther* 2022; 9: 771–779.

873. Parperis K, Hadi M, Bhattarai B. Outcomes and resource utilization in calcium pyrophosphate deposition disease patients who underwent total knee arthroplasty: a cross-sectional analysis. *Clin Rheumatol* 2022; 41: 1817–1824.

874. Shams S, Khan B, Jeffries A. Crowned dens syndrome-case of crystal deposition in cervical spine. *Oxf Med Case Rep* 2022; 2022: omab135.

875. Pfeil A. Calcium pyrophosphate crystal deposition in Gitelman syndrome: which joint is affected? *Rheumatol Oxf Engl* 2022; 61: 2211–2212.

876. Reijnierse M, Schwabl C, Klauser A. Imaging of Crystal Disorders:: Calcium Pyrophosphate Dihydrate Crystal Deposition Disease, Calcium Hydroxyapatite Crystal Deposition Disease and Gout Pathophysiology, Imaging, and Diagnosis. *Radiol Clin North Am* 2022; 60: 641–656.

877. Takeda K, Miyamoto I, Abe R, et al. Tophaceous pseudogout of the temporomandibular joint extending into the cranium: a case report with literature review. *J Surg Case Rep* 2022; 2022: rjac055.

878. Tantillo TJ, Chang K, Tan S, et al. An Unusual Pathologic Ulna Fracture Induced by Intraosseous Tumoral Calcium Pyrophosphate Dihydrate Crystal Deposition Disease. *J Hand Surg Glob Online* 2022; 4: 233–238.

879. Tedeschi SK, Becce F, Pascart T, et al. Imaging features of calcium pyrophosphate deposition (CPPD) disease: consensus definitions from an international multidisciplinary working group. *Arthritis Care Res*. Epub ahead of print 19 April 2022. DOI: 10.1002/acr.24898.

880. Tedeschi SK, Huang W, Yoshida K, et al. Risk of cardiovascular events in patients having had acute calcium pyrophosphate crystal arthritis. *Ann Rheum Dis* 2022; annrheumdis-2022-222387.

881. Terauchi M, Uo M, Fukawa Y, et al. Chemical Diagnosis of Calcium Pyrophosphate Deposition Disease of the Temporomandibular Joint: A Case Report. *Diagn Basel Switz*; 12. Epub ahead of print 7 March 2022. DOI: 10.3390/diagnostics12030651.

882. Zeng L, Liao H, Xie M, et al. Images in Neuroscience: Crowned Dens Syndrome. *Neurol India* 2022; 70: 1329–1330.

883. Zelano L, Locantore P, Rota CA, et al. Parathyroid Carcinoma All-In-One, a Rare Life-Threatening Case With Multiple Systemic Manifestations: Case Report and Review of the Literature. *Front Endocrinol* 2022; 13: 881225.

884. Yamauchi R, Ohta R, Igarashi M, et al. Pseudogout as a Cause of Fever of Unknown Origin Following Staphylococcal Bacteremia in an Older Patient. *Cureus* 2022; 14: e24333.

885. Wireko FW, Khalafalla S, Jamshidi T, et al. Septic and Crystal-Induced Arthritis (Pseudogout) Post-COVID-19 Vaccination. *Cureus* 2022; 14: e23902.

886. Weaver JS, Omar I, Mar W, et al. Magnetic resonance imaging of rheumatological diseases. *Pol J Radiol* 2022; 87: e93–e112.
