# Supplementary material for: Systematic literature review on Calcium Pyrophosphate Deposition (CPPD) nomenclature: condition elements and clinical states— A Gout, Hyperuricaemia and Crystal-Associated Disease Network (G-CAN) consensus project
Source: RMD Open. 2025 Jan 30;11(1):e004847. doi: 10.1136/rmdopen-2024-004847 (PMC11784236; doi:10.1136/rmdopen-2024-004847)
Supplement: online supplemental table 4 [file rmdopen-11-1-s004.docx]

**S4. General data on the 886 included articles.**

| **Article type** | **Articles, N (%)** | **Patient in included article (min-max)** | **Basis for CPPD diagnosis, N (%)** | | | | | **Pathogenic condition and pathogenic crystal names, and the labels used to represent them, N (%)** | | | | | **Basic condition elements, N (%)** | | **Clinical condition states, N (%)** | |
| --- | --- | --- | --- | --- | --- | --- | --- | --- | --- | --- | --- | --- | --- | --- | --- | --- |
|  |  |  | Mc Carty criteria | SFA | Imaging | Histology | Expert  Opinion | Patho-genic condition | Pathogenic condition abbreviation | Pathogenic condition abbreviation meaning | Pathogenic crystal label | Pathogenic crystal abbreviation | Crystal deposition | Imaging | Asymptomatic condition states | Symptomatic condition states |
| **All included articles** | 886 | 1-109,753 | 28 (3.2) | 272 (30.7) | 244 (27.5) | 160 (18.1) | 33 (3.7) | 783 (88.4) | 390 (44.0) | 421 (47.5) | 717 (80.9) | 550 (62.1) | 355 (40.1) | 569 (64.2) | 38 (4.3) | 460 (51.9) |
| **Case reports** | 394 (44.5) | 1 | 7 (1.8) | 117 (29.7) | 123 (31.2) | 106 (26.9) | 11 (1.2) | 342 (86.8) | 145 (36.8) | 176 (44.7) | 305 (77.4) | 202 (51.3) | 203 (51.5) | 284 (72.1) | 12 (3.0) | 242 (61.4) |
| **Scoping review** | 169 (19.0) | *NA* | 4 (2.4) | 39 (23.1) | 21 (12.4) | 8 (4.7) | 1 (0.6) | 148 (87.6) | 84 (49.7) | 84 (49.7) | 147 (87.0) | 120 (71.9) | 52 (30.8) | 102 (60.4) | 15 (8.9) | 85 (50.3) |
| **Cross-sectional cohort study** | 72 (8.1) | 6-25,157 | 0 (0.0) | 25 (34.7) | 19 (26.4) | 11 (15.3) | 0 (0.0) | 67 (93.1) | 31 (43.1) | 38 (52.8) | 60 (83.3) | 52 (72.2) | 19 (26.4) | 39 (54.2) | 3 (4.2) | 27 (37.5) |
| **Case series** | 58 (6.5) | 2-21 | 0 (0.0) | 20 (34.5) | 21 (36.2) | 20 (34.5) | 4 (6.9) | 49 (84.5) | 23 (39.7) | 29 (50.0) | 46 (79.3) | 37 (63.8) | 25 (43.1) | 38 (65.5) | 2 (3.4) | 34 (58.6) |
| **Retrospective cohort study** | 55 (6.2) | 22-109,753 | 0 (0.0) | 13 (23.6) | 9 (16.4) | 4 (7.3) | 1 (1.8) | 52 (9.5) | 31 (56.4) | 34 (61.8) | 42 (76.4) | 37 (67.3) | 16 (29.1) | 25 (45.4) | 2 (3.6) | 26 (47.2) |
| **Systematic literature review** | 35 (4.0) | *NA* | 1 (2.9) | 14 (40.0) | 13 (37.1) | 4 (11.4) | 3 (8.6) | 33 (94.3) | 19 (54.3) | 13 (37.1) | 29 (82.9) | 23 (65.7) | 10 (28.6) | 18 (51.4) | 2 (5.7) | 15 (42.9) |
| **Longitudinal cohort study** | 29 (3.3) | 5-4,543 | 2 (6.9) | 11 (37.9) | 9 (31.0) | 2 (6.9) | 3 (10.3) | 28 (96.6) | 13 (44.8) | 15 (51.7) | 26 (89.7) | 22 (75.9) | 10 (34.5) | 18 (62.1) | 0 (0.0) | 11 (37.9) |
| **Prospective case-control study** | 28 (3.2) | 4-162,204 | 6 (21.4) | 8 (28.6) | 6 (21.4) | 3 (10.7) | 2 (7.1) | 23 (82.1) | 18 (64.3) | 14 (50.0) | 25 (89.3) | 23 (82.1) | 11 (39.3) | 22 (78.6) | 1 (3.6) | 9 (32.1) |
| **Letter to the editor:**  **7 case series**  **3 case reports**  **2 cross-sectional cohort studies**  **3 scoping reviews**  **1 reply to a paper** | 16 (1.8) | 1-130 | 0 (0.0) | 2 (12.5) | 3 (18.8) | 1 (6.3) | 0 (0.0) | 14 (87.5) | 3 (18.8) | 5 (31.3) | 10 (62.5) | 9 (56.3) | 3 (18.8) | 8 (50.0) | 0 (0.0) | 3 (18.8) |
| **Retrospective case-control study** | 15 (1.7) | 28-10,024 | 3 (20.0) | 2 (13.3) | 3 (20.0) | 0 (0.0) | 4 (26.7) | 13 (86.7) | 12 (80.0) | 7 (46.7) | 14 (93.3) | 14 (93.3) | 2 (13.3) | 7 (46.7) | 1 (6.7) | 4 (26.7) |
| **Longitudinal case-control study** | 8 (0.9) | 40-205 | 1 (12.5) | 5 (62.5) | 3 (37.5) | 0 (0.0) | 0 (0.0) | 7 (87.5) | 5 (62.5) | 2 (25.0) | 6 (75.0) | 5 (62.5) | 3 (37.5) | 3 (37.5) | 0 (0.0) | 0 (0.0) |
| **Randomized controlled trial** | 4 (0.5) | 15-61 | 1 (25.0) | 2 (50.0) | 1 (25.0) | 0 (0.0) | 0 (0.0) | 4 (100.0) | 3 (75.0) | 1 (25.0) | 4 (100.0) | 3 (75.0) | 0 (0.0) | 2 (50.0) | 0 (0.0) | 2 (50.0) |
| **Meta-analysis** | 3 (0.3) | 597 | 2 (66.7) | 0 (0.0) | 0 (0.0) | 0 (0.0) | 0 (0.0) | 3 (100.0) | 3 (100.0) | 3 (100.0) | 3 (100.0) | 3 (100.0) | 1 (33.3) | 3 (100.0) | 0 (0.0) | 2 (66.7) |

*Legend: SFA: synovial fluid analysis; NA: non-applicable; N (%): effective (proportion); CPPD: calcium pyrophosphate deposition.*
