# Supplementary material for: Systematic literature review on Calcium Pyrophosphate Deposition (CPPD) nomenclature: condition elements and clinical states— A Gout, Hyperuricaemia and Crystal-Associated Disease Network (G-CAN) consensus project
Source: RMD Open. 2025 Jan 30;11(1):e004847. doi: 10.1136/rmdopen-2024-004847 (PMC11784236; doi:10.1136/rmdopen-2024-004847)
Supplement: online supplemental table 6 [file rmdopen-11-1-s006.pdf]

Supplementary Table 6. Correspondence between abbreviation and abbreviation meaning, regarding the crystal.

Abbreviation, Meaning	Number
**(none)**	**179**
calcium	1
calcium phosphate	1
calcium pyrophosphate	87
calcium pyrophosphate dehydrate	12
calcium pyrophosphate dihydrate	74
inorganic pyrophosphate	1
pyrophosphate	1
pyrophosphate calcium	1
pyrophosphate dihydrate	1
**CCP**	**3**
calcium pyrophosphate	3
**CCPD**	**1**
calcium pyrophosphate dihydrate	1
**CP**	**1**
calcium pyrophosphate	1
**CPC**	**1**
calcium pyrophosphate	1
**CPDD**	**1**
calcium pyrophosphate dihydrate	1
**CPP**	**221**
(none)	3
calcium phosphate	1
calcium pyrophosphate	180
calcium pyrophosphate dehydrate	4
calcium pyrophosphate dihydrate	32
pyrophosphate	1
**CPPD**	**350**
(none)	10
ca2+ pyrophosphate dihydrate	1
calcium	1
calcium phosphate	1
calcium pyrophosphatase dihydrate	1
calcium pyrophosphate	15
calcium pyrophosphate dehydrate	45
calcium pyrophosphate dihydrate	273
pyrophosphate	2
pyrophosphate dihydrate	1
**CPPDC**	**1**
calcium inorganic pyrophosphate dihydrate	1
**CPPDD**	**1**
calcium pyrophosphate dihydrate	1
**PPi**	**1**
inorganic pyrophosphate	1
**SCPPD**	**1**
calcium pyrophosphate	1
**T-CPPD**	**1**
triclinic calcium pyrophosphate dihydrate	1
