# Supplementary material for: Systematic literature review on Calcium Pyrophosphate Deposition (CPPD) nomenclature: condition elements and clinical states— A Gout, Hyperuricaemia and Crystal-Associated Disease Network (G-CAN) consensus project
Source: RMD Open. 2025 Jan 30;11(1):e004847. doi: 10.1136/rmdopen-2024-004847 (PMC11784236; doi:10.1136/rmdopen-2024-004847)
Supplement: online supplemental table 7 [file rmdopen-11-1-s007.pdf]

Supplementary tables: crude results

Supplementary Table S7. Labels used to represent the concepts of pathogenic condition (name, abbreviation, and abbreviation meaning) and pathogenic crystal (name and abbreviation), among all article types, between 2000 and 2022

Pathogenic condition name, N	Pathogenic condition abbreviation, N	Pathogenic condition abbreviation meaning, N	Pathogenic crystal name, N	Pathogenic crystal abbreviation, N
Pseudogout, 365	CPPD, 312	Calcium pyrophosphate deposition disease, 104	Calcium pyrophosphate dihydrate, 372	CPPD, 334
Chondrocalcinosis, 207	CPDD, 39	Calcium pyrophosphate dihydrate, 73	Calcium pyrophosphate, 280	CPP, 214
Calcium pyrophosphate deposition disease, 181	CC, 31	Calcium pyrophosphate deposition, 72	Calcium pyrophosphate dehydrate, 61	CCP, 3
Calcium pyrophosphate dihydrate crystal deposition disease, 105	CPPD-CDD, 9	Calcium pyrophosphate dihydrate deposition disease, 28	Pyrophosphate, 4	CCPD, 1
Calcium pyrophosphate dihydrate deposition disease, 95	CPPDD, 6	Calcium pyrophosphate dihydrate crystal deposition disease, 26	Calcium phosphate, 3	CP, 1
Calcium pyrophosphate deposition, 29	CPPDCD, 4	Chondrocalcinosis, 24	Pyrophosphate dihydrate, 2	CPC, 1
Pyrophosphate arthropathy, 26	CPPDDD, 2	Calcium pyrophosphate dihydrate deposition, 23	Calcium pyrophosphate in dihydrate form, 1	CPDD, 1
Calcium pyrophosphate dihydrate disease, 21	TCPPD, 2	Calcium pyrophosphate crystal deposition, 21	Calcium inorganic pyrophosphate dihydrate, 1	CPPDC, 1
Calcium pyrophosphate dihydrate deposition, 21	CPP, 2	Calcium pyrophosphate dehydrate, 13	Calcium-containing crystal, 1	CPPDD, 1
Calcium pyrophosphate dihydrate crystal deposition, 20	CCA, 1	Calcium pyrophosphate dihydrate crystal deposition, 9	Pyrophosphate calcium, 1	CPPS, 1
Calcium pyrophosphate crystal deposition disease, 18	CCAL, 1	Calcium pyrophosphate disease, 8	Triclinic calcium pyrophosphate dihydrate, 1	SCPPD, 1
Calcium pyrophosphate dehydrate crystal deposition disease, 13	CCPD, 1	Calcium pyrophosphate crystal deposition disease, 7		T-CPPD, 1
Calcium pyrophosphate disease, 13	CPP-CA, 1	Calcium pyrophosphate dehydrate crystal deposition disease, 5		
Calcium pyrophosphate crystal deposition, 12	CPPA, 1	Calcium pyrophosphate, 3		
Calcium pyrophosphate dihydrate arthropathy, 10	SCPPD, 1	Deposition of calcium pyrophosphate dihydrate, 2		
Calcium pyrophosphate dehydrate deposition disease, 9	TCPPDCD, 1	Crystal pyrophosphate deposition disease, 1		
Calcium pyrophosphate crystal arthritis, 9		Crystal pyrophosphate dihydrate deposition disease, 1		
Calcium pyrophosphate deposition arthropathy, 5		Calcium pyrophosphate dihydrate deposition disorder, 1		
Calcium pyrophosphate arthropathy, 5		Calcium pyrophosphate dihydrate disease, 1		
Calcium pyrophosphate dehydrate deposition, 4		Calcium pyrophosphate dehydrate deposition disease, 1		
Calcium pyrophosphate dihydrate 4		Calcium pyrophosphate crystal disease, 1		
Calcium pyrophosphate dehydrate arthropathy, 3		Calcium pyrophosphate dehydrate deposition, 1		
Calcium pyrophosphate crystal disease, 3		Calcium pyrophosphate crystal arthritis, 1		
Tophaceous pseudogout, 2		Calcium pyrophosphate dihydrate crystal arthropathy, 1		
Calcium pyrophosphate dehydrate crystal deposition, 2		Calcium pyrophosphate dihydrate crystal deposition arthropathy, 1		
Calcium pyrophosphate dihydrate deposition disorder, 2		Calcium pyrophosphate dihydrate crystal, 1		
Calcium pyrophosphate dihydrate crystal disease, 2		Calcium pyrophosphate dihydrate crystal deposits, 1		
CPPD crystal deposition disease, 2		Calcium pyrophosphate in dihydrate form, 1		
Calcium pyrophosphate dihydrate crystal arthropathy, 2		Primary calcium pyrophosphate disease, 1		
Calcium pyrophosphate dihydrate crystal-related arthropathy, 2		Tophaceous CPPD, 1		
Calcium pyrophosphate dihydrate deposition arthropathy, 2		Tumoral calcium pyrophosphate dihydrate deposition disease, 1		
Calcium pyrophosphate microcrystal deposition disease, 1		Tumoural CPPD crystal deposition disease, 1		
Calcium pyrophosphate in dihydrate form crystal deposition disease, 1				
Calcium pyrophosphate dihydrate associated disease, 1				
Calcium pyrophosphate dihydrate disorder, 1				
Crystal pyrophosphate deposition disease, 1				
Pyrophosphate calcium dihydrate disease, 1				
Pyrophosphate crystal deposition disease, 1				
Calcium pyrophosphate-related pseudogout, 1				
Pyrophosphate chondrocalcinosis, 1				
Arthropathy associated with calcium pyrophosphate crystal deposition, 1				
Calcium arthropathy, 1				
Calcium crystal-associated arthropathy, 1				
Calcium pyrophosphate dihydrate related arthropathy, 1				
Calcium pyrophosphate dihydrate crystal deposition arthropathy, 1				
Calcium pyrophosphate dihydrates crystal deposition disease arthropathy, 1				

Calcium pyrophosphate crystal arthropathy, 1<br>Calcium pyrophosphate crystal deposition related arthropathy, 1<br>Calcium pyrophosphate deposition disease arthropathy, 1<br>Calcium pyrophosphate-related arthropathy, 1<br>Chronic arthropathy, 1<br>Chronic pyrophosphate arthropathy, 1<br>CPPD arthropathy, 1<br>Calcium pyrophosphate associated arthritis, 1<br>Calcium pyrophosphate dehydrate arthritis, 1<br>Calcium pyrophosphate dihydrate arthritis, 1<br>Calcium pyrophosphate dihydrate induced arthritis, 1<br>Calcium pyrophosphate deposition arthritis, 1<br>Gout-like arthritis, 1<br>Calcium pyrophosphate dihydrate crystal deposits, 1<br>Deposition of calcium pyrophosphate dehydrate, 1<br>Deposition of calcium pyrophosphate dihydrate, 1<br>Deposition of calcium pyrophosphate dihydrate crystals, 1<br>Pyrophosphate gout, 1				
