# Supplementary material for: Systematic literature review on Calcium Pyrophosphate Deposition (CPPD) nomenclature: condition elements and clinical states— A Gout, Hyperuricaemia and Crystal-Associated Disease Network (G-CAN) consensus project
Source: RMD Open. 2025 Jan 30;11(1):e004847. doi: 10.1136/rmdopen-2024-004847 (PMC11784236; doi:10.1136/rmdopen-2024-004847)
Supplement: online supplemental table 8 [file rmdopen-11-1-s008.pdf]

Supplementary tables: crude results

Supplementary Table S8. Labels used to represent chemical and imaging basic condition elements, among all article types, between 2000 and 2022

The macroscopic deposition of pathogenic crystal in tissues	The microscopic deposition of pathogenic crystal in tissues	The presence of pathogenic crystal in SFA	Evidence of pathogenic crystal deposition on CR	Evidence of pathogenic crystal deposition on US	Evidence of pathogenic crystal deposition on CT	Evidence of pathogenic crystal deposition on MRI	Evidence of pathogenic crystal deposition on DECT
Material, 23<br>Mass, 21<br>Deposit, 10<br>Tissue, 7<br>Lesion, 4<br>Substance, 3<br>Cyst, 3<br>Calcification, 2<br>Nodule, 2<br>Aggregate, 1<br>Body, 1<br>Collection, 1<br>Fragment, 1<br>Mass-forming, 1<br>Chondrocalcinosis, 1<br>Matter, 1<br>Ossification, 1<br>Spot, 1<br>Surface, 1<br>Tophus, 1	Crystal, 94<br>Deposit, 41<br>Calcification, 16<br>Material, 11<br>Chondrocalcinosis, 9<br>Aggregate, 3<br>Mass, 3<br>Nodule, 3<br>Granule, 2<br>Structure, 2<br>Tissue, 2<br>Area, 2<br>Accumulation, 1<br>Arrow, 1<br>Chondrocalcification, 1<br>Collection, 1<br>Formation, 1<br>Precipitation, 1<br>Projection, 1	Crystal, 210<br>Aggregate of crystal, 1<br>Deposition, 1<br>Chondrocalcinosis, 1<br>Particle, 1<br>Arrow, 1	Chondrocalcinosis, 285<br>Calcification, 139<br>Deposit, 17<br>Calcific deposit, 9<br>Calcium deposit, 7<br>Calcified mass, 6<br>Mass, 6<br>Crystal deposit, 4<br>Calcific density, 3<br>Calcified lesion, 3<br>Lesion, 2<br>Calcific mass, 1<br>Calcified material, 1<br>Calcified process, 1<br>Calcified synovial cyst, 1<br>Density, 1<br>Material, 1<br>Opacity, 1<br>Ossification, 1	Deposit, 68<br>Calcification, 15<br>Chondrocalcinosis, 7<br>Aggregate, 6<br>Nodule, 2<br>Bodies, 1<br>Calcific reflection, 1<br>Double contour, 1<br>Pseudo-double contour, 1<br>Lesion, 1<br>Mass, 1	Calcification, 122<br>Calcified mass, 29<br>Chondrocalcinosis, 19<br>Calcified lesion, 7<br>Mass, 6<br>Calcium deposit, 3<br>Calcified deposit, 3<br>Calcific deposit, 3<br>High density mass, 2<br>High-density, 2<br>Ossification, 2<br>Calcium deposition, 1<br>Calcific density, 1<br>Calcific substance, 1<br>Calcified area, 1<br>Calcified ligament, 1<br>Calcified material, 1<br>Calcified synovial cyst, 1<br>Cystic mass, 1<br>Dense deposit, 1<br>Deposit, 1<br>Destructive mass, 1<br>Fibro-osseous mass, 1<br>Hyperdense deposit, 1<br>Hyperdense soft tissue, 1<br>Hyperdensity, 1<br>Mineralised deposit, 1<br>Mineralized mass, 1<br>Ossified mass, 1<br>Radio-opaque density, 1<br>Radiodense material, 1<br>Radio-opacity, 1	Mass, 30<br>Calcification, 17<br>Chondrocalcinosis, 10<br>Lesion, 5<br>Calcified mass, 4<br>Cystic mass, 2<br>Calcific deposit, 1<br>Calcified cyst, 1<br>Calcified lesion, 1<br>Calcium deposit, 1<br>Cyst formation, 1<br>Cystic collection, 1<br>Cystic tumor, 1<br>Pannus, 1<br>Pseudo-tumor, 1<br>Soft tissue thickening, 1	Calcification, 5<br>Calcium deposition, 2<br>Calcium pyrophosphate crystal deposition, 1<br>Calcium pyrophosphate deposition, 1<br>Calcium pyrophosphate crystal deposit, 1<br>Chondrocalcinosis, 1<br>Image of color-coded calcium deposition, 1<br>Hyperdensity, 1<br>Specific DECT attenuation properties, 1
