# Supplementary material for: Systematic literature review on Calcium Pyrophosphate Deposition (CPPD) nomenclature: condition elements and clinical states— A Gout, Hyperuricaemia and Crystal-Associated Disease Network (G-CAN) consensus project
Source: RMD Open. 2025 Jan 30;11(1):e004847. doi: 10.1136/rmdopen-2024-004847 (PMC11784236; doi:10.1136/rmdopen-2024-004847)
Supplement: online supplemental table 9 [file rmdopen-11-1-s009.pdf]

Supplementary tables: crude results

Supplementary Table S9. Labels used to represent clinical condition states, among all article types, between 2000 and 2022

Asymptomatic condition states		
**Asymptomatic evidence of pathogenic crystal deposition on SFA**	**Asymptomatic evidence of pathogenic crystal deposition on CR**	**Asymptomatic evidence of pathogenic crystal deposition on CT scan**
Calcium pyrophosphate crystals in aspiration from asymptomatic joints, 2<br>Calcium pyrophosphate crystals in aspiration from asymptomatic joints during inter-critical periods, 1	Chondrocalcinosis, 15<br>Asymptomatic chondrocalcinosis, 11<br>Asymptomatic radiological finding, 1<br>Incidental radiological finding, 1<br>Asymptomatic calcification of the articular cartilage, 1<br>Asymptomatic calcium deposit, 1<br>Asymptomatic calcium pyrophosphate crystal deposition, 1<br>Asymptomatic calcium pyrophosphate dihydrate, 1<br>Asymptomatic calcium pyrophosphate dihydrate deposit, 1<br>Asymptomatic cartilage calcification, 1<br>Asymptomatic CPPD, 1<br>Asymptomatic CPPD deposition, 1<br>Crystal deposits in asymptomatic joint, 1<br>Incidental CPPD deposition, 1<br>Intra-articular calcification, 1<br>Lanthanic chondrocalcinosis, 1<br>Lanthanic CPPD, 1<br>Radiographically visible calcific deposits without symptoms, 1	Asymptomatic calcification 1<br>Asymptomatic calcium deposition 1

Symptomatic condition states						
Acute peripheral articular inflammation triggered by the presence of pathogenic crystals	Recurrent acute peripheral articular inflammation triggered by the presence of pathogenic crystals	Persistent peripheral articular inflammation triggered by the presence of pathogenic crystals	Skin and subcutaneous involvement with evidence of pathogenic crystal deposits	Cervical spinal involvement with evidence of pathogenic crystal deposits	Lumbar spinal involvement with evidence of pathogenic crystal deposits	Symptomatic OA related to pathogenic crystal deposits
Pseudogout, 113<br>Acute CPP crystal arthritis, 55<br>Acute pseudogout, 22<br>Acute arthritis, 21<br>Attack of pseudogout, 12<br>Acute attack, 10<br>Pseudogout attack, 7<br>Acute pseudogout attack, 5<br>Acute CPPD arthritis, 4<br>Acute CPPD disease, 4<br>Acute synovitis, 4<br>Acute arthropathy, 3<br>Attack of arthritis, 3<br>Acute flare, 2<br>Pseudogout flare, 2<br>Arthritis, 2<br>Acute CPDD, 2<br>Acute attack of arthritis, 2<br>Acute attack of synovitis, 2	Pseudogout, 16<br>Acute recurrent CPP arthritis, 4<br>Pseudo-rheumatoid arthritis, 3<br>Recurrent arthritis, 3<br>Pseudogout attacks, 3<br>Recurrent pseudogout, 3<br>Recurrent flares, 2<br>Relapsing-remitting CPP crystal arthritis, 2<br>Asynchronous arthritis, 1<br>Acute recurrent CPP crystal arthritis, 1	Chronic CPP crystal arthritis, 27<br>Pseudo-rheumatoid arthritis, 25<br>Chronic arthritis, 15<br>Chronic CPP arthritis, 2<br>Chronic CPPD arthritis, 2<br>Chronic CPPD disease, 2<br>Chronic CPP crystal inflammatory arthritis, 1<br>Chronic arthropathy, 1<br>Chronic calcium pyrophosphate crystal disease, 1<br>Chronic calcium pyrophosphate deposition disease, 1<br>Chronic calcium pyrophosphate dihydrate crystal deposition, 1	Tophaceous pseudogout, 25<br>Tophaceous CPPD, 5<br>Tumoral CPPD, 4<br>Tumoral calcium pyrophosphate deposition disease, 3<br>Tumoral CPPD crystal deposition disease, 1<br>Tumoural CPPD crystal deposition disease, 1<br>Tumoral CPPD disease, 1<br>Tumoral pseudogout, 1<br>Pseudotumoral CPPD, 1<br>Tophaceous CPPD disease, 1<br>Tumoural form of calcium	Crowned dens syndrome, 122<br>Cervical cord compression/myelopathy, 24<br>Cervical axial pain, 20<br>Cervical axial stiffness, 17<br>Calcium pyrophosphate dihydrate spinal deposition, 4<br>Spinal canal stenosis, 4<br>Spinal CPPD, 2<br>Axial calcium pyrophosphate dihydrate deposition disease, 2<br>Calcification of the ligamentum flavum, 2<br>Crystal-induced arthritis of the lateral atlantoaxial joint, 2<br>Acute atlantoaxial arthritis, 1<br>Acute inflammatory episodes of the spine related to CPP deposition, 1<br>Acute spinal pain mimicking spondylodiscitis, 1<br>Atlantoaxial chondrocalcinosis, 1<br>Attack of pseudogout in the atlantoaxial joint, 1<br>Axial localization of calcium pyrophosphate dihydrate arthropathy, 1<br>Calcification of the atlanto-occipital ligament, 1	Lumbar axial pain, 7<br>Radiculopathy, 6<br>Cauda equina syndrome, 3<br>Calcium pyrophosphate dihydrate spinal deposition, 1<br>Acute lumbar pseudogout, 1<br>Calcium pyrophosphate dihydrate deposition disease of the lumbar spine, 1<br>Calcium pyrophosphate dihydrate lumbar deposition, 1<br>Chronic pseudogout of the lumbar spine, 1<br>Lumbar axial stiffness, 1<br>Lumbar calcium pyrophosphate deposition disease, 1	CPPD-related/associated osteoarthritis, 37<br>Pseudo-osteoarthritis, 25<br>CPP crystals deposits with osteoarthritis, 15<br>Osteoarthritis with chondrocalcinosis, 14<br>Pyrophosphate arthropathy, 12<br>Osteoarthritis, 4<br>Destructive arthropathy, 3<br>Chronic CPP arthropathy associated with osteoarthritis, 2<br>Severe osteoarthritis, 2<br>Chronic CPP arthropathy, 1<br>Chronic destructive arthropathy, 1<br>Chronic recurrent arthropathy, 1<br>Chronic arthropathy, 1<br>Chronic arthropathy mimicking osteoarthritis, 1

Acute calcium pyrophosphate dihydrate disease, 2<br>Acute attack of CPPD arthritis, 1<br>Acute attack of CPPD crystal-induced synovitis, 1<br>Acute calcium pyrophosphate dihydrate arthritis, 1<br>Acute calcium pyrophosphate deposition, 1<br>Acute calcium pyrophosphate deposition arthropathy, 1<br>Acute calcium pyrophosphate deposition disease, 1<br>Acute calcium pyrophosphate disease, 1<br>Acute CPPD, 1<br>Acute CPPD-associated arthritis, 1<br>Acute episode, 1<br>Acute exacerbation of CPPD, 1<br>Acute gout-like attack, 1<br>Acute inflammation due to deposit of CPPD crystals, 1<br>Acute pseudogout flare, 1<br>Attacks of acute pseudogout, 1<br>Pseudogout arthritis, 1<br>Symptomatic pseudogout, 1	Intermittent flares, 1<br>Intermittent swelling, 1<br>Paroxysmal form, 1<br>Recurrent attacks of pseudogout, 1<br>Recurrent attacks of synovitis, 1<br>Recurrent CPPD, 1<br>Recurrent form, 1<br>Recurrent joint pain, 1<br>Recurrent swelling, 1	Chronic calcium pyrophosphate dihydrate deposition disease, 1<br>Chronic CPDD, 1<br>Chronic CPPD, 1<br>Chronic CPPD crystal arthritis, 1<br>Chronic CPPD deposition disease, 1<br>Chronic form, 1<br>Chronic inflammatory calcium pyrophosphate deposition, 1<br>Chronic pseudogout, 1<br>Chronic recurrent calcium pyrophosphate arthropathy, 1<br>CPPD's chronic form, 1<br>Persistent arthritis, 1<br>Persistent disease, 1<br>Persistent flare, 1<br>Persistent swelling, 1<br>Pseudo-osteoarthritis, 1<br>Refractory CPP crystal-induced inflammatory arthritis, 1<br>Steroid-resistant pseudogout, 1	pyrophosphate dihydrate deposition, 1	Calcifications along the transverse ligament of the atlas, 1<br>Calcium deposits in the atlantoaxial ligaments, 1<br>Calcium pyrophosphate deposition of cervical spine, 1<br>Calcium pyrophosphate dihydrate spinal deposition disease, 1<br>Cervical calcium pyrophosphate dihydrate deposition, 1<br>Cervical calcium pyrophosphate dihydrate spinal deposition, 1<br>Cervical chondrocalcinosis, 1<br>Cervical pseudogout, 1<br>Chondrocalcinosis of the atlanto-axial joint, 1<br>Chondrocalcinosis of the cruciform ligament, 1<br>Chondrocalcinosis surrounding the odontoid process, 1<br>CPP crystal-induced spondylodiscitis, 1<br>CPPD around the odontoid process, 1<br>CPPD attack of the cervical ligamentum flavum, 1<br>CPPD crystal deposition in the atlantoaxial joint, 1<br>CPPD crystals deposits in the ligamentum flavum or atlanto-occipital ligament, 1<br>CPPD in the transverse ligament of the atlas, 1<br>Crystal deposition in the cervical spine, 1<br>Foramen magnum syndrome, 1<br>Lateral atlantoaxial joint CPPD crystal-induced arthritis, 1<br>Ligamentum flavum calcifications, 1<br>Meningism, 1<br>Periodontoid calcium pyrophosphate dihydrate crystal deposition, 1<br>Pseudogout attack of the cervical ligamentum flavum 1<br>Pseudogout attack of the yellow ligament, 1<br>Pseudogout of the neck, 1<br>Pyrophosphate arthropathy of the atlantoaxial joint, 1<br>Radiculomyelopathy, 1<br>Retro-odontoid calcium pyrophosphate crystal deposition, 1<br>Retro-odontoid CPPD mass, 1<br>Retro-odontoid pseudo-tumor due to calcium pyrophosphate crystal deposits, 1<br>Retrodental inflammatory pannus, 1<br>Spinal calcium pyrophosphate dehydrate crystal deposition disease, 1<br>Tumoral CPPDD of the ligamentum flavum, 1	Lumbar myelopathy, 1<br>Lumbar pseudogout, 1<br>Lumbar spine chondrocalcinosis, 1<br>Pseudogout of the spine, 1<br>Sacroiliitis, 1<br>Spinal intracanal conflict with a CPPD lesion in the zygapophyseal joint, 1<br>Tophaceous pseudogout of the lumbar spine, 1	Chronic CPPD associated osteoarthritis, 1<br>Chronic pyrophosphate arthropathy, 1<br>Severe destructive arthropathy, 1<br>Severe arthropathy, 1<br>Chronic CPP arthropathy, 1<br>Chronic CPPD arthropathy, 1<br>Chronic CPPD-CDD with osteoarthritis, 1<br>Chronic CPPD-related arthropathy, 1<br>Chronic degenerative joint disease, 1<br>Chronic noninflammatory calcium pyrophosphate deposition, 1<br>Crystal-induced arthropathy, 1<br>Degenerative arthropathy, 1<br>Destructive arthropathy, 1<br>Early osteoarthritis, 1<br>Erosive arthropathy, 1<br>Erosive osteoarthritis, 1<br>Exaggerated form of osteoarthritis, 1<br>Inflammatory arthropathy, 1<br>Joint degeneration, 1<br>Joint destructive form of CPP crystal-induced arthritis, 1<br>Multifocal osteoarthritis, 1<br>Osteoarthritic changes with pseudogout, 1<br>Osteoarthritis at unusual sites, 1<br>Osteoarthritis with pseudogout, 1<br>Premature generalized aggressive destructive osteoarthritis, 1<br>Pseudo-neuroarthropathy, 1<br>Pseudo-neuropathic arthropathy, 1<br>Pseudo-osteoarthritis with chondrocalcinosis, 1<br>Pseudogout induced osteoarthritis, 1<br>Pseudogout with osteoarthritis, 1<br>Secondary osteoarthritis, 1
